# Cation–Anion Co‐doping Enables Acid‐Stable MnO_2_ Anodes for Proton Exchange Membrane Water Electrolysis

**DOI:** 10.1002/smsc.70388

**Published:** 2026-08-27

**Authors:** Vimanshu Chanda, Olaf Rüdiger, Daniel Cruz, Viktor Mackert, Pascal Sous, Markus Sonnenberg, Hesham Solh, Lukas Pielsticker, Leander Kucklick, Walid Hetaba, Sebastian Daniel Hirt, Harry Ernst Hoster, Natalia Levin

**Affiliations:** ^1^ The Hydrogen and Fuel Cell Center (ZBT GmbH) Duisburg Germany; ^2^ Department of Inorganic Spectroscopy, Max‐Planck‐Institute for Chemical Energy Conversion Mülheim an der Ruhr Germany; ^3^ Department of Inorganic Chemistry Fritz‐Haber‐Institut der Max‐Planck‐Gesellschaft Berlin Germany; ^4^ Helmholtz‐Zentrum Berlin fürMaterialien und Energie Berlin Germany; ^5^ Energy Technology University of Duisburg‐Essen Duisburg Germany; ^6^ Center for Nanointegration Duisburg‐Essen (CENIDE) University of Duisburg‐Essen Duisburg Germany

**Keywords:** acidic stability, cation–anion co‐doping, manganese dioxide, oxygen evolution reaction, PEM water electrolysis

## Abstract

Developing acid‐stable, earth‐abundant oxygen evolution catalysts remains a central challenge for proton exchange membrane water electrolyzers (PEM‐WE), due to catalyst degradation via overoxidation under strongly anodic conditions. Here, we introduce a cation–anion co‐doping strategy that induces lattice‐level electronic stabilization in manganese dioxide (MnO_2_) through incorporation of niobium (Nb^5+^) and fluoride (F^−^) into a γ/β‐MnO_2_ framework. Structural and spectroscopic analyses, including operando X‐ray absorption spectroscopy, show that co‐doping stabilizes an electron‐enriched Mn environment and suppresses oxidation to unstable high‐valence states under OER conditions. This originates from charge redistribution across the Mn‐O‐Nb framework, enabling controlled Mn^3+^/Mn^4+^ dynamics and mitigating overoxidation‐driven dissolution. As a result, MnO_2_‐Nb‐F catalyst demonstrates an overpotential of 410 mV at 10 mA cm^−2^ in 0.5 M H_2_SO_4_, a Tafel slope of 90 mV dec^−1^, and reduced charge transfer resistance compared to pristine MnO_2_. When integrated into a PEM electrolyzer (5 cm^2^, 60 °C), the catalyst achieves a cell voltage of 1.91 V at 1 A cm^−2^ and exhibits operational stability for 380 h at 0.4 A cm^−2^. These results demonstrate that synergistic cation–anion engineering decouples the activity‐stability trade‐off in MnO_2_, establishing a viable design strategy for noble‐metal‐free PEM‐WE anodes.

## Graphical abstract

Stabilizing earth‐abundant catalysts for acidic oxygen evolution remains a major challenge for PEM water electrolysis. Here, Nb/F co‐modification electronically tunes MnO_2_ and stabilizes the Mn^3+^/Mn^4+^ redox equilibrium, suppressing overoxidation and structural degradation during acidic oxygen evolution, thereby enhancing the activity and durability of PGM‐free PEM electrolyzer anodes.

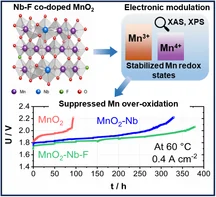

## 1 Introduction

Green hydrogen (H_2_) production via water electrolysis powered by renewable electricity is widely recognized as a cornerstone of industrial decarbonization [1, 2]. Among the different electrolysis technologies, proton exchange membrane water electrolysis (PEM‐WE) stands out for its ability to generate high‐purity H_2_ at high current densities (>1 A cm^−2^) while offering rapid dynamic response, making it ideally suited for integration with renewable energy sources and the utilization of surplus renewable electricity [3, 4, 5]. One of the main hurdles for PEM‐WE is the anode catalyst, due to the sluggish oxygen evolution reaction (OER) kinetics and its insufficient stability in the highly acidic working environment [6]. Presently, Ir‐based oxides are the only materials that combine sufficient activity and stability under these conditions, yet the extreme scarcity of Ir (<8 t year^−1^) and high cost (~$206 g^−1^) pose severe scalability constraints [7]. Meeting future H_2_ demand at the terawatt level would require several‐fold more Ir than is globally available, which is an unrealistic prospect [7, 8]. This fundamental bottleneck underscores the urgent need to design acid‐stable, platinum‐group‐metal (PGM)‐free OER catalysts that can sustain high activity and durability, thereby enabling the economic and large‐scale deployment of PEM electrolyzers. In view of this, various nonprecious OER materials have been reported in recent years, including MnSbO_*x*_ [9], MnCoO_*x*_ [10], Co_3_O_4_ [11], Mn_7.5_O_10_Br_3_ [12], and Ti_0.9_Nb_0.1_O_2−*x*_ [13]. Despite encouraging activity in acidic or near‐neutral electrolytes, most of these catalysts suffer from rapid dissolution and structural degradation at high anodic potentials [14]. More elaborate Co‐based and high‐entropy architectures have also been pursued to overcome this limitation, such as Ru‐Co_3_O_4_ (160 mV, 1500 h at 10 mA cm^−2^) [15], and Ir‐shell/high‐entropy‐core oxides (IrRuNiMoCo, 243 mV at 10 mA cm^−2^, >500 h at 1.0 A cm^−2^ in a full PEM‐WE) [16, 17] still rely on residual Ru or Ir content to reach these stability benchmarks. Consequently, achieving both activity and stability under strongly acidic conditions while fully excluding PGMs remains the key obstacle to replacing Ir‐based anodes.

In this context, among PGM‐free alternatives, Mn‐based oxides, particularly manganese dioxide (MnO_2_), are distinguished by its intrinsically low cost (2–3 $ kg^−1^), earth abundance, tunable electronic structure, defect density, and versatile Mn^2+^/Mn^3+^/Mn^4+^ redox chemistry [18]. This cost advantage holds even relative to other nonprecious candidates: MnO_2_ precursors remain substantially below cobalt (~25 $ kg^−1^) and orders of magnitude below PGMs such as Ir or Pt (>10^4^–10^5^ $ kg^−1^), underscoring the strong potential of Mn‐based catalysts for large‐scale electrolysis applications [8, 19]. Among different MnO_2_ polymorphs, γ‐MnO_2_ is especially appealing due to its mixed tunnel structures, which accommodate higher defect densities and provide accessible active sites that facilitate OER kinetics [20, 21, 22]. Benchmark studies have shown that γ‐MnO_2_ can sustain acidic OER operation for thousands of hours at low current densities (10 mA cm^−2^, 8000 h) in half‐cell measurements, while recent advances have enabled operation for ~1000 h at 200 mA cm^−2^ in full PEM‐WE cells [23, 24]. Despite these advantages, MnO_2_ remains unstable at anodic potentials above ~1.8 V vs. reversible hydrogen electrode (RHE), where overoxidation of Mn^4+^ species leads to the formation of soluble permanganate (MnO_4_^−^), resulting in irreversible catalyst dissolution and structural degradation [23]. To overcome these limitations, doping strategies have been employed to stabilize MnO_2_ by tuning its electronic structure, modulating the local Mn─O bonding environment, and suppressing excessive Mn oxidation under OER conditions [10, 12, 25, 26]. High‐valence cations such as Nb^5+^ have proven particularly effective for stabilizing transition‐metal oxides [13]. Nb incorporation can generate robust Nb─O bonds and compensating lattice defects, reinforcing the structural framework and increasing electron density around neighboring Mn sites [13, 26, 27]. This mitigates overoxidation and dissolution, consistent with the stabilization effects observed in Nb‐doped ruthenium oxides [27]. Computational and experimental studies on (Mn,Nb)O_2_ solid solutions further suggest that Nb incorporation modifies the Mn‐O electronic structure and metal–oxygen covalency, thereby tuning the adsorption energetics of OER intermediates and lowering the kinetic barriers for oxygen evolution [28]. In parallel, owing to its high electronegativity and ionic radius comparable to that of oxygen, F^−^ can partially substitute for O^2−^ within the MnO_2_ lattice, thereby inducing local electron redistribution, modifying the Mn─O bonding environment, and tuning the adsorption energetics of OER intermediates together with the Mn valence distribution [25, 29]. Consequently, fluoride incorporation has been reported to alter the electronic structure and interfacial reaction environment of transition‐metal oxides, which in several cases correlates with improved OER activity and enhanced electrochemical stability [28, 29, 30].

In this work, we combine spectroscopic and electrochemical analyses to understand the role of Nb/F doping in modulating the structure, activity, and stability of MnO_2_ under acidic OER conditions. X‐ray absorption near‐edge structure (XANES) and X‐ray photoelectron spectroscopy (XPS) measurements reveal how Nb^5+^ and F^−^ incorporation alter the local coordination and oxidation states, generating lattice distortion and defect oxygen environment that tunes the electronic structure. These modifications lead to enhanced OER activity and durability, which are further validated in PEM water electrolysis single‐cell tests.

## 2 Results and Discussion

**FIGURE 1:**
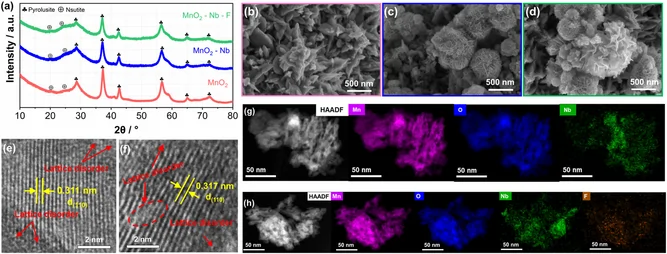
Structural and compositional characterizations. (a) XRD patterns. SEM images of (b) MnO_2_, (c) MnO_2_‐Nb, and (d) MnO_2_‐Nb‐F. HR‐TEM images of (e) MnO_2_‐Nb and (f) MnO_2_‐Nb‐F. HAADF–STEM with elemental mappings of Mn, O, Nb, and F for (g) MnO_2_‐Nb and (h) MnO_2_‐Nb‐F.

The MnO_2_ electrocatalysts were synthesized by anodic galvanostatic electrodeposition on Pt‐coated titanium (Ti) felt (350 µm), followed by thermal treatment (Figure S1a). Nb and F were incorporated into the MnO_2_ matrix using the same electrodeposition process as the undoped sample, followed by spray coating with aqueous NbCl_5_ or NbCl_5_ and NH_4_F precursor solutions and subsequent annealing (Figure S1b,c). To study the crystallinity and the phase purity of the three as‐prepared catalysts, powder X‐ray diffraction (P‐XRD) measurements were done, and the results are presented in Figure 1a. The pristine MnO_2_ exhibits distinct reflections at 2*θ* = 28.7°, 37.4°, 42.8°, 56.7°, 58.8°, and 64.9°, which correspond to the (110), (101), (111), (211), (220), and (002) planes of β‐MnO_2_ (pyrolusite) with tetragonal symmetry (space group P4_2_/mnm, JCPDS 24‐0735) [31]. These sharp and well‐defined reflections indicate a high degree of crystallinity and confirm the rutile‐type framework characterized by edge and corner‐sharing MnO_6_ octahedra [31, 32]. In addition to these dominant reflections, two features are observed at 20.3° and 24.4°, which are characteristic of the orthorhombic γ‐MnO_2_ phase (space group Pnam; nsutite, JCPDS 14‐0644), a defect structure exhibiting de Wolff disorder arising from intergrowths of ramsdellite and pyrolusite frameworks [20, 21]. The low‐intensity reflection at approximately 20.3° corresponds to lattice distortion resulting from irregular stacking of these domains, while the reflection at 24.4° can be indexed to the (110) plane of γ‐MnO_2_ (ICSD no. 78331). The presence of these reflections indicates that the electrodeposited MnO_2_ is predominantly pyrolusite type with intergrown γ‐MnO_2_ domains. Upon Nb doping (MnO_2_‐Nb), all key MnO_2_ reflections are preserved, and no additional reflections corresponding to secondary Nb‐containing phases (e.g., Nb_*x*_O_*y*_) are observed. Reflection broadening and minor negative shifts in major reflections, such as (101) from 2*θ* = 37.36° to 37.13°, (110) from 28.7° to 28.5°, and (211) from 56.76° to 56.5°, indicate structural perturbation and lattice expansion upon Nb incorporation. This can be rationalized by the substitution of Nb^5+^, which possesses a larger ionic radius (0.64 Å) than Mn^4+^ (0.53 Å) [33], within the MnO_6_ framework, inducing local lattice distortion and charge imbalance that are compensated through modified Mn coordination environments [26, 34]. These changes increase the interplanar spacing and introduce local strain, leading to reduced crystallite size and slightly enhanced disorder [35]. Fluoride co‐doping (MnO_2_‐Nb‐F) preserves the overall structure but induces subtle structural modifications. The diffraction pattern remains characteristic of γ/β‐MnO_2_, but subtle shifts, reflection broadening, and reduced peak intensities, especially around 37.4° and 56°, indicate increased strain, reduced crystallite size, and unit cell expansion. F^−^ ions likely substitute for lattice O^2−^ or occupy interstitial positions, thereby further modifying the local Mn‐O environment and promoting defect‐rich oxygen coordination together with partial Mn^4+^ to Mn^3+^ reduction [25, 30]. These effects reinforce the lattice expansion already induced by Nb doping. The co‐doped sample displays broader (28.7°), less intense reflections (37.4°, 42.8°, and 56.7°) indicative of increased structural disorder and defect density, yet it retains the original tunnel framework.

The morphologies of the prepared electrocatalysts were investigated using scanning electron microscopy (SEM). The pristine MnO_2_ exhibits a characteristic flake‐like morphology with densely stacked layers, typical of its tunnel‐structured framework (Figures 1b and S2a–c) [36]. Upon Nb incorporation, the morphology evolves into spherical, flower‐like assemblies with a roughened surface indicative of defect formation and structural distortion associated with Nb substitution (Figures 1c and S2d–f). Further introduction of F alongside Nb produces highly aggregated, porous architectures with pronounced hierarchical features, suggesting a synergistic effect of co‐doping on nucleation and growth processes (Figures 1d and S2g–i) [25, 37]. Such architectures not only expose a larger density of catalytic sites but also promote improved electrolyte penetration and mass transport [37]. The transition from flake‐like to hierarchical morphologies indicates that cation–anion co‐doping significantly influences the morphological evolution of MnO_2_. Transmission electron microscopy (TEM) images reveal that all three samples exhibit aggregated, irregular nanoparticulate clusters with slightly finer and more compact structures upon incorporation of Nb and F (Figures S3, S7, and S10). The high‐resolution TEM (HR‐TEM) image shows clear lattice fringes with interplanar spacings of 0.304 and 0.239 nm, corresponding to the (110) and (101) planes of pyrolusite MnO_2_, confirming its dominant crystalline phase (Figures S3d,e and S4). Mn substitution by Nb induces lattice strain within the pyrolusite structure, resulting in localized structural disorder (Figures S6 and S7). The cointroduction of F further amplifies the lattice distortions, resulting in a more heterogeneous and defect‐rich structure (Figures S9 and S10). Such coupled cation–anion doping induces substantial lattice strain and local disorder, which can modulate the electronic structure, enhance charge transfer, and increase the density of electrochemically active sites [38]. HR‐TEM images of MnO_2_‐Nb and MnO_2_‐Nb‐F (Figure 1e,f) reveal lattice fringes with interplanar spacings of 0.311 and 0.317 nm, respectively, corresponding to the (110) planes of pyrolusite‐type MnO_2_. The slight lattice expansion in the doped samples is consistent with Nb^5+^ incorporation into the MnO_2_ framework and is accompanied by reduced long‐range crystallinity [39]. In addition, the partial substitution of O^2−^ by F^−^ may induce local bond relaxation and generate defect‐related low‐coordination/hydroxylated oxygen environments, further contributing to lattice disorder while preserving the overall pyrolusite structure [40]. Particle‐size distribution analysis from TEM images shows a reduction in particle size upon the addition of two dopants, with the average size decreasing from 15.1 ± 1.3 nm for MnO_2_ to 12.5 ± 3 nm for MnO_2_‐Nb and 11.3 ± 1.8 nm for MnO_2_‐Nb‐F, respectively (Figures S3, S7, and S10). High‐angle annular dark‐field scanning TEM (HAADF–STEM), combined with elemental mapping at both low and high magnifications, reveals a homogeneous distribution of Mn and O across the MnO_2_ (Figure S5) and of Mn, O, Nb, and F across the MnO_2_‐Nb (Figures 1g and S8) and MnO_2_‐Nb‐F samples (Figures 1h and S11), confirming uniform dopant incorporation at the nanoscale. TEM‐EDX elemental mapping confirms fluorine incorporation in the MnO_2_‐Nb‐F sample, with signals observed in localized nanoscale regions of the catalyst. Quantitative energy‐dispersive X‐ray spectroscopy (EDX) analysis provides further evidence of successful dopant incorporation across the series (Table S1). Pristine MnO_2_ exhibits a near‐stoichiometric composition of 28.9 ± 0.3 at.% Mn and 71.1 ± 1.7 at.% O. Upon Nb substitution, the MnO_2_‐Nb sample shows a bulk Nb content of 7.2 ± 1.8 at.%, with Mn and O concentrations of 26.3 ± 4.5 and 66.5 ± 2.2 at.%, respectively. For the MnO_2_‐Nb‐F co‐doped catalyst, the presence of both dopants is confirmed with a bulk composition of 5.8 ± 1.3 at.% Nb and 3.5 ± 1.8 at.% F, while the Mn and O contents are measured at 25.4 ± 2.1 and 65.3 ± 4.8 at.%, respectively. Selected‐area electron diffraction (SAED) patterns (Figure S12) further show a gradual reduction in crystallinity upon Nb and Nb/F modification, as evidenced by progressive broadening and decreased intensity of diffraction rings. Nevertheless, all samples retain the characteristic diffraction features of pyrolusite‐type MnO_2_ phase, confirming retention of the crystal structure and the absence of secondary oxide phases or other crystalline impurities, which are consistent with the XRD data.

To further understand the textural properties of the catalysts, Brunauer–Emmett–Teller (BET) analysis was conducted using N_2_ adsorption–desorption isotherms (Figure S13) to evaluate their specific surface area (SSA) and porosity. The isotherms of MnO_2_, MnO_2_‐Nb, and MnO_2_‐Nb‐F exhibit typical type IV isotherms with H_3_‐type hysteresis loops, characteristic of mesoporous materials. All three samples display a sharp increase in adsorbed volume at high relative pressures (*p*/*p*_0_ > 0.8), indicating capillary condensation within mesopores. Compared to pristine MnO_2_, the Nb‐ and Nb‐F‐doped samples show a notable enhancement in N_2_ uptake, particularly in the medium‐ to high‐pressure region, suggesting the development of a more open and accessible pore structure. Pristine MnO_2_ exhibits a BET SSA of 56.7 m^2^ g^−1^, which increases upon Nb incorporation to 61.3 m^2^ g^−1^, aligning with previous reports [28, 33]. Interestingly, fluorination results in a moderate decrease in the SSA to 59.4 m^2^ g^−1^, likely due to partial particle agglomeration associated with the exothermic decomposition of the fluorine precursor [41]. Despite the reduced SSA, F incorporation modifies the pore architecture, which may contribute to improved mass transport characteristics. The MnO_2_‐Nb‐F sample exhibits the highest pore volume (0.235 cm^3^ g^−1^), derived from N_2_ adsorption at high relative pressure, and a large average pore diameter (11.3 nm), estimated from Barret–Joyner–Halenda (BJH) analysis [42], reflecting an open and accessible mesoporous network. In comparison, MnO_2_‐Nb shows moderate mesoporosity with an average pore diameter of 8.7 nm and a high SSA, offering a balance between active‐site density and ion diffusion. This trend is corroborated by the aggregated, hierarchical morphology observed by SEM for MnO_2_‐Nb‐F (Figures 1d and S10) and by TEM particle‐size analysis, which shows the average primary crystallite size decreasing across the series. Smaller crystallites have higher surface energy per unit mass, making them more prone to coalescing into larger secondary particles during thermal treatment [43], consistent with the decreased SSA alongside increased pore volume and pore diameter and inconsistent with pore blockage or network collapse.

**FIGURE 2:**
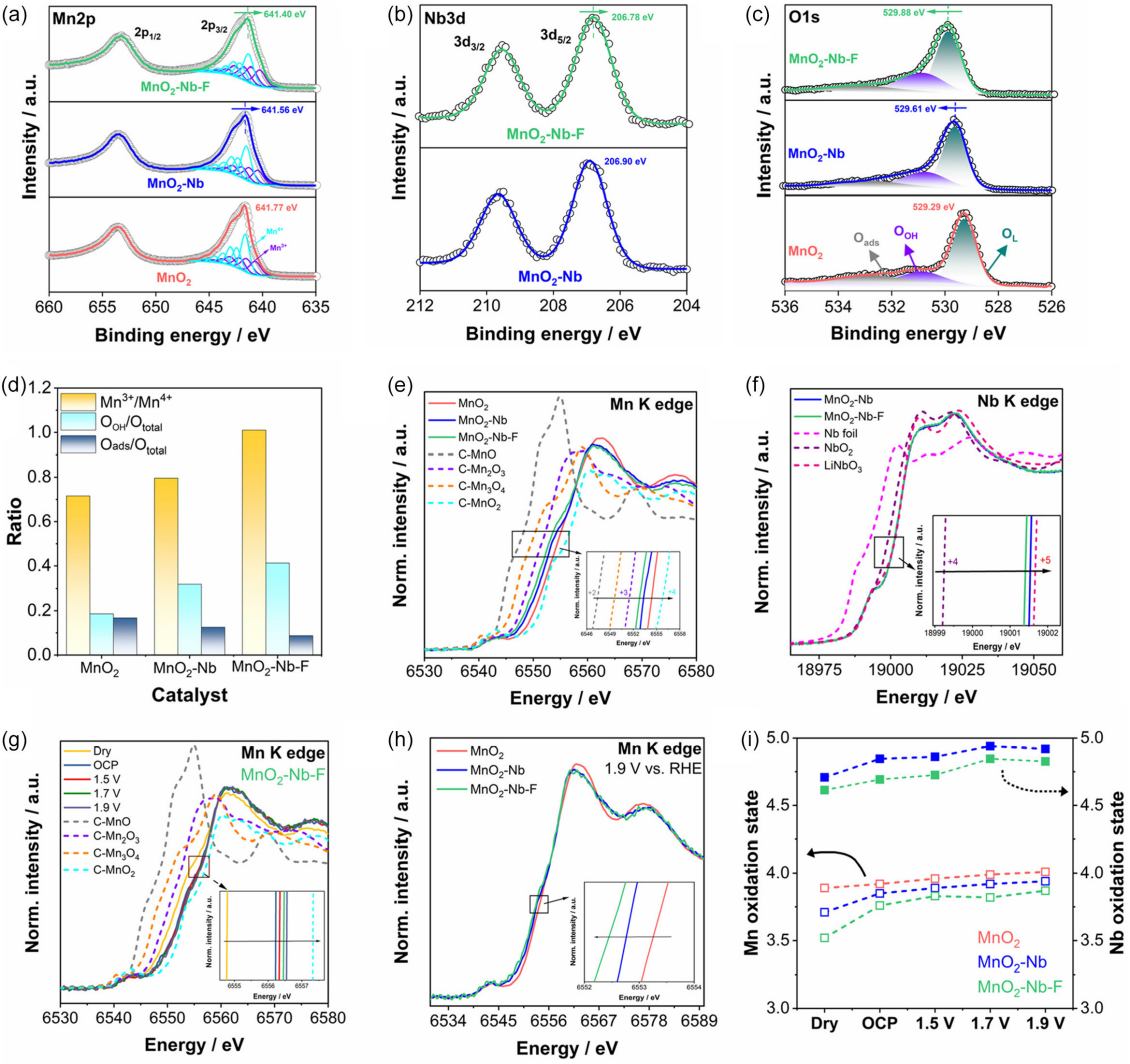
XPS and XAS characterizations of the three electrocatalysts. (a) Mn 2p XPS spectra, (b) Nb 3d XPS spectra, and (c) O 1s XPS spectra of MnO_2_, MnO_2_‐Nb, and MnO_2_‐Nb‐F. (d) Quantitative ratios derived from XPS fitting. (e) Mn K‐edge XANES spectra of MnO_2_, MnO_2_‐Nb, and MnO_2_‐Nb‐F compared with Mn references. (f) Nb K‐edge XANES spectra of MnO_2_‐Nb and MnO_2_‐Nb‐F referenced with Nb references. (g) Operando Mn K‐edge XANES spectra of MnO_2_‐Nb‐F collected under dry, electrolyte‐contacted (no applied bias), and anodic polarization conditions. (h) Mn K‐edge at 1.9 V vs. RHE highlights edge shifts among MnO_2_, MnO_2_‐Nb, and MnO_2_‐Nb‐F. (i) Evolution of Mn and Nb oxidation states as a function of condition, derived from K‐edge absorption energies using reference‐based calibration.

The surface electronic structure and chemical environment of the MnO_2_‐based catalysts were investigated by XPS, which provides insight into the influence of Nb and F co‐doping on the lattice coordination, oxidation states, and surface defect chemistry (Figure 2a–d). Peak fitting of the Mn 2p region was performed using literature‐reported multiplet splitting parameters for Mn species in transition‐metal oxides [44]. The XPS survey spectra (Figure S14) confirm successful Nb incorporation in MnO_2_‐Nb and MnO_2_‐Nb‐F through the appearance of Nb 3d signals, while all samples show characteristic Mn 2p and O 1s peaks with no additional impurity‐related features. Pristine MnO_2_ displays Mn 2p_3/2_ and Mn 2p_1/2_ peaks at 641.77 and 653.65 eV, respectively, with a spin–orbit splitting of 11.88 eV, consistent with Mn^4+^ in octahedral [MnO_6_] coordination [25, 29]. Upon Nb and Nb/F incorporation, both peaks shift negatively by 0.23 and 0.39 eV, respectively, reflecting an increase in the electronic density around Mn, confirming the partial reduction of Mn^4+^ to Mn^3+^. This shift indicates a redistribution of electron density within the Mn‐O framework, leading to local lattice polarization and the generation of electronically perturbed Mn sites [45]. These modified coordination environments can facilitate oxygen mobility and promote the formation of defect‐rich oxygen environments, which may provide additional adsorption and reaction sites during the OER [46]. Quantitative fitting confirms a progressive increase in the Mn^3+^/Mn^4+^ ratio from 0.69 (MnO_2_) to 0.81 (MnO_2_‐Nb) and 1.03 (MnO_2_‐Nb‐F), indicating partial reduction of Mn^4+^ through charge compensation triggered by Nb^5+^ and F^−^ substitution (Figure 2d). The higher Mn^3+^ content, known to be catalytically active in Mn‐based oxides, enhances the redox flexibility and intrinsic electronic conductivity of the material. Quantitative XPS analysis reveals the near‐surface elemental composition of the electrocatalysts (Table S2). Pristine MnO_2_ shows 20.6  at.% Mn and 59.7 at.% O, consistent with the pyrolusite stoichiometry. Upon Nb incorporation, MnO_2_‐Nb exhibits a surface Nb content of 9.7 at.%, together with 14.9 at.% Mn and 57.4 at.% O, confirming effective Nb incorporation. For the MnO_2_‐Nb‐F catalyst, the surface composition comprises 12.3 at.% Mn, 54.1 at.% O, and 6.9 at.% Nb. The Nb 3d spectrum (Figure 2b) exhibits two well‐defined peaks at 206.90 eV (3d_5/2_) and 209.67 eV (3d_3/2_), with a spin–orbit splitting of 2.77 eV. This binding energy lies much closer to reported Nb_2_O_5_ values (207.20 eV) than to NbO_2_ (205.20 eV) [47], and no lower binding energy shoulder or additional component is observed near 205–206 eV, consistent with a single Nb^5+^ oxidation state. The small offset from the Nb_2_O_5_ reference value (~0.3 eV) is attributed to Nb^5+^ incorporation into the MnO_2_ lattice, together with differences in final‐state screening and the applied charge correction procedure, rather than to a reduced Nb species. Following fluoride incorporation, a negative binding energy shift of 0.12 eV is observed together with a concomitant shift of the Mn 2p peaks, indicating electron redistribution and modification of the local electronic environment after Nb/F incorporation. To further confirm this assignment, depth‐resolved synchrotron XPS was performed on MnO_2_‐Nb‐F (Figure S15). A single Nb^5+^ 3d doublet was resolved at every probed depth (3*λ* ≈ 2–7 nm), with no metallic or segregated oxide shoulder near 205 eV, confirming genuine lattice incorporation rather than a surface‐adhered species. MnO_2_‐Nb shows the same binding energy and line shape by laboratory XPS, consistent with the same Nb^5+^ assignment. The same depth‐resolved dataset provides direct evidence for the chemical state of fluorine, which fell below the detection limit of laboratory (Al Kα) XPS due to low fluorine coverage, modest F 1s cross‐section at Al Kα excitation, and the strong surface weighting of Al Kα toward the outermost, most fluorine‐poor 1–2 nm (Figure S16). The F 1s core level resolves into two chemical states at every probed depth (3*λ* ≈ 2–7 nm): a covalent F–O/F–OH component at 687.3 eV, concentrated near the surface, and an ionic metal‐fluoride (Mn/Nb‐F) component at 684.2 eV, whose fraction of the total F 1s signal rises with depth (~13% to ~21%). This ionic state reflects fluorine substituting oxygen within the metal–oxide lattice, distinct from surface‐bound fluoride, and its depth dependence shows lattice‐substituted F is not confined to the surface. Rather than inferring depth distribution from surface‐weighted XPS‐versus‐EDX at.% comparisons, a sensitivity‐normalized depth‐contrast factor R (bulk‐ to surface‐sensitive intensity ratio; *R* > 1 = subsurface‐enriched) was defined from the same dataset (Figure S20). Nb (*R* ≈ 10.2), ionic Mn/Nb‐F (*R* ≈ 1.9), and the Mn^3+^/Mn^4+^ ratio (*R* ≈ 9.4) are all subsurface‐enriched, opposite to what a surface‐adhered NbO_*x*_ or Nb/F film would show. This indicates a thin (~1–2.5 nm) hydroxylated outer skin over an Nb(V)‐ and lattice‐F‐enriched interior (Figure S22), consistent with genuine substitutional incorporation rather than surface segregation. The O 1s spectra provide complementary evidence of the defects induced by Nb and F (Figure 2c). All samples show three main components: lattice oxygen (*O*_L_—529.29 eV), defect‐associated hydroxyl species (*O*_OH_—530.95 eV), and adsorbed oxygen species (*O*_ads_—532.87 eV [26, 44, 48]. Compared with pristine MnO_2_, Nb incorporation increases the *O*_OH_ contribution, indicating the generation of defect‐rich hydroxylated oxygen environments, while additional F incorporation further enhances this trend accompanied by a slight decrease in the *O*_L_ and *O*_ads_ fractions. In addition, the lattice O 1s peak shifts toward higher binding energies by 0.32 and 0.59 eV for MnO_2_‐Nb and MnO_2_‐Nb‐F, respectively, indicating enhanced Mn─O bond polarization and modification of the local electronic structure upon Nb and F incorporation [10, 26, 27]. Quantitative analysis (Figure 2d) further supports these trends: The *O*_OH_/*O*_total_ ratio increases from 0.18 for MnO_2_ to 0.31 for MnO_2_‐Nb and 0.42 for MnO_2_‐Nb‐F, whereas the *O*_ads_/*O*_total_ ratio decreases from 0.16 to 0.11 and 0.08, respectively, consistent with progressive modification of the Mn‐O electronic environment.

To study the electronic structure modulation induced by Nb and F incorporation, operando XAS was conducted. The setup of the operando experiments and detailed experimental procedures are provided in the Supporting Information, and schematics of the operando cell and the setup are shown in Figures S25 and S26. The normalized Mn K‐edge XANES spectra of dry MnO_2_, MnO_2_‐Nb, and MnO_2_‐Nb‐F (Figure 2e) exhibit similar overall spectral profiles, indicating that the local Mn coordination environment remains predominantly octahedral across all compositions. Nevertheless, distinct systematic shifts in the absorption edge position are observed, reflecting subtle yet significant modifications in the Mn electronic structure. The Mn K‐edge positions indicate that the average Mn oxidation state lies between +3 and +4 for all samples (Figure 2e). Compared to pristine (dry) MnO_2_, the Mn K‐edge of MnO_2_‐Nb shifts toward lower energy, indicating partial reduction of Mn upon Nb incorporation. This negative shift becomes more pronounced after F doping (MnO_2_‐Nb‐F), suggesting enhanced electronic localization and modified Mn─O covalency at the Mn centers, also consistent with the surface analysis (Figure 2e). The oxidation states were quantified using the absorption edge energy (*E*_0_), determined from the maximum of the first derivative, and calibrated against MnO, Mn_3_O_4_, Mn_2_O_3_, and MnO_2_ standards (Figure S29e). Quantitative analysis of the Mn K‐edge reveals a clear and systematic evolution of the oxidation state with composition and applied potential. At dry conditions, the average Mn oxidation state decreases from 3.89 in MnO_2_ to 3.71 in MnO_2_‐Nb and further to 3.52 in MnO_2_‐Nb‐F, confirming that Nb incorporation and subsequent F doping progressively stabilize Mn in a more reduced state. Upon electrolyte circulation, all samples exhibit an increase in Mn valence, indicating partial anodic activation of the surface. Under OER‐relevant potentials, the Mn oxidation state increases continuously from OCP to 1.9 V vs. RHE. For pristine MnO_2_, the oxidation state rises from 3.92 (OCP) to 4.01 (1.9 V), reflecting significant oxidation toward Mn^4+^‐like species (Figures 2i and S19a). In contrast, MnO_2_‐Nb and MnO_2_‐Nb‐F display a much more moderate increase, reaching 3.94 and 3.87 at 1.9 V, respectively (Figures 2i and S19b). Notably, MnO_2_‐Nb‐F maintains the lowest Mn valence across the entire potential range, demonstrating enhanced resistance to excessive anodic oxidation. The suppressed oxidation response at high potentials suggests that Nb and F incorporation induce lattice‐level charge redistribution within the Mn‐O‐Nb framework, modifying Mn─O covalency and stabilizing Mn against excessive oxidation and structural degradation under OER conditions.

The Nb K‐edge XANES spectra provide further insight into the lattice electronic structure evolution (Figure 2f). The absorption energies of MnO_2_‐Nb and MnO_2_‐Nb‐F are positioned between NbO_2_ (+4) and LiNbO_3_ (+5) references, confirming that Nb predominantly exists in a high oxidation state within the lattice (Figure S29f). This assignment is independently supported by Nb 3d spectrum XPS, which indicates Nb is predominantly present in an oxidation state close to +5 (Figure 2b). Notably, a very small but reproducible negative edge shift is observed upon F doping, indicating slight reduction of Nb. Moreover, comparison at 1.9 V vs. RHE shows that Nb in MnO_2_‐Nb‐F remains in a relatively reduced oxidation state even at the highest applied potential (Figure 2i). The observed negative shift signifies increased electronic screening and enhanced charge density around Nb sites, consistent with lattice‐wide electronic redistribution induced by F incorporation. Similarly, Nb K‐edge analysis shows that Nb remains in a high oxidation state under all conditions but undergoes subtle electronic modulation upon F incorporation. At dry state, Nb exhibits a slightly more oxidized state for MnO_2_‐Nb (Figure S29c) than for MnO_2_‐Nb‐F (Figure S29d). This trend is maintained under OER conditions, where MnO_2_‐Nb‐F remains with lower valence. The uniform reduction across the potential range confirms that F incorporation drives lattice‐level charge redistribution, creating a more reduced electronic environment around Nb, while the smaller potential‐dependent change compared to Mn indicates that Nb functions mainly as an electronic modulator rather than an active redox center.

**FIGURE 3:**
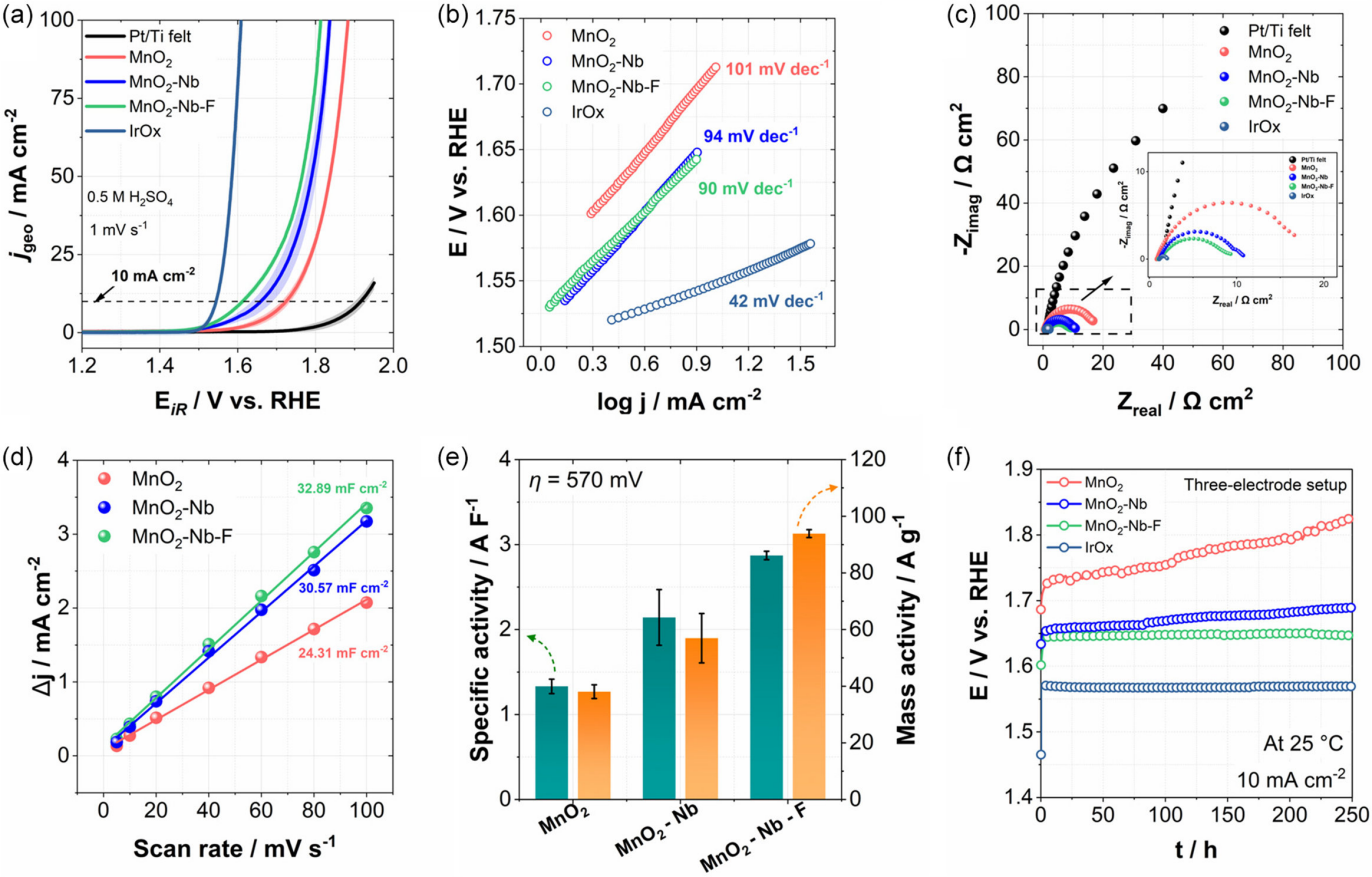
Electrochemical characterization of the three electrocatalysts. (a) Averaged LSV curves recorded at 1 mV s^−1^ and compared with IrO*_x_* and bare Pt/Ti felt in 0.5 M H_2_SO_4_. The dashed areas represent the standard deviation obtained from three repeated measurements. (b) Tafel plots derived from LSV data, (c) Nyquist plots at 1.75 V vs. RHE, (d) double‐layer capacitance, (e) specific and mass activities at an overpotential of 570 mV, and (f) chronopotentiometry of MnO_2_‐based anodes and IrO_*x*_ reference at 10 mA cm^−2^.

The electrocatalytic performance of the synthesized catalysts toward the OER was evaluated under acidic conditions using linear sweep voltammetry (LSV) and electrochemical impedance spectroscopy (EIS), as shown in Figure 3. The LSV curves (Figure 3a) reveal that incorporating MnO_2_ with Nb and F significantly enhances its OER activity. At a current density of 10 mA cm^−2^, the MnO_2_‐Nb‐F catalyst exhibits the lowest overpotential of 0.410 ± 0.004 V, compared to 0.429 ± 0.013 V for MnO_2_‐Nb and 0.506 ± 0.006 V for pristine MnO_2_. At a higher current density of 100 mA cm^−2^, the corresponding overpotentials increase to 0.581 ± 0.004, 0.606 ± 0.013, and 0.664 ± 0.006 V, respectively (Figure S27). The significant reduction in overpotential for the MnO_2_‐Nb‐F and MnO_2_‐Nb catalysts across both kinetic and high‐current regions emphasizes the beneficial effect of Nb and F co‐doping on enhancing the oxygen evolution kinetics in acidic conditions. Tafel plots derived from the LSV data in the kinetic region (Figure 3b) reveal that MnO_2_‐Nb‐F exhibits a lower slope (90 mV dec^−1^) compared to MnO_2_ (101 mV dec^−1^) and MnO_2_‐Nb (94 mV dec^−1^), indicating improved reaction kinetics upon co‐doping. For reference, IrO_*x*_ shows the lowest slope (42 mV dec^−1^), consistent with its well‐established high OER activity. To investigate the electrochemical reaction kinetics and the charge transfer resistance (*R*_ct_) at the electrocatalyst/electrolyte interface, EIS analysis was carried out at 1.75 V (vs. RHE) in the electrolyte solution of 0.5 M H_2_SO_4_. The Nyquist plots (Figure 3c) show that besides the IrO_*x*_ benchmark material, the MnO_2_‐Nb‐F electrode achieves the lowest charge transfer resistance (*R*_ct_ ≈ 8.28 Ω cm^2^), indicating more efficient interfacial charge transfer during the OER. The MnO_2_‐Nb electrode exhibits a moderate *R*_ct_ of 9.65 Ω cm^2^, while pristine MnO_2_ displays the highest *R*_ct_ of 15.79 Ω cm^2^, consistent with sluggish charge transfer kinetics. These results confirm that Nb and F co‐doping substantially reduces the charge transfer resistance of MnO_2_ and enhances its intrinsic catalytic activity.

The influence of the electrochemically active surface area (ECSA) on the OER activity was evaluated by determining the double‐layer capacitance (*C*_dl_). Cyclic voltammetry (CV) was performed in the non‐Faradaic region at various scan rates (5–100 mV s^−1^), and the difference in current density (Δ*j*) between anodic and cathodic sweeps at a fixed potential was plotted against the scan rate (Figures 3d and S29). The linear fits yield *C*_dl_ values of 24.31 mF cm^−2^ for MnO_2_, 30.57 mF cm^−2^ for MnO_2_‐Nb, and 32.89 mF cm^−2^ for MnO_2_‐Nb‐F. Because *C*_dl_ correlates with the ECSA [49], the higher capacitance values observed for the Nb‐ and Nb‐F‐doped catalysts suggest increased exposure of electrochemically accessible active sites. This correlates well with the improved OER activity observed for the doped catalysts. To further understand the origin of the observed activity enhancement, the specific and mass activities were evaluated at an overpotential of 570 mV (Figure 3e). The specific activity increases from 1.33 A F^−1^ for MnO_2_ to 2.14 A F^−1^ for MnO_2_‐Nb and further to 2.87 A F^−1^ for MnO_2_‐Nb‐F. A similar trend is observed for mass activity which increases from 38.1 A g^−1^ (MnO_2_) to 57.0 A g^−1^ (MnO_2_‐Nb) and 93.9 A g^−1^ (MnO_2_‐Nb‐F). To separate the individual and synergistic contributions of Nb and F doping, a MnO_2_‐F control sample was prepared and evaluated under identical conditions. MnO_2_‐Nb‐F consistently outperformed pristine MnO_2_ and both singly doped controls across all electrochemical metrics, including overpotential, Tafel slope, and specific and mass activities (Figures S27, S28a–d, and S30). Kinetically, MnO_2_‐F reaches an overpotential of 0.450 ± 0.002 V at 10 mA cm^−2^, closely matching MnO_2_‐Nb (0.429 ± 0.013 V) in this low‐current regime. Quite interestingly, however, at higher current density, this advantage fades: At 100 mA cm^−2^, MnO_2_‐F requires 0.647 ± 0.006 V, converging toward the value for undoped MnO_2_ (0.664 ± 0.002 V), indicating that F incorporation alone is not sufficient to sustain the activity enhancement into the mass transport–limited regime. These results support the conclusion that the enhanced OER activity arises from a synergistic effect of Nb and F co‐doping rather than from either dopant alone, reflecting a significant intrinsic kinetic enhancement beyond surface area effects.

To assess long‐term stability, chronopotentiometry (CP) was performed for MnO_2_, MnO_2_‐Nb, MnO_2_‐Nb‐F, and the IrO_*x*_ benchmark at a constant current density of 10 mA cm^−2^ in 0.5 M H_2_SO_4_ for 250 h (Figure 3f). The IrO_*x*_ benchmark maintains an essentially constant potential of  ~1.57 V vs. RHE throughout the test, consistent with its established stability under acidic OER conditions. In contrast, pristine MnO_2_ shows the most pronounced degradation, with the potential rising sharply from 1.685 V at *t* = 0 to approximately 1.73 V within the first ~10 h, followed by a continued gradual increase to 1.82 V by 250 h. MnO_2_‐Nb shows substantially improved stability, rising from an initial 1.63 V to approximately 1.65 V within the first ~10 h and reaching 1.69 V after 250 h, with a discernible acceleration in the later stage of the test (beyond ~150 h). MnO_2_‐Nb‐F exhibits the most stable behavior among the doped catalysts, stabilizing at 1.645 V within the first few hours and increasing only marginally to 1.650 V over the full 250 h, corresponding to a decay rate of 0.020 mV h^−1^, indicative of a stable performance among the nonprecious metal catalysts under acidic OER conditions. To probe the origin of the observed degradation, the electrolyte was analyzed by ICP‐OES after 250 h of chronopotentiometric testing at 10 mA cm^−2^. The measured Mn concentrations were 2372.3 µg/L for pristine MnO_2_, 320.80 µg/L for MnO_2_‐Nb, and 132.52 µg/L for MnO_2_‐Nb‐F, an ~18‐fold reduction for the co‐doped catalyst relative to pristine MnO_2_. Notably, Nb was below the detection limit of the instrument in all samples, indicating negligible Nb dissolution and confirming its stability in the acidic electrolyte. This shows that the observed degradation is governed by Mn leaching rather than Nb dissolution and that this leaching is substantially suppressed by Nb doping and further diminished by Nb‐F co‐doping. We compared the OER performance of MnO_2_‐Nb‐F with reported non‐PGM electrocatalysts in terms of overpotential, mass activity, and stability. As shown in Table S3, MnO_2_‐Nb‐F is among the most promising non‐PGM OER electrocatalysts reported, combining a competitive overpotential at 10 mA cm^−2^ with a leading mass activity (93.9 A g^−1^ at 570 mV) and negligible degradation over 250 h.

**FIGURE 4:**
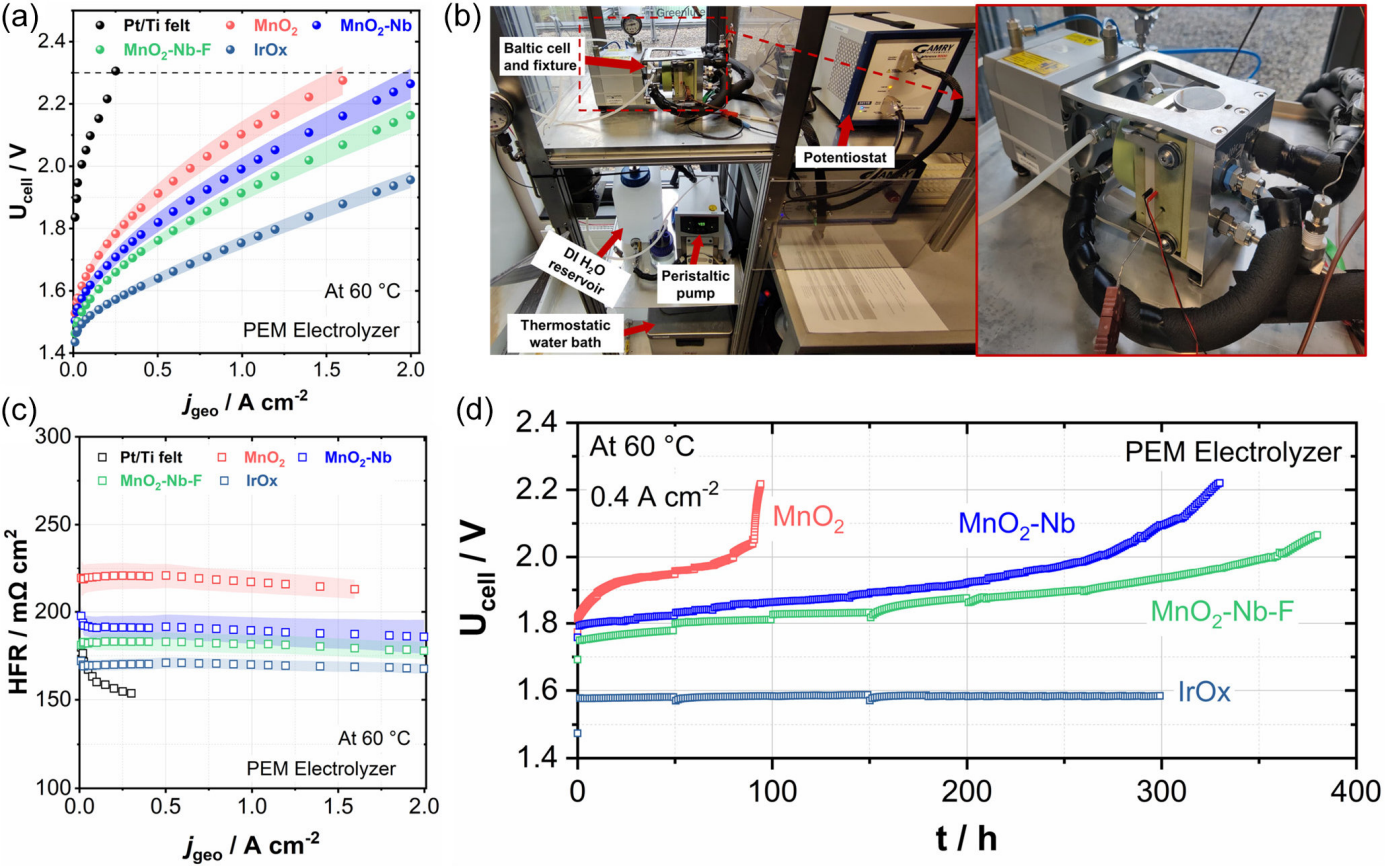
Performance of the electrocatalyst for PEM electrolysis at 60 °C. (a) Steady‐state polarization curves for MnO_2_‐based anodes, IrO_*x*_, and bare Pt/Ti felt. (b) Experimental setup used for electrochemical testing in the Baltic cell. (c) High‐frequency resistance as a function of current density and (d) comparison of the long‐term durability of MnO_2_‐based anodes and IrO_*x*_ reference. The dashed areas represent the standard deviation obtained from three repeated measurements.

Based on the improved three‐electrode performance of MnO_2_‐Nb‐F, its activity and stability were further assessed under PEM electrolyzer conditions by applying 20 wt% Pt/C as cathode catalyst. The electrochemical measurement setup is illustrated in Figure 4b. A membrane electrode assembly (MEA) was fabricated using a hybrid configuration. For the cathode, a 20 wt% Pt/C catalyst ink was sprayed directly onto a pretreated Nafion 115 membrane (active area: 5 cm^2^) following the catalyst‐coated membrane (CCM) approach (Figure S31). The final MEA was formed by hot‐pressing the catalyst‐coated PTL against the cathode‐coated membrane (Figure S32). The electrochemical performance and high‐frequency resistance (HFR) characteristics of the MnO_2_‐based catalysts are presented in Figure 4a,c. Prior to HFR correction (Figure 4a), all electrodes exhibit an expected exponential rise in cell voltage with increasing current density, characteristic of kinetically controlled oxygen evolution followed by resistive and mass transport limitations at higher currents. Among the tested catalysts, MnO_2_‐Nb‐F consistently delivers the lowest cell voltage across the entire range, while pristine MnO_2_ displays the highest cell voltages. The MnO_2_‐Nb sample exhibits intermediate performance, indicating that Nb incorporation already improves the catalytic properties of MnO_2_. The further enhancement observed for MnO_2_‐Nb‐F can be attributed to improved electronic pathways and reduced interfacial resistance, as evidenced by the lower HFR and *R*_ct_ values. In addition, the gradual decrease in Tafel slope suggests a modification of the reaction kinetics, likely arising from electronic structure changes induced by Nb and F incorporation in the Mn‐O framework. In the low‐current activation region (*j* < 0.2 A cm^−2^), pristine MnO_2_ exhibits a sluggish voltage rise, indicative of higher activation barriers, whereas Nb and F incorporation markedly lowers the cell voltage (1.58 V for MnO_2_‐Nb‐F and 1.65 V for MnO_2_‐Nb vs. 1.72 V for MnO_2_). At higher current densities, this trend becomes more pronounced: The MnO_2_‐Nb‐F electrode achieves 1 A cm^−2^ at 1.91 V, while MnO_2_‐Nb requires 1.98 V, and pristine MnO_2_ reaches only 1.5 A cm^−2^ even at 2.3 V, revealing substantial resistive and mass transport limitations (Figure 4a). The corresponding HFR profiles (Figure 4c) further support this behavior, showing a progressive reduction from 220 mΩ cm^2^ for MnO_2_ to 192 mΩ cm^2^ for MnO_2_‐Nb and 174 mΩ cm^2^ for MnO_2_‐Nb‐F, indicating reduced Ohmic losses and improved electronic pathways within the catalyst layer.

The electrochemical durability of the MnO_2_‐Nb‐F anode was evaluated at a constant current density of 0.4 A cm^−2^ and 60 °C under PEM electrolyzer operating conditions and compared with pristine MnO_2_ (Figure 4d). These measurements were performed under device‐relevant conditions, where catalyst stability, membrane hydration, and interfacial charge and mass transport collectively influence long‐term electrolyzer performance. The MnO_2_‐Nb‐F cell exhibits the lowest beginning‐of‐life (BOL) voltage (1.74 V), followed by MnO_2_‐Nb (1.78 V), while pristine MnO_2_ starts at a higher voltage of 1.80 V, reflecting inferior intrinsic catalytic activity. During the initial 100 h, MnO_2_‐Nb‐F shows a gradual voltage increase to 1.81 V, corresponding to a very low degradation rate of 0.71 mV h^−1^. In comparison, MnO_2_‐Nb displays a moderately higher voltage rise from 1.78 to 1.86 V over the same period (~0.8 mV h^−1^), indicating partial stabilization but still inferior to the co‐doped system. In contrast, pristine MnO_2_ degrades significantly faster, reaching 1.93 V within 50 h, corresponding to a decay rate of 2.6 mV h^−1^. At extended operation times, MnO_2_‐Nb‐F transitions into a stabilized regime, with the voltage increasing more slowly to 1.87 V at 200 h and 1.90 V at 270 h (0.55 mV h^−1^), confirming sustained kinetic stability. MnO_2_‐Nb, however, exhibits a clear deviation from this behavior beyond 200 h, where the voltage increases more rapidly, reaching 2.01 V at 270 h, indicating the onset of accelerated degradation. In contrast, pristine MnO_2_ continues its rapid deterioration, reaching 1.98 V at 70 h and 2.02 V at 85 h, followed by a pronounced failure event where the voltage sharply rises to 2.17 V at 95 h, signifying severe catalyst degradation and loss of functionality. At longer timescales, MnO_2_‐Nb also enters a regime of accelerated decay, with the voltage rising steeply beyond 270–300 h and approaching 2.17 V, although this transition occurs significantly later than for pristine MnO_2_. In contrast, MnO_2_‐Nb‐F maintains stable electrolysis over substantially extended durations, with a delayed transition only after 300 h, where the voltage increases from 1.91 to 2.06 V at 380 h, corresponding to a higher late‐stage degradation rate of 1.87 mV h^−1^.

Direct ICP‐OES quantification of Mn/Nb dissolution was not performed in the single‐cell configuration, as the recirculating water exits the cell as product effluent and is continuously routed through a mixed‐bed ion‐exchange deionizer (Figure S33) before returning as feed, which would scavenge dissolved cations and render downstream effluent measurements unrepresentative of the true catalyst‐surface dissolution rate. As a complementary, operando probe of interfacial degradation mechanisms, galvanostatic electrochemical impedance spectroscopy (GEIS) was employed to further examine the origin of the observed durability trends (Figures S34–S36). The Nyquist spectra are characterized by a single semicircular feature associated with interfacial charge transfer at the catalyst/electrolyte interface. For MnO_2_‐Nb‐F, the evolution of this feature remains limited, with only a moderate increase in semicircle diameter over time, corresponding to a gradual rise in *R*_ct_ from 283 to 466 mΩ cm^2^. In contrast, the Ohmic resistance exhibits a slight initial decrease (190 mΩ cm^2^ at 0 h), which can be attributed to membrane rehydration and improved interfacial contact, and remains largely unchanged thereafter. This behavior indicates that performance degradation is dominated by kinetic rather than Ohmic contributions. A distinctly different trend is observed for MnO_2_‐Nb, where the semicircle expands more significantly during operation, with *R*_ct_ increasing from ~324 to 708 mΩ cm^2^. This progressive increase in interfacial impedance reflects a continuous decline in charge transfer kinetics, suggesting partial instability of the active surface under OER conditions. This effect is markedly amplified in pristine MnO_2_, where the Nyquist semicircle undergoes a severe expansion, with *R*_ct_ rising from ~760 mΩ cm^2^ at 0 h to 5509 mΩ cm^2^ at 95 h. The correlation between the increase in *R*_ct_ and the observed voltage profile during durability testing indicates that performance losses are dominated by kinetic limitations rather than Ohmic contributions. Mechanistically, this behavior is attributed to dissolution of Mn‐based active sites under strong oxidative OER conditions, consistent with the Mn leaching data and *R*_ct_ evolution presented above. While Nb incorporation partially suppresses these processes, the combined Nb and F co‐doping more effectively stabilizes the catalyst/electrolyte interface, limits the growth of interfacial resistance, and preserves charge transfer kinetics over extended operation.

## 3 Conclusion

Herein, cation–anion co‐doping with Nb and F is employed to stabilize MnO_2_ under strongly acidic OER conditions while enhancing its intrinsic activity using a scalable anodic electrodeposition route on Pt‐coated Ti felt followed by postdeposition Nb/F incorporation. Comprehensive structural and spectroscopic characterization reveals that Nb/F co‐doping preserves the γ/β‐MnO_2_ tunnel framework while inducing lattice strain, reducing crystallite size, and stabilizing an electronically modified Mn environment characterized by an increased Mn^3+^/Mn^4+^ ratio together with enhanced defect‐rich oxygen coordination. These coupled structural and electronic effects suppress Mn overoxidation through strengthened Mn─O coordination and collectively generate a stabilized yet more catalytically active surface. Electrochemically, the MnO_2_‐Nb‐F catalyst exhibits reduced overpotentials at 10 and 100 mA cm^−2^, accelerated OER kinetics, and a lower *R*_ct_ compared with pristine and Nb‐only MnO_2_. Moving beyond half‐cell benchmarking, we demonstrate MEA operation in a single‐cell PEM electrolyzer, where the MnO_2_‐Nb‐F anode delivers the lowest cell voltages across the current densities tested, reaching 1.91 V at 1 A cm^−2^ and exhibiting the lowest HFR (174 mΩ cm^2^) among the MnO_2_ variants studied. Notably, the average degradation rate of the MnO_2_‐Nb‐F electrode (0.84 mV h^−1^) is three times lower than that of pristine MnO_2_ (2.6 mV h^−1^), demonstrating that Nb and F co‐doping effectively stabilizes the Mn‐O catalytic framework, suppresses rapid kinetic degradation, and enables nearly fourfold longer operational stability (380 h vs. 95 h) under PEM electrolyzer conditions. By establishing Nb/F co‐doping as an effective strategy to regulate Mn redox chemistry and enhance the stability of PGM‐free PEM‐WE anodes, this work highlights combined cation–anion incorporation as a promising approach for tuning the electronic structure and oxidative stability of transition‐metal oxide electrocatalysts under acidic conditions.

## Funding

This study was supported by Johannes‐Rau‐Forschungsgemeinschaft (JRF), Max‐Planck‐Gesellschaft.

## Conflicts of Interest

The authors declare no conflicts of interest.

## Supporting information

Supplementary Material

## Data Availability

The data that support the findings of this study are available from the corresponding authors upon reasonable request. The XAS data supporting this study are publicly available in the Edmond Open Research Data Repository athttps://doi.org/10.17617/3.85ZMIB.
