# Supplementary material for: Cation–Anion Co‐doping Enables Acid‐Stable MnO2 Anodes for Proton Exchange Membrane Water Electrolysis
Source: Small Sci. 2026 Aug 27;6(9):e70388. doi: 10.1002/smsc.70388 (PMC13533152; doi:10.1002/smsc.70388)
Supplement: Supplementary file 1 [file SMSC-6-e70388-s001.pdf]

Supporting Information

Cation-Anion Co-Doping Enables Acid-Stable MnO_2 Anodes for Proton Exchange Membrane Water Electrolysis

Vimanshu Chanda[a], Olaf Rüdiger*[b], Daniel Cruz[c], Viktor Mackert[a], Pascal Sous[a], Markus Sonnenberg[a], Hesham Solh[a], Lukas Pielsticker[b], Leander Kucklick[d,e], Walid Hetaba[b], Sebastian Daniel Hirt[a], Harry Ernst Hoster[a,d,e], Natalia Levin[a]*

[a] The Hydrogen and Fuel Cell Center (ZBT GmbH), Duisburg, Germany

[b] Max-Planck-Institute for Chemical Energy Conversion, Mülheim an der Ruhr, Germany

[c] Department of Inorganic Chemistry, Fritz-Haber-Institut der Max-Planck-Gesellschaft, Berlin, Germany

[d] Energy Technology, University of Duisburg-Essen, Duisburg, Germany

[e] Center for Nanointegration Duisburg-Essen (CENIDE), University of Duisburg-Essen, Duisburg, Germany

Experimental Section

Chemicals and materials

Manganese (II) sulfate monohydrate ($MnSO_4 \cdot H_2O$, ≥99%, Sigma-Aldrich), niobium(V) chloride ($NbCl_5$, 99.95%, Alfa Aesar), and ammonium fluoride (NH_4F, 98%, Alfa Aesar) were used as precursors for synthesizing electrocatalysts. Sulfuric acid (H_2SO_4, 98%, Sigma-Aldrich) was used for preparing electrodeposition solutions. A Nafion ionomer dispersion (10 wt%) in a mixed aliphatic solvent-water system (Sigma-Aldrich) was used as the binder for the preparation of the electrocatalyst ink. All aqueous solutions were prepared with ultrapure water of 18.2 MΩ cm^{-1} (Millipore purification system). All chemicals were used as received without any further purification.

Synthesis of MnO_2, MnO_2-Nb and MnO_2-Nb-F

The MnO_2 electrocatalysts reported in this work were synthesized by anodic electrodeposition followed by thermal treatment. MnO_2 films were deposited onto Pt-coated Ti felt substrates (Bekaert, 350 μm) via galvanostatic electrodeposition **(Figure S1a)**. The Pt-coated Ti felt substrates were ultrasonically cleaned in deionized water and ethanol to remove surface contaminants prior to electrodeposition. The process was conducted in an aqueous electrolyte containing 0.3 M $MnSO_4 \cdot H_2O$ and 0.35 M H_2SO_4, using a three-electrode configuration. A constant current density of 7 mA cm^{-2} was applied for 12 min, while the bath temperature was maintained at 94 °C, to promote uniform film growth. The electrodeposition proceeds via the oxidation of Mn^{2+} according to Equation (1):

$$Mn^{2+} + 2H_2O \rightarrow MnO_2 + 4H^+ + 2e^- \quad (1)$$

After deposition, the electrodes were rinsed thoroughly with deionized water and dried at 80 °C for 1 hour. The deposited MnO_2 films were subjected to annealing at 400 °C for 5 hours in air to improve crystallinity. The achieved mass loading of the MnO_2 catalyst on the electrode was 1.09 ± 0.08 mg cm^{-2} (n = 3). The MnO_2-Nb, MnO_2-F, and MnO_2-Nb-F catalysts were prepared by spray-coating the corresponding precursor solution onto the as-deposited MnO_2 electrode, followed by the same drying (80 °C, 1 h) and annealing (400 °C, 5 h, air) steps described above. For MnO_2-Nb, an aqueous solution of NbCl5 (75 mM) was used, with the precursor concentration and spray-coated volume adjusted to give a nominal Nb loading of ~0.5 mg cm^2 (Figure S1b). For MnO_2-Nb-F, a combined aqueous solution of $NbCl_5$ (75 mM) and NH_4F (22.5 mM) was used (Figure S1c). An analogous MnO_2-F control was prepared using NH_4F (22.5 mM) at a nominal loading matched to MnO_2-Nb, to isolate the contribution of F relative to Nb-F co-doping.

Physicochemical Characterization

XRD patterns were collected using a Rigaku SmartLab diffractometer in Bragg-Brentano geometry equipped with a Cu Kα radiation source (λ = 1.5418 Å). The data were recorded over a 2θ range of 10-80°. For XRD analysis, the deposited MnO_2 catalyst layer was carefully scraped from the substrate and ground into powder prior to measurement to minimize substrate contributions and ensure random crystallite orientation. A small amount of the powder was then placed on a low-background silicon wafer mounted in a PMMA sample holder. The near-surface composition of the as-prepared samples was investigated using X-ray photoelectron spectroscopy (XPS). Samples were mounted on carbon tape with full surface coverage to avoid signal contributions from the underlying substrate and subsequently transferred into the XPS chamber. An ultra-high vacuum (UHV) system (base pressure $< 5 \times 10^{-10}$ mbar; SPECS GmbH) equipped with a Phoibos NAP-150 hemispherical analyzer (SPECS GmbH) and a monochromated Al Kα X-ray source (hν = 1486.6 eV) was used for all measurements. The source–analyzer angle was 54°, and the sample stage was oriented normal (90°) to the analyzer. Charge compensation during analysis was achieved using an electron flood gun (EQ 22/35, SPECS GmbH) operated at an acceleration voltage of 200 V. Survey spectra and high-resolution spectra of the C 1s, O 1s, Mn 2p, and Nb 3d regions were recorded. Binding energies were calibrated using the adventitious C 1s peak at 284.8 eV as a reference. Data processing and peak fitting were carried out using CasaXPS software (version 2.3.26). Background subtraction was performed using the Shirley method, and symmetric GL(60) gaussian-lorentzian product line shapes were applied for fitting all core-level spectra. Peak fitting of the Mn 2p spectra was performed using literature-reported multiplet splitting parameters for Mn species in transition metal oxides, following the approach described by Biesinger *et al*.[1] In this method, each oxidation state is represented by a characteristic multiplet-split envelope together with the associated satellite structure. The fitting components were constrained according to literature-reported values for peak positions, relative intensities, spin–orbit splitting, and line shapes rather than treated as fully independent fitting parameters. This fitting procedure was used to distinguish Mn^{3+} and Mn^{4+} contributions and evaluate changes in the surface electronic structure upon Nb and F incorporation. SEM was carried out using a FEI Quanta 400 ESEM operated in high-vacuum mode, and elemental analysis was performed by EDX using a Genesis 4000 detector. HR-TEM, HAADF-STEM as well as STEM-EDX mapping were performed on a Thermo Scientific Talos F200X G2 microscope operated at 200 kV and equipped with a Super-X EDX system. Metal dissolution was assessed by ICP-OES of electrolyte samples collected before and after the 250 h chronopotentiometric stability test at 10 mA cm^{-2} in 0.5 M

H_2SO_4. Measurements were performed on a SPECTROBLUE ICP-OES instrument (AMETEK SPECTRO), using the emission lines at 257.611 nm (Mn) and 309.418 nm (Nb). The full spectral regions surrounding each line were inspected in addition to the target wavelengths to distinguish genuine emission peaks from background signal. To minimise memory effects and cross-contamination, the instrument tubing and nebulizer system were flushed with Milli-Q water (18.2 MΩ·cm) between all measurements. The analytical sequence comprised an instrument blank (Milli-Q water), the pre-experiment electrolyte samples (MnO_2, MnO_2–Nb, MnO_2–Nb–F), a second instrument blank, the corresponding post-experiment samples, and a final blank. Mn concentrations were quantified against a calibration curve. For Nb, emission intensities at 309.418 nm did not exceed the background signal observed in the blanks and electrolyte samples, and no distinguishable Nb emission peak was identified upon spectral inspection of the post-experiment samples. Nb dissolution was therefore below the quantifiable range of the present ICP-OES setup and could not be reliably determined.

Depth-Resolved Synchrotron XPS and XAS

Depth-resolved photoemission and X-ray absorption were recorded at the BElChem-PGM plane- 1200 mm grating-monochromator beamline of the BESSY II storage ring (Helmholtz-Zentrum Berlin für Materialien und Energie, Berlin, Germany). The tunable, linearly polarized soft X-ray source enables continuous, non-destructive depth profiling. By varying the photon energy (hν), we control the kinetic energy, and thus the inelastic mean free path and information depth, of photoelectrons from each core level, avoiding Ar^+ sputtering, which can preferentially remove F and chemically reduce Mn.

Core-level spectra (Mn 2p, Nb 3d, O 1s, F 1s) and the valence band were acquired with a hemispherical analyzer in fixed-analyzer-transmission (FAT) mode at pass energy 10 eV. Residual Cl 2p, arising from unreacted $NbCl_5$ precursor, was resolved under these synchrotron excitation conditions owing to the enhanced surface sensitivity and more favorable photoionization cross-section at low photon energy, and was not detected under laboratory Al Kα conditions. For the F 1s series, five photon energies were used so that F 1s emerged at KE = 200, 400, 800, 1000 and 1110 eV, giving information depths (3λ) of 21, 32, 52, 61 and 66 Å (≈ 2–7 nm; Table A1). The F K-edge (≈ 685 eV) was additionally recorded in X-ray absorption mode in two detection channels of complementary depth sensitivity: Auger-electron yield (AEY, very surface sensitive, ≈ 1–2 nm) and total-electron yield (TEY, more bulk sensitive, ≈ 5–10 nm).

The synchrotron XPS survey spectrum (Figure S17), acquired at 1200 eV excitation energy, shows an intense F 1s photoemission line at ≈685 eV alongside Mn 2p, O 1s, Nb 3d, Cl 2p, and C 1s core levels, providing direct and unambiguous detection of fluorine not resolved in the laboratory survey. Independent, element-specific confirmation is provided by F K-edge XANES in two detection modes **(Figures S18 and S19)**: the surface-sensitive Auger-electron-yield (AEY, ≈1–2 nm) and more bulk-sensitive total-electron-yield (TEY, ≈5–10 nm) channels both show a clear F K-edge (onset ≈688 eV) with a characteristic metal-fluoride/fluoro-oxide white-line. Because the edge persists in the more bulk-sensitive TEY channel, fluorine is confirmed to extend into the sub-surface region in a metal-bonded environment rather than existing only as an adventitious surface film, consistent with the depth-resolved F 1s trend (Figure S16): the covalent surface fluoride is surface-enriched while the ionic lattice fluoride grows inward.

To determine whether Nb, F, and reduced Mn are confined to the surface or genuinely incorporated into the lattice, a sensitivity-normalized depth-contrast factor was defined as $R = [I(3\lambda = 61\ Å)/S] / [I(3\lambda = 21\ Å)/S]$, where I/S is the depth-weighted, sensitivity-normalized intensity of a given species. By this definition, $R < 1$ indicates surface enrichment, $R \approx 1$ a uniform distribution, and $R > 1$ subsurface/bulk enrichment; a surface-adhered NbO_x or Nb/F film would be expected to give $R < 1$. **Figure S20** shows the opposite trend for every lattice-type species considered: Nb/(F+O+Mn+Nb) gives $R \approx 10.2$, the ionic (Mn–F, Nb–F) fraction gives $R \approx 1.9$, and the Mn^{3+}/Mn^{4+} ratio gives $R \approx 9.4$, indicating that Nb, ionic fluoride, and reduced Mn are all subsurface-enriched rather than surface-concentrated. This is consistent with substitutional lattice incorporation and with the absence of any Nb_xO_y reflection in the XRD. **Figure S21** presents the same trends in a transmission-independent form (intra-spectrum ratios, unaffected by absolute flux, cross-section, or IMFP): the ionic fluoride fraction of total F rises from 10.5% to 20.7%, while the Nb atomic fraction and the Mn^{3+}/Mn^{4+} ratio (0 → ≈0.47) both increase monotonically with depth. **Figure S22** summarizes this picture schematically: a thin (~1–2.5 nm) hydroxylated, fluorine-poor outer skin overlying an Nb(V)-, lattice-F-, and Mn^{3+}-enriched interior. Only the absolute magnitude of these depth gradients carries residual uncertainty; the direction of every trend, and all intra-spectrum ratios reported here, are transmission-independent and therefore robust.

The elevated near-surface Nb fraction reported by conventional (surface-weighted) XPS quantification reflects this hydroxylated outer skin rather than evidence of a discrete Nb overlayer, since the depth-resolved data (**Figures S15, S20, S21**) show Nb increasing, not decreasing, toward the interior. Regarding the uniformity of F incorporation, **Figure S21** shows

a finite ionic (lattice) F fraction present at every probed depth, rising monotonically from 10.5% to 20.7%, consistent with the distributed F observed in the HAADF-STEM elemental maps **(Figure S11)**. Rather than claiming atomically uniform doping, the data supports a graded distribution: covalent/surface fluoride concentrated in the outermost nanometer, with lattice fluoride increasing toward the interior. This is independently corroborated by the depth-resolved Mn 2p and O 1s series (**Figures S23, S24**), which show the interior to be more reduced (higher Mn^{3+} fraction) and more oxidic (higher lattice O^{2-} fraction), consistent with dopant incorporation beneath a thin hydroxylated surface layer.

Electrochemical operando XAS

Operando measurements were conducted at the ID24-DCM beamline of the ESRF synchrotron, operating with an electron beam current of 200 mA. The incident energy was selected by a Si (111) double crystal monochromator with Pt-coated mirrors. Incident flux was ca. 1 x 10^{13} ph/sec using a beam size of 0.3 mm × 0.3 mm. Fluorescence spectra were captured using a diode detector. The electrodes were prepared analogously to what is described in the previous section, but using a 40 mm diameter carbon felt disks. The Pt/Ti substrate could not be employed in this experiment, since it would block completely the X-rays. The disks were placed in a custom-built spectroelectrochemical (SEC) flow-cell **(Figure S25)**. The schematics for the cell can be accessed via the Edmond data repository. Throughout the measurement, the electrolyte (0.5 M H_2SO_4) was continuously circulated through the cell (0.5 mL/min) using a pressure-controlled pump (Fluigent) to prevent electrode vibrations that may induce noise in the spectra. A SP-50 potentiostat (Biologic) was used for potential control of the experiments **(Figure S26)**. We tested for beam damage by repeating measurements at different beam attenuations. Subtle spectral changes were observed when the measuring time at a single spot exceeded 90 s with an attenuation factor of 100. To monitor and prevent beam damage, we measured 20 s scans and moved to a fresh spot after 3 scans. The incident energy was calibrated by assigning the first inflection point of a Mn foil to 6539 eV, and the Nb foil energy was calibrated to 18986 eV. Final spectra were processed and normalized using Athena included in the DEMETER package.[2]

Electrochemical experiments

Electrochemical measurements were performed using a three-electrode configuration with a platinum mesh counter electrode (wire diameter: 0.5 mm), Ag/AgCl (3.4 M KCl), (Innovative Instruments) as reference electrode (Innovative Instruments, USA) and 0.5 M sulfuric acid (H_2SO_4, pH ~0.22) as the electrolyte. The reference electrode potential was regularly verified

against a commercial reference electrode. During measurements, the reference electrode was positioned at a fixed distance from the working electrode surface. Linear sweep voltammetry (LSV) and chronopotentiometry (CP) measurements were conducted using a Gamry 5000E potentiostat. The current density was calculated based on the geometric area of the working electrode (1 cm^2) exposed to the electrolyte. All potential values measured in this study were converted to RHE scale using equation 2:

$$E_{iR}(\mathrm{RHE}) = E_{\mathrm{Ag/AgCl\ (3.4\ M\ KCl)}} + 0.21 + (0.059.\mathrm{pH} - \mathrm{i}.Ru) \quad (2)$$

where E_{iR} is the electrode potential in the RHE (in V); $E_{\mathrm{Ag/AgCl(3.4\ M\ KCl)}}$ is the measured potential versus Ag/AgCl/3.4 M KCl (in V) and 0.21 is the standard potential of the Ag/AgCl (3.4 M KCl) reference electrode. E_{iR} values were manually 100% *iR*-corrected using the uncompensated resistance (*R*u, Ω) calculated by GEIS in the high-frequency range.

LSV curves were recorded at a scan rate of 1 mV s^{-1} to minimize the capacitive contribution arising from the pseudocapacitance of MnO_2. Prior to activity measurements, the electrodes were conditioned by cyclic voltammetry (scan rate: 100 mV s^{-1}; potential range: 1.0-1.4 V vs. RHE) to stabilize the catalyst surface. The EIS was also performed using the Gamry 5000E in 0.5 M H_2SO_4 electrolyte solution at 1.75 V vs RHE and using a frequency range of 100 kHz-0.1 Hz (amplitude - 10 mV). For long-term chronopotentiometry experiments and electrolyzer testing, the electrodeposition time was increased to 1 h, resulting in a thicker catalyst layer with a mass loading of ~7.5 mg cm^{-2}. The data obtained from CP stability tests were not *iR* corrected and displayed as raw data. Tafel slopes were derived from LSV curves at a low overpotential fitted to the tafel equation (3):

$$\eta = a + b \cdot \log \mathrm{j} \quad (3)$$

where η is the overpotential, a is the constant, b is the Tafel slope, and j is the current density.

PEM electrolyzer setup and testing

The membrane electrode assemblies (MEAs) were fabricated using Nafion 115 polymer membranes (N-115, Fumatech) and underwent a pretreatment process to ensure complete protonation and remove potential contaminants. This process involved sequentially boiling the membranes in 3 wt.% H_2O_2 (Milli-Q ultrapure water) for 1 hour, followed by 1.0 M H_2SO_4 for 1 hour, and finally rinsing them in Milli-Q ultrapure water for 1 hour. The anode was prepared by coating the electrocatalyst onto a 350 µm Pt-coated Ti felt (Bekaert), followed by applying ~0.5 mg cm^{-2} of Nafion ionomer before assembling it into the MEA. The cathode consisted of Pt/C (20 wt% Pt on carbon, Heraeus) deposited directly onto the Nafion 115 membrane

following the catalyst-coated membrane (CCM) approach, with a Pt loading of ~0.2 mg cm^{-2}. The cathode slurry was formulated by mixing Pt/C with water, isopropanol, and Nafion dispersion (10 wt.% in H_2O, Sigma-Aldrich), maintaining a 3:1 mass ratio of Pt/C to Nafion ionomer **(Figure S31)**. The MEAs were assembled by hot-pressing the cathode and anode onto the Nafion membrane at 135 °C under a force of 5 kN for 3 minutes **(Figure S32)**. For benchmarking measurements, commercial IrO_x (40 wt% IrO_x on TiO_2, Heraeus) was used as the reference catalyst. The IrO_x reference catalyst had a mass loading of ~1 mg cm^{-2} for the three-electrode chronopotentiometric stability test (10 mA cm^{-2}, 250 h, 0.5 M H_2SO_4), and at ~2.5 mg cm^{-2} for the long-term single-cell PEM-WE test. The same IrO_x reference catalyst was used across both the three-electrode and single-cell PEM-WE experiments, so that all activity and stability comparisons to MnO_2, MnO_2-Nb, and MnO_2-Nb-F are made under identical measurement conditions within each test format. Prior to electrochemical testing, the assembled MEA was conditioned by circulating heated deionized water through the electrolyzer to ensure full membrane hydration and stabilization of the electrode layers. The electrochemical tests were conducted in a single-cell PEM electrolyzer (Baltic Cell, Fuel Cell Works) with an active area of 5 cm^2. Polarization curves, galvanostatic EIS and durability measurements were performed using a Gamry 5000E potentiostat equipped with a booster. The cell and feed water temperatures were maintained at 60 °C under ambient pressure, with deionized water supplied to the anode at a flow rate of 10 mL min^{-1} cm^{-2}. A mixed-bed ion-exchange deionizer (LEYCO HLA-EK-2000-MB, LEYCO Wassertechnik GmbH) was integrated into the recirculating water loop, purifying the product effluent before it returned to the cell as feed and continuously removing trace ionic impurities to maintain high water purity during operation **(Figure S33)**. This protocol, including feed-water resistivity, ion-exchange column configuration, flow rate, and cell/feed-water temperature, was applied identically across all single-cell measurements reported in this work, ensuring that relative comparisons between catalysts are not confounded by differing water-quality protocols.

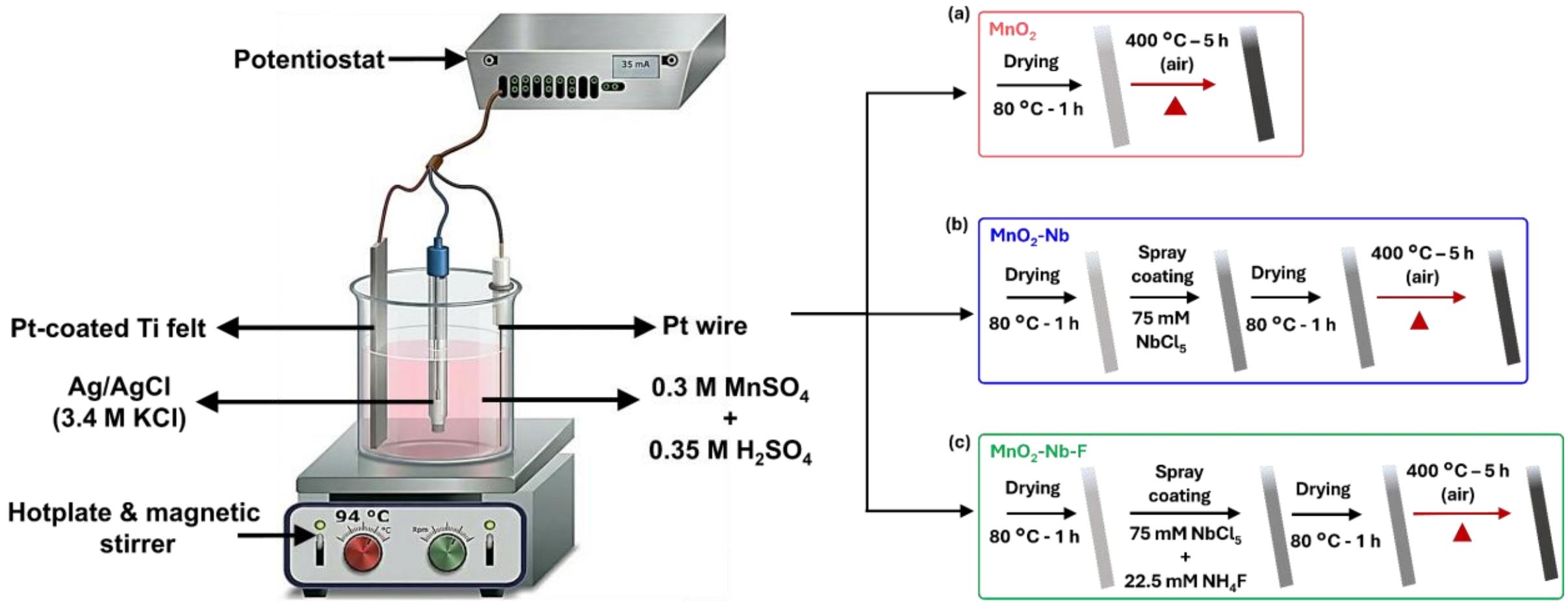

Figure S1. Schematic representation of the synthesis for (a) MnO_2, (b) MnO_2-Nb and (c) MnO_2-Nb-F electrocatalysts.

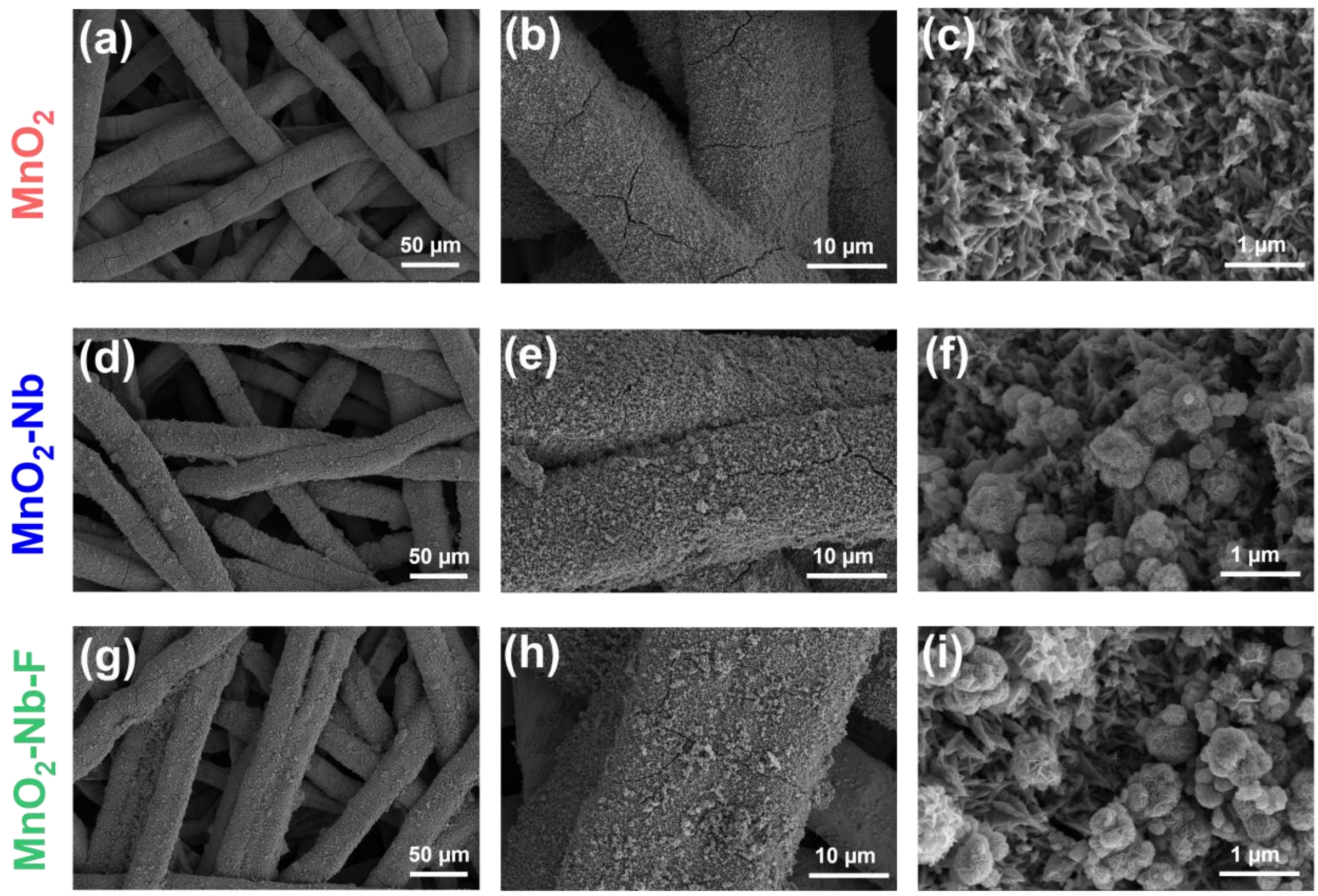

Figure S2. Scanning electron microscopy (SEM) images of (a-c) MnO_2, (d-f) MnO_2-Nb, and (g-i) MnO_2-Nb-F at different magnifications.

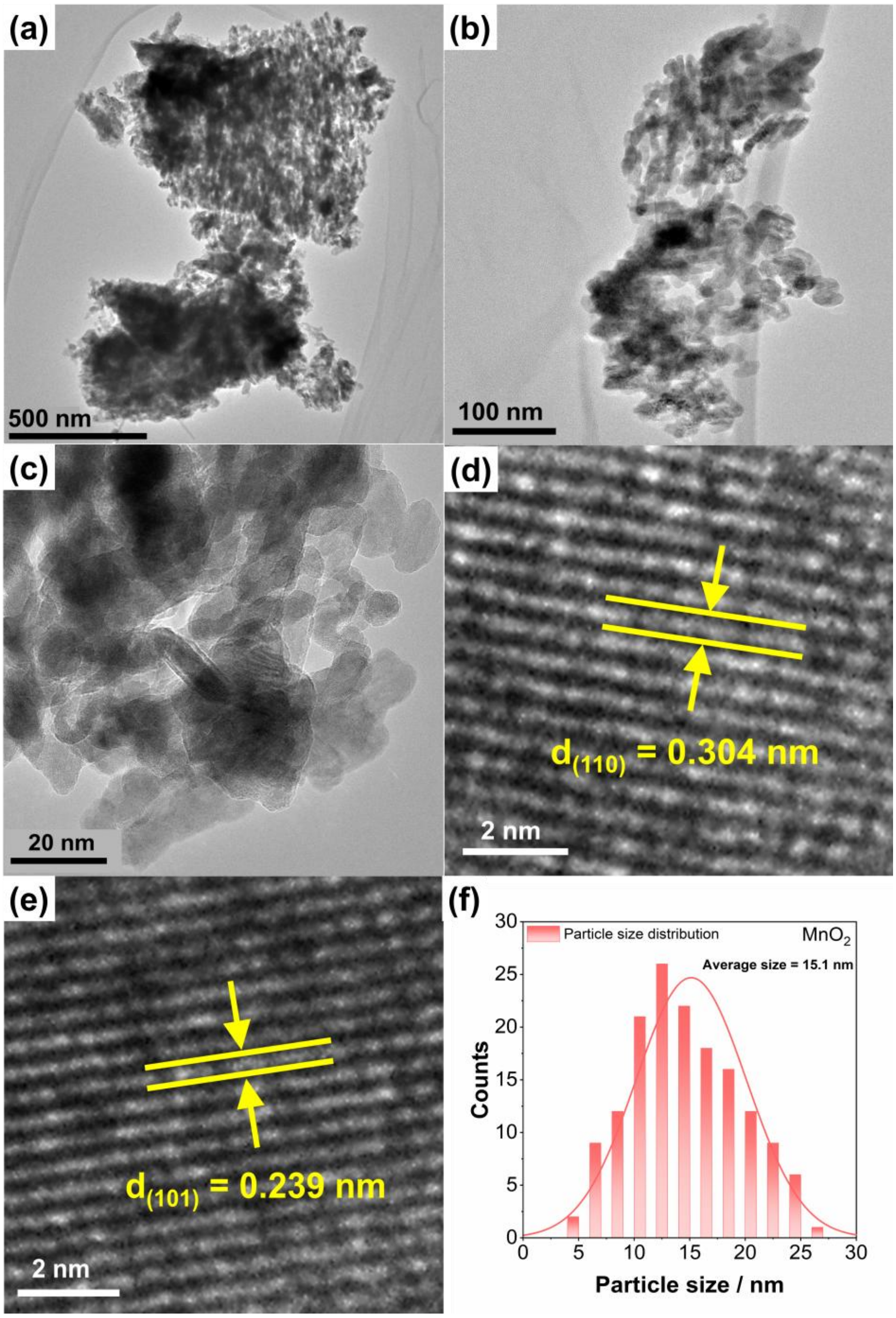

Figure S3. Morphology characterization of the MnO_2. (a-c) TEM images at progressively higher magnifications reveal a nanostructured morphology composed of aggregated yet well-dispersed MnO_2 nanoparticles. (d) High-resolution TEM (HR-TEM) image displays distinct lattice fringes with an interplanar spacing of 0.304 nm, corresponding to the (110) plane of MnO_2. (e) HR-TEM image displays distinct lattice fringes with an interplanar spacing of 0.239 nm, corresponding to the (101) plane of MnO_2. (f) Particle size distribution derived from TEM analysis, showing an average particle size of 15.1 nm.

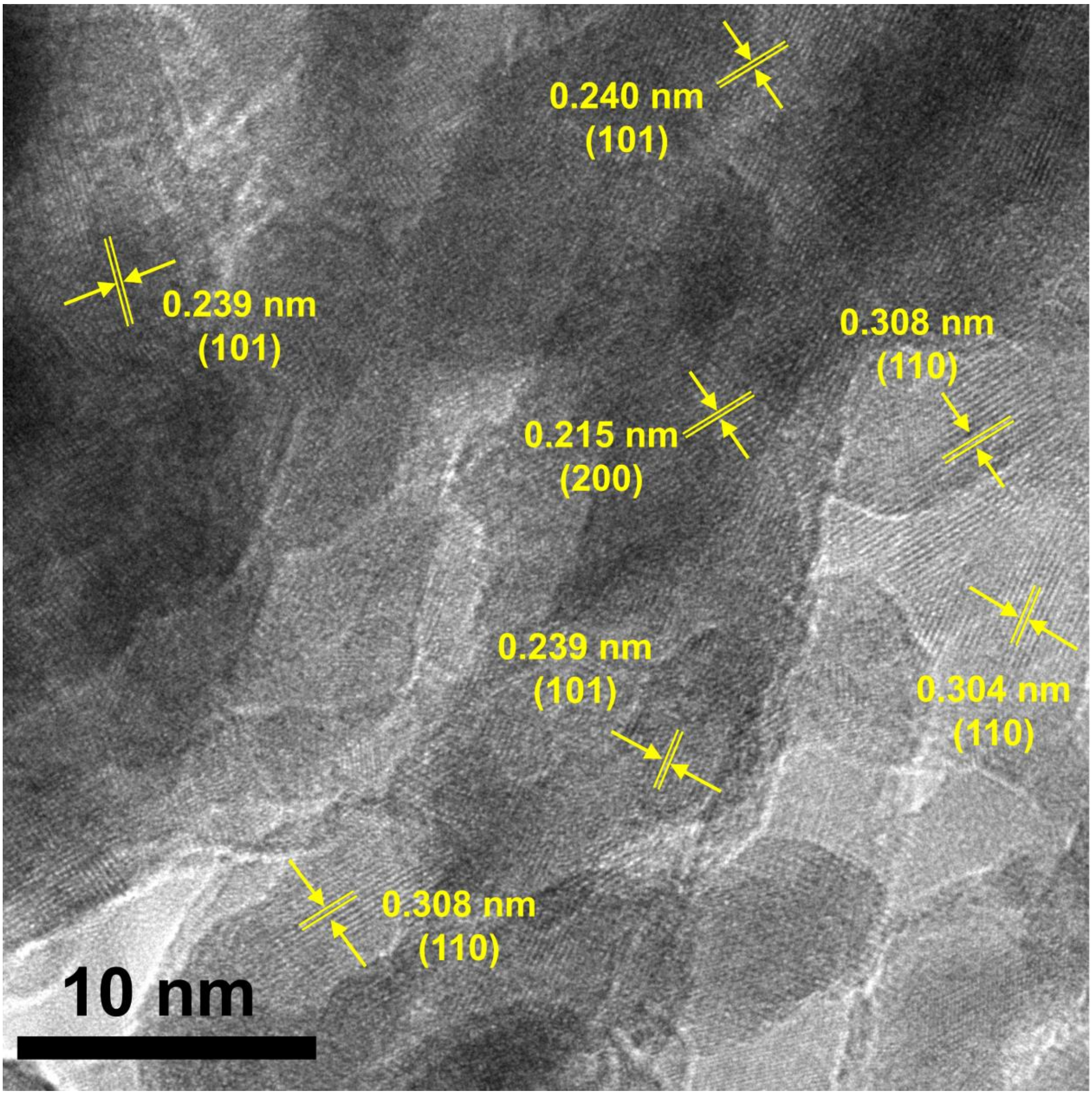

Figure S4. HR-TEM image for MnO_2. The image shows typical (110) and (101) crystal planes of MnO_2.

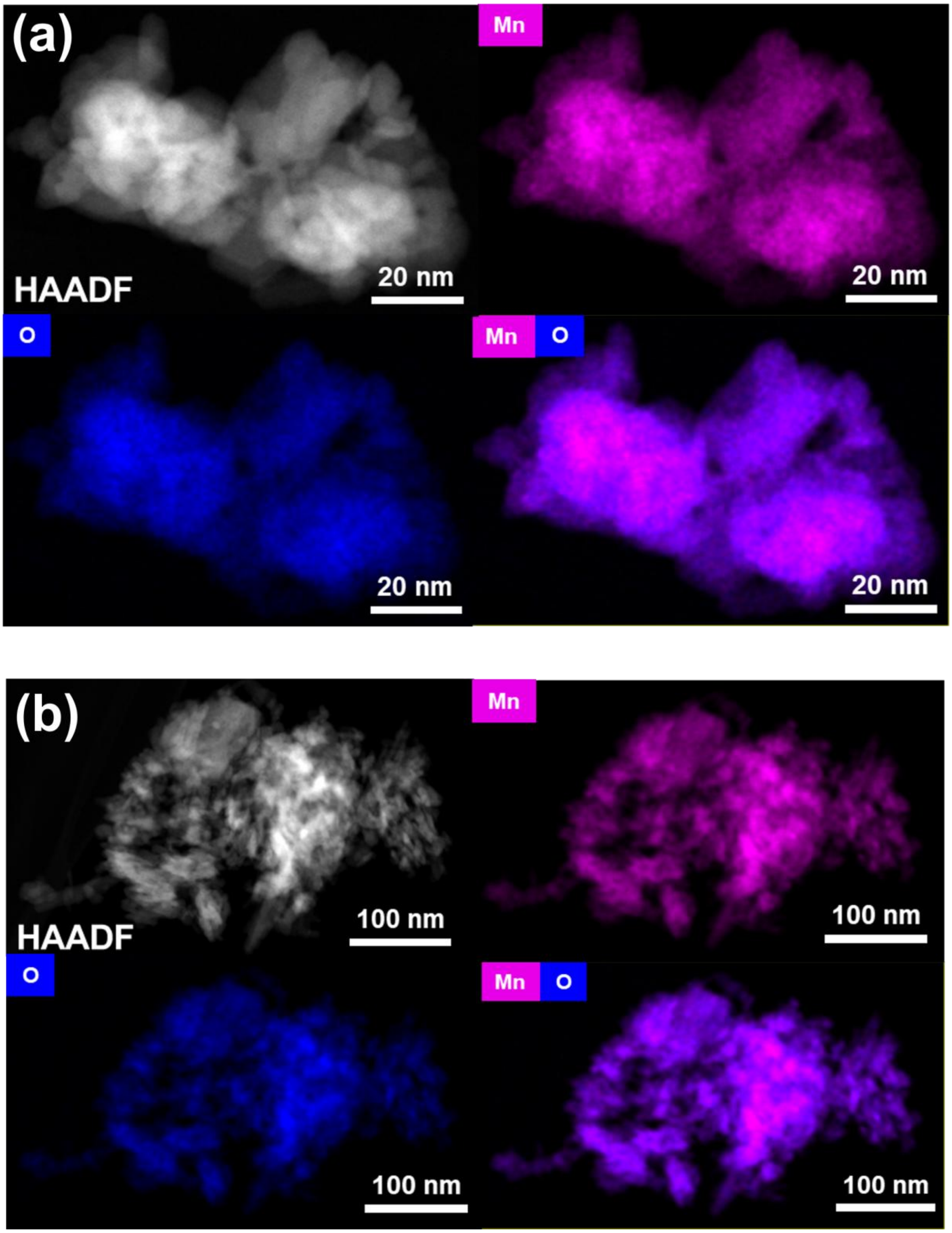

Figure S5. HAADF-STEM and corresponding EDX elemental maps of the pristine MnO_2 at (a) higher and (b) lower magnifications, showing a uniform distribution of Mn (magenta) and O (blue) throughout the nanostructure.

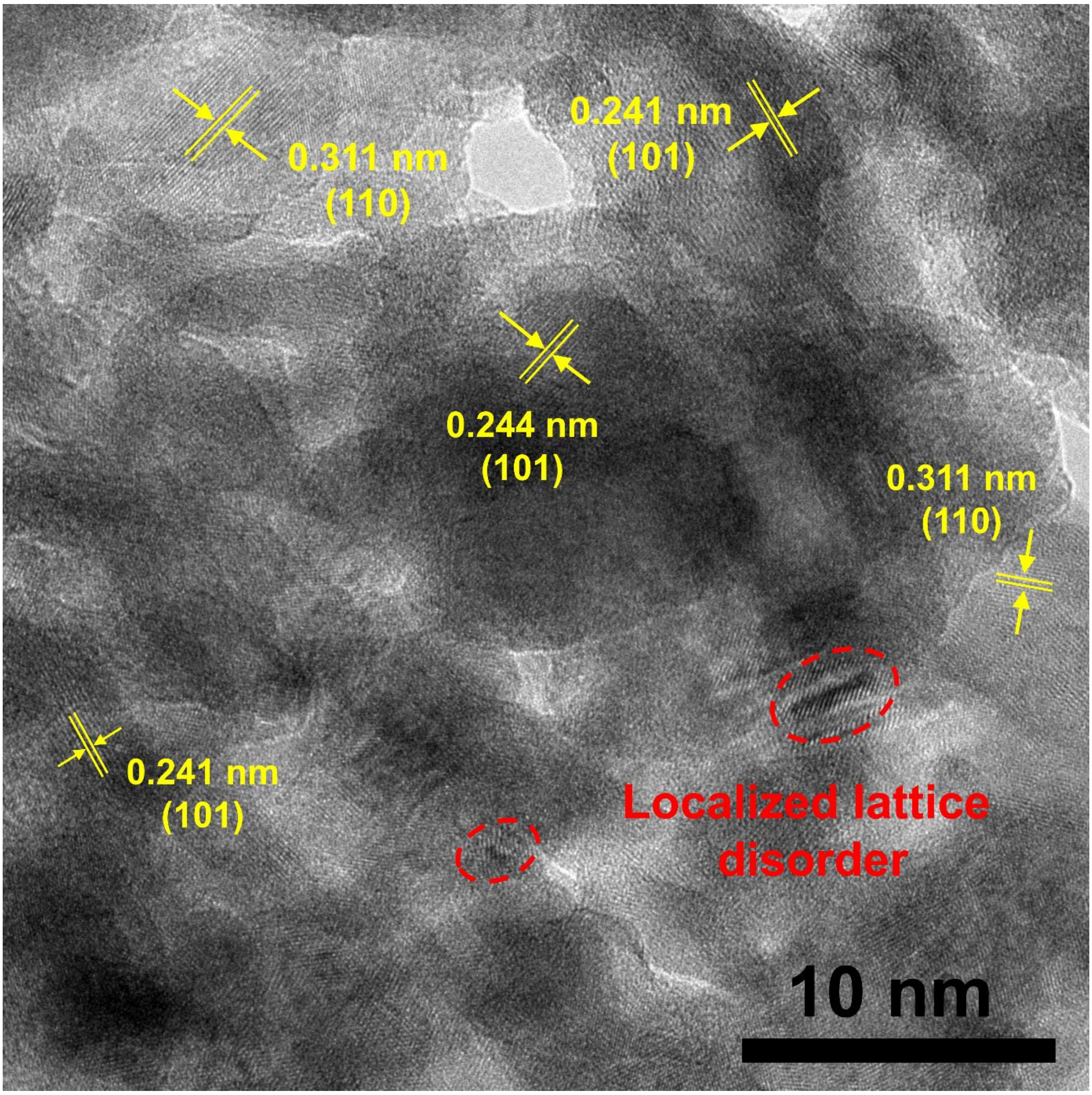

Figure S6. HR-TEM image for MnO_2-Nb. Regions highlighted with red circles indicate local lattice distortions and changes in interplanar distances, suggesting structural disorder and strain within the nanocrystalline domains.

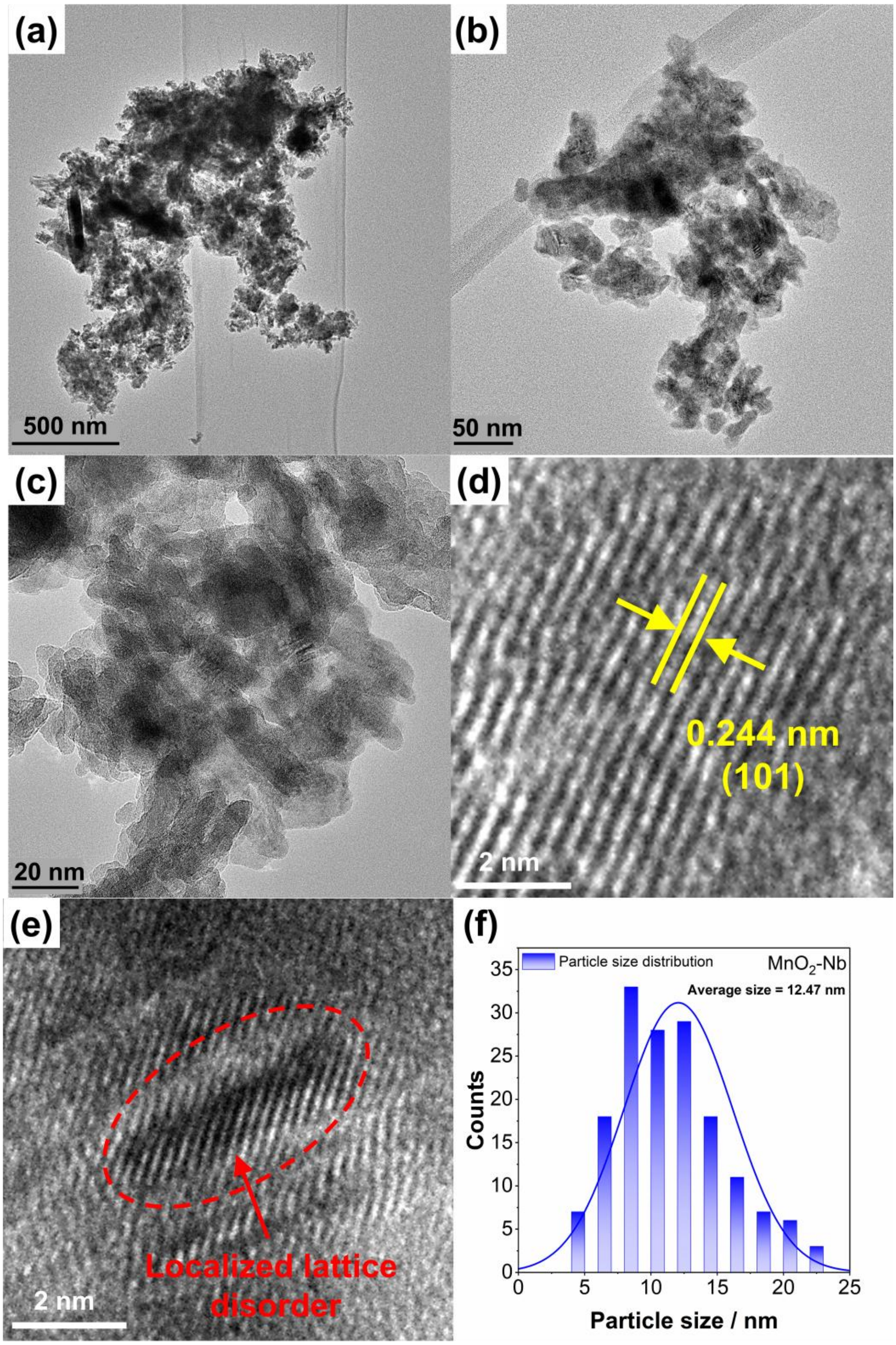

Figure S7. Morphology characterization of the MnO_2-Nb. (a-c) TEM images at progressively higher magnifications. (d) HR-TEM image displaying lattice fringes with an interplanar spacing of 0.244 nm corresponding to the (101) plane of MnO_2. (e) A locally distorted region indicating Nb-induced lattice disorder (highlighted in red). (f) Particle size distribution derived from TEM analysis, showing an average particle size of ~12.5 nm.

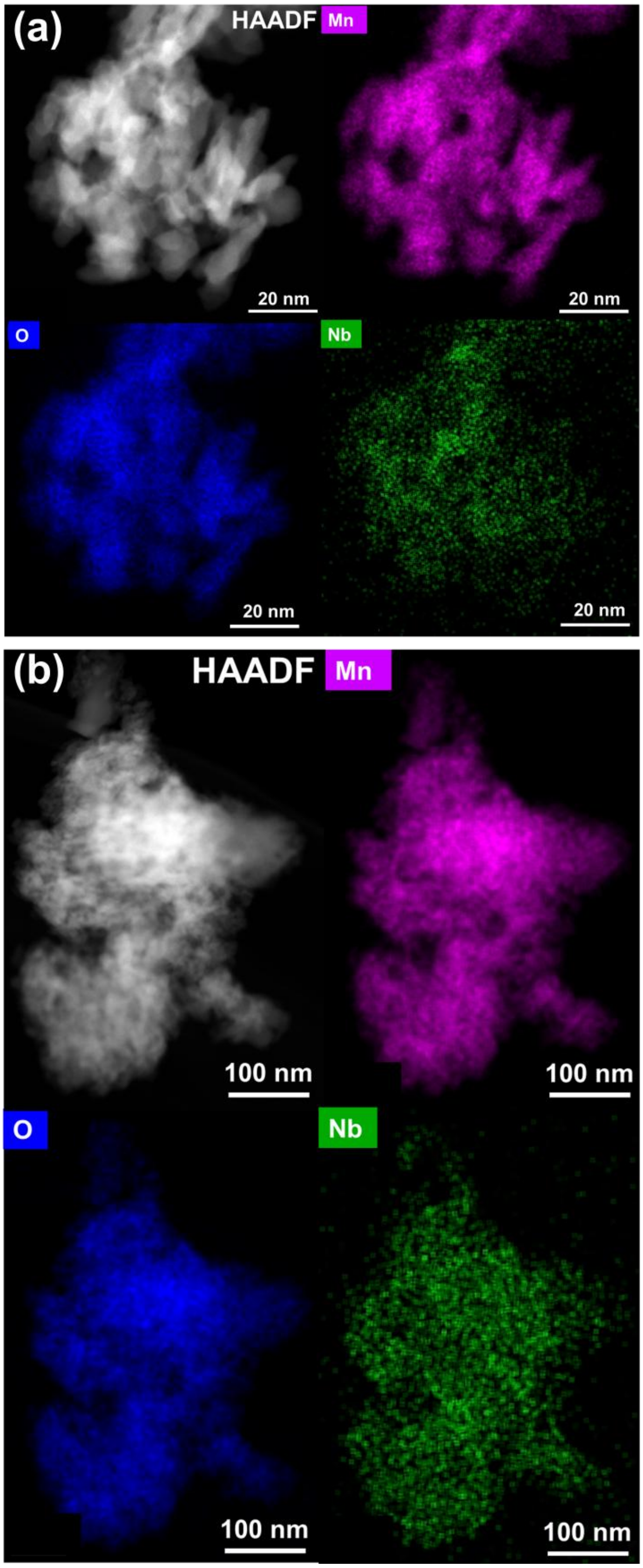

Figure S8. HAADF-STEM and corresponding EDX elemental maps of the MnO_2-Nb at (a) higher and (b) lower magnifications, showing a uniform distribution of Mn (magenta), O (blue) and Nb (green) throughout the nanostructure.

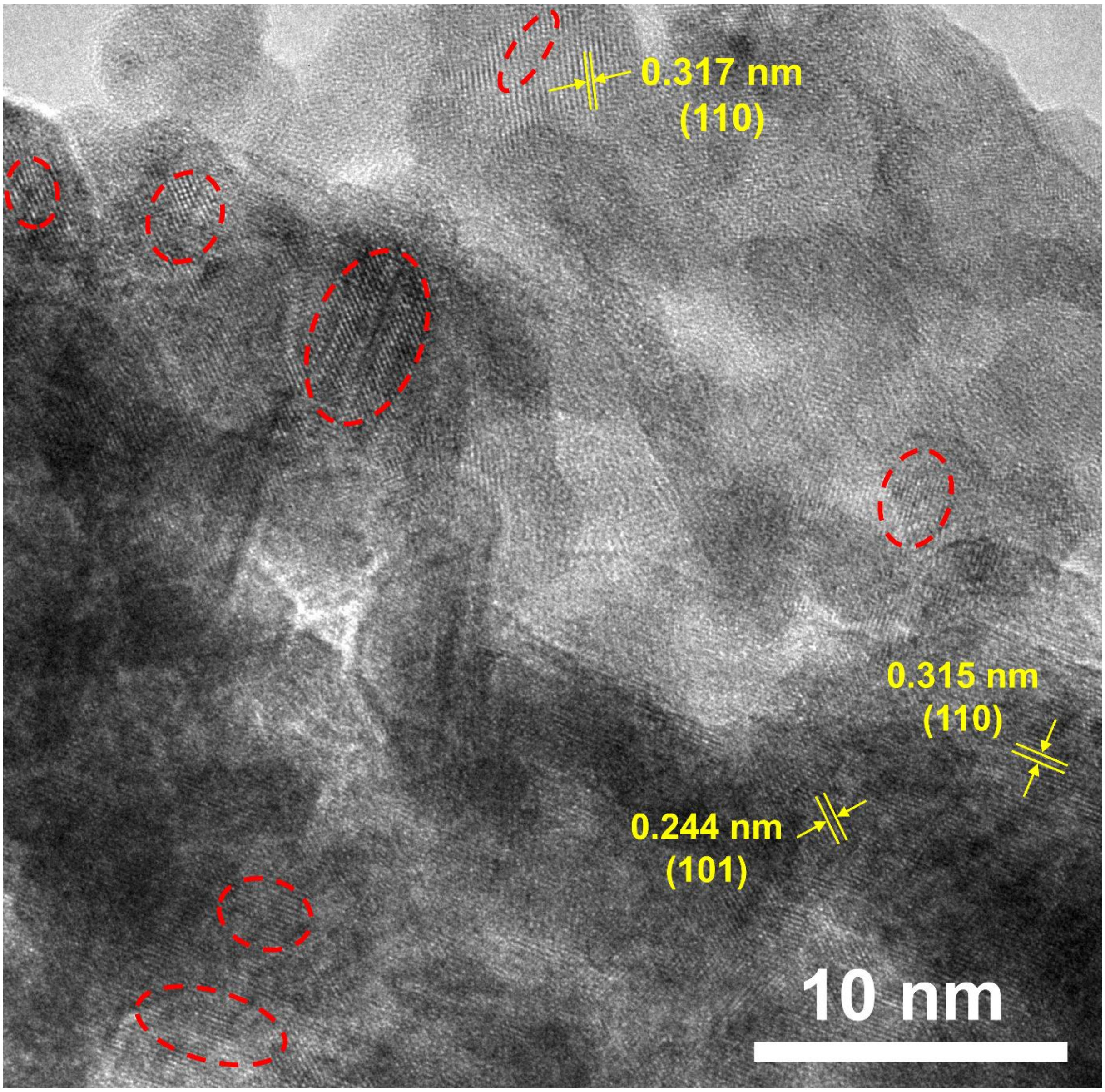

Figure S9. HR-TEM image for the MnO_2-Nb-F. Regions highlighted with red circles indicate local lattice distortions.

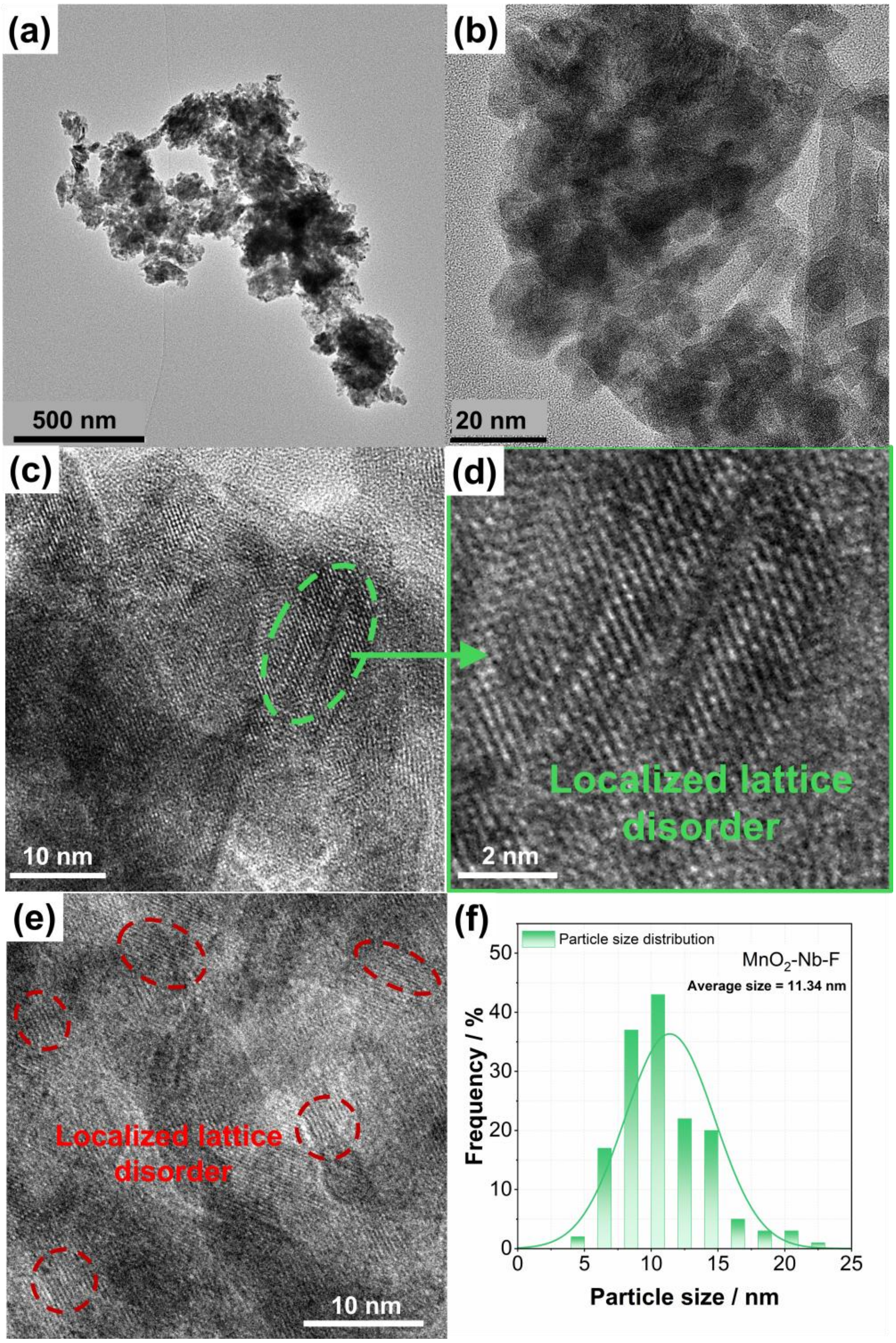

Figure S10. Morphology characterization of the MnO_2-Nb-F. (a-b) TEM images at progressively higher magnifications. (c) HR-TEM image showing lattice fringes corresponding to MnO_2 domains embedded within partially disordered regions. (d) Enlarged HR-TEM image highlighting localized lattice disorder. (e) A locally distorted region indicating Nb/F-induced lattice disorder. (f) Particle size distribution derived from TEM analysis, showing an average particle size of ~11.3 nm.

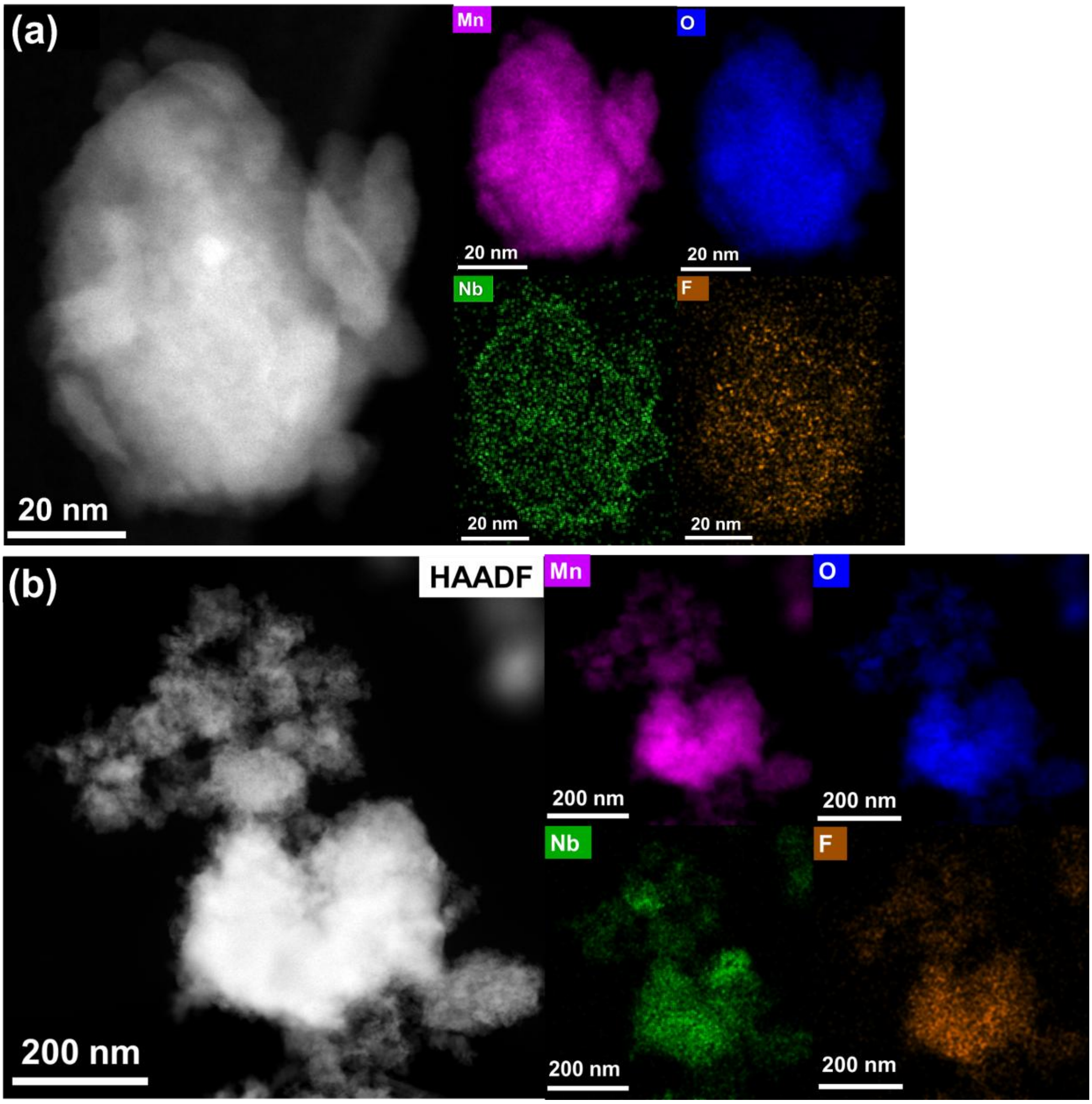

Figure S11. HAADF-STEM and corresponding EDX elemental maps of the MnO_2-Nb-F at (a) higher and (b) lower magnifications, showing a uniform distribution of elements throughout the nanostructure.

Table S1. EDX quantification (N = 3) of the three electrocatalysts, where N is the number of spots measured.

Sample	Mn (at. %)	O (at. %)	Nb (at. %)	F (at. %)
MnO_2	28.9 ± 0.3	71.1 ± 1.7	0	0
MnO_2-Nb	26.3 ± 4.5	66.5 ± 2.2	7.2 ± 1.8	0
MnO_2-Nb-F	25.4 ± 2.1	65.3 ± 4.8	5.8 ± 1.3	3.5 ± 1.8

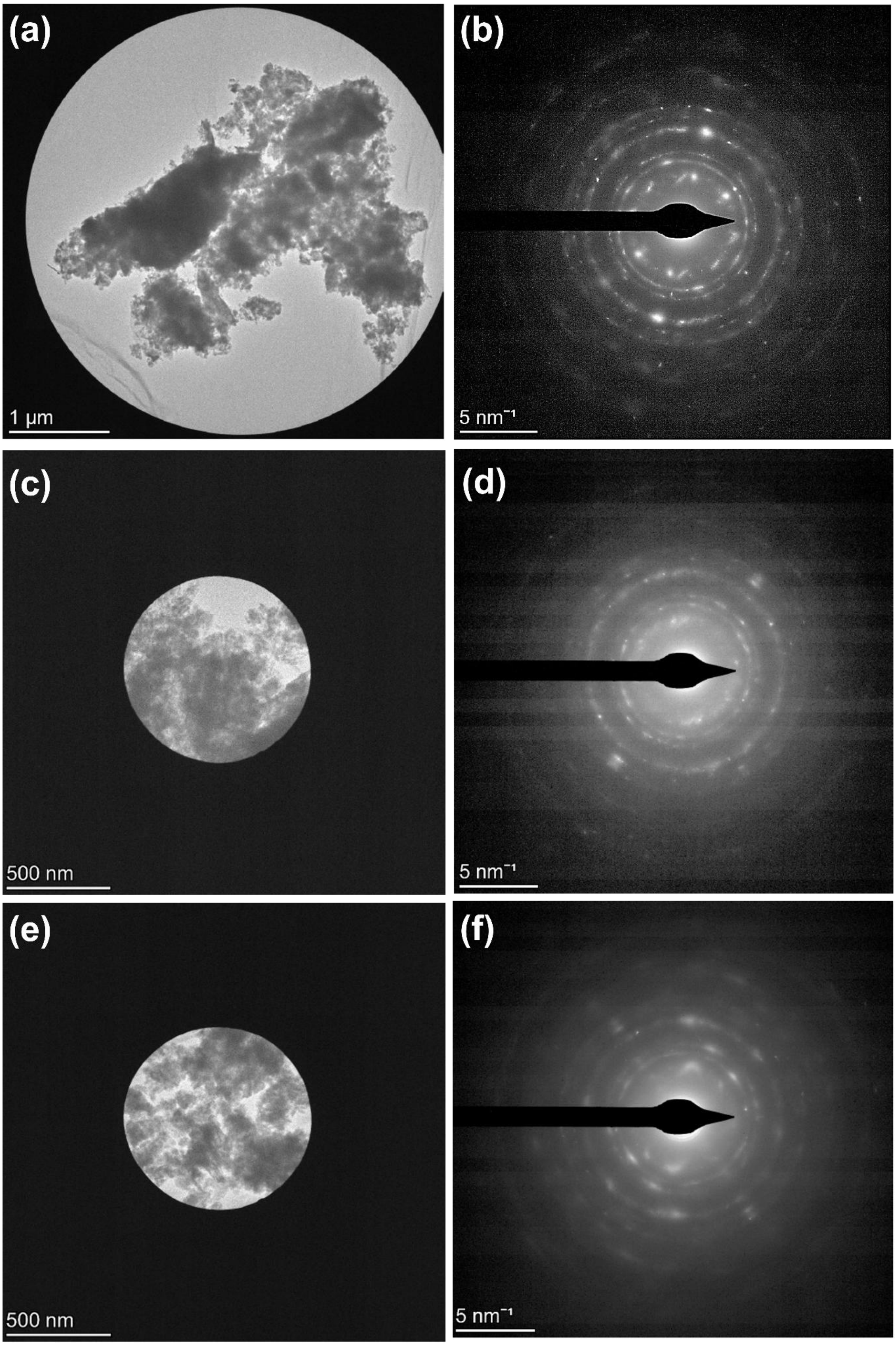

Figure S12. TEM images and the corresponding selected area electron diffraction (SAED) patterns for (a, b) MnO_2 (c, d) MnO_2-Nb and (e, f) MnO_2-Nb-F.

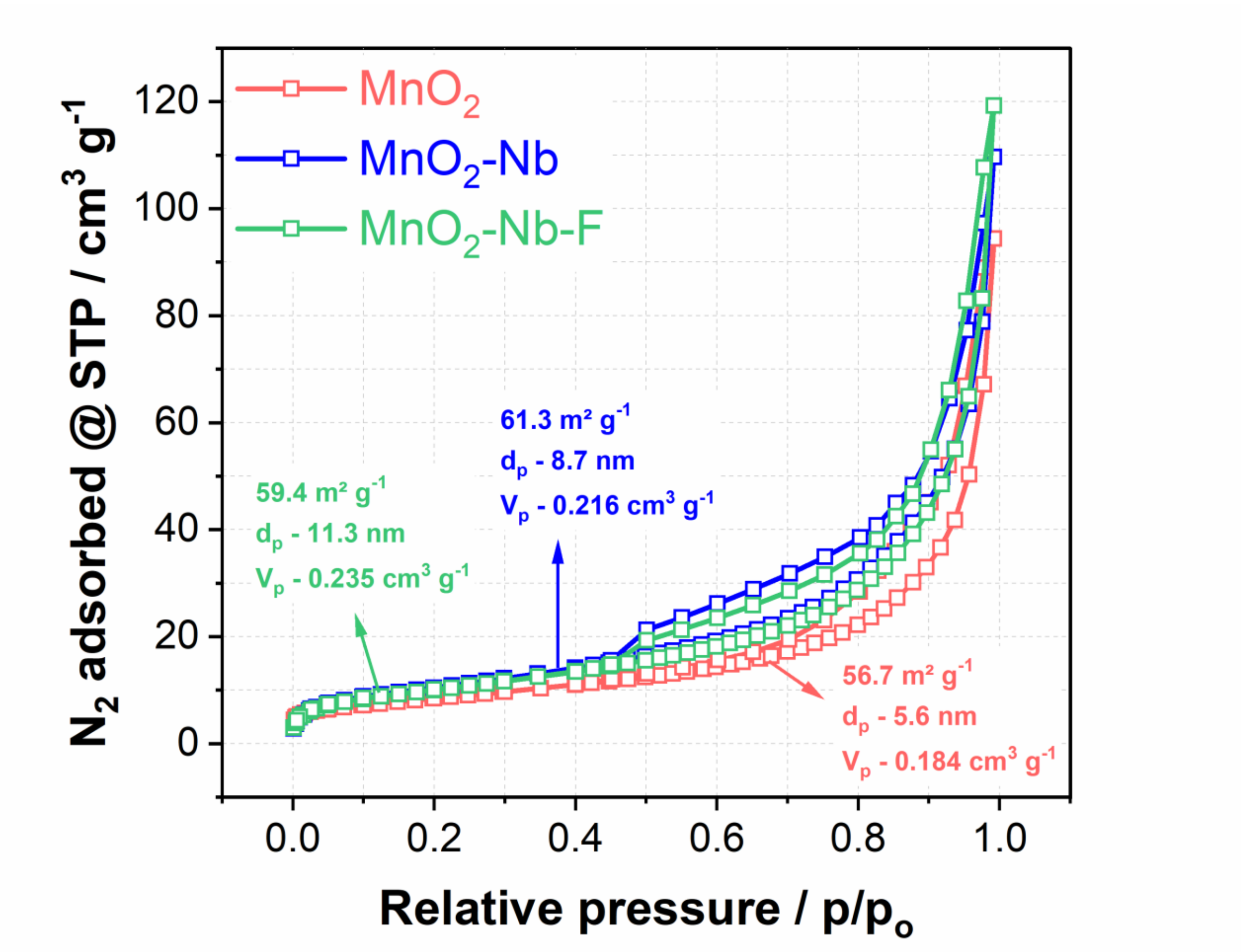

Figure S13. N_2 sorption desorption isotherms for MnO_2, MnO_2-Nb, and MnO_2-Nb-F.

Table S2. XPS quantification of the three electrocatalysts. The C 1s signal is attributed to adventitious carbon from ambient air exposure and is not part of the intrinsic catalyst composition; F in MnO_2-Nb-F was below the detection limit (n.d.).

Sample	C (at. %)	Mn (at. %)	O (at. %)	Nb (at. %)	F (at. %)
**MnO_2**	19.7	20.6	59.7	0.0	0.0
**MnO_2-Nb**	18.0	14.9	57.4	9.7	0.0
**MnO_2-Nb-F**	26.7	12.3	54.1	6.9	n.d.

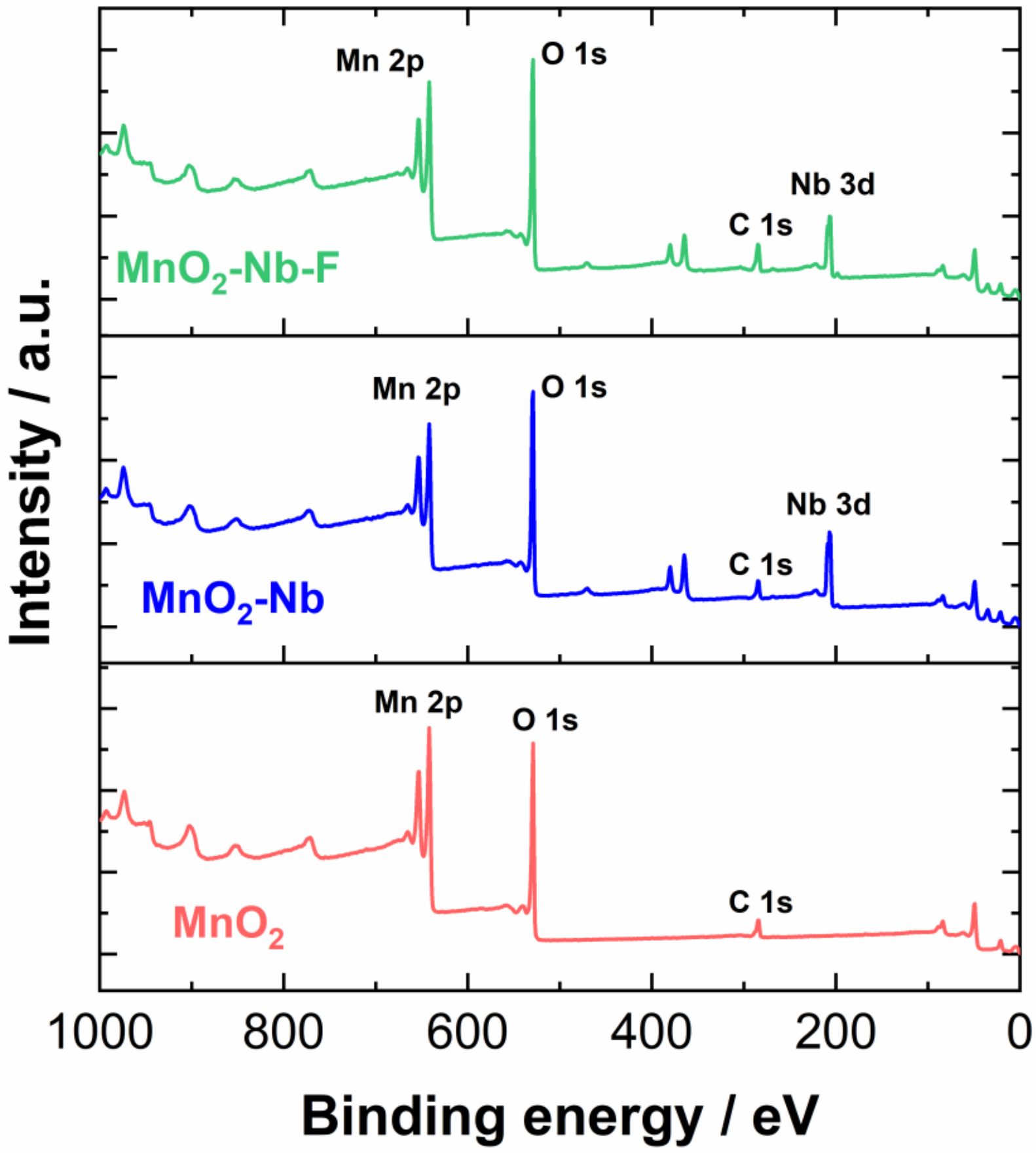

Figure S14. XPS survey spectra for MnO_2, MnO_2-Nb, and MnO_2-Nb-F.

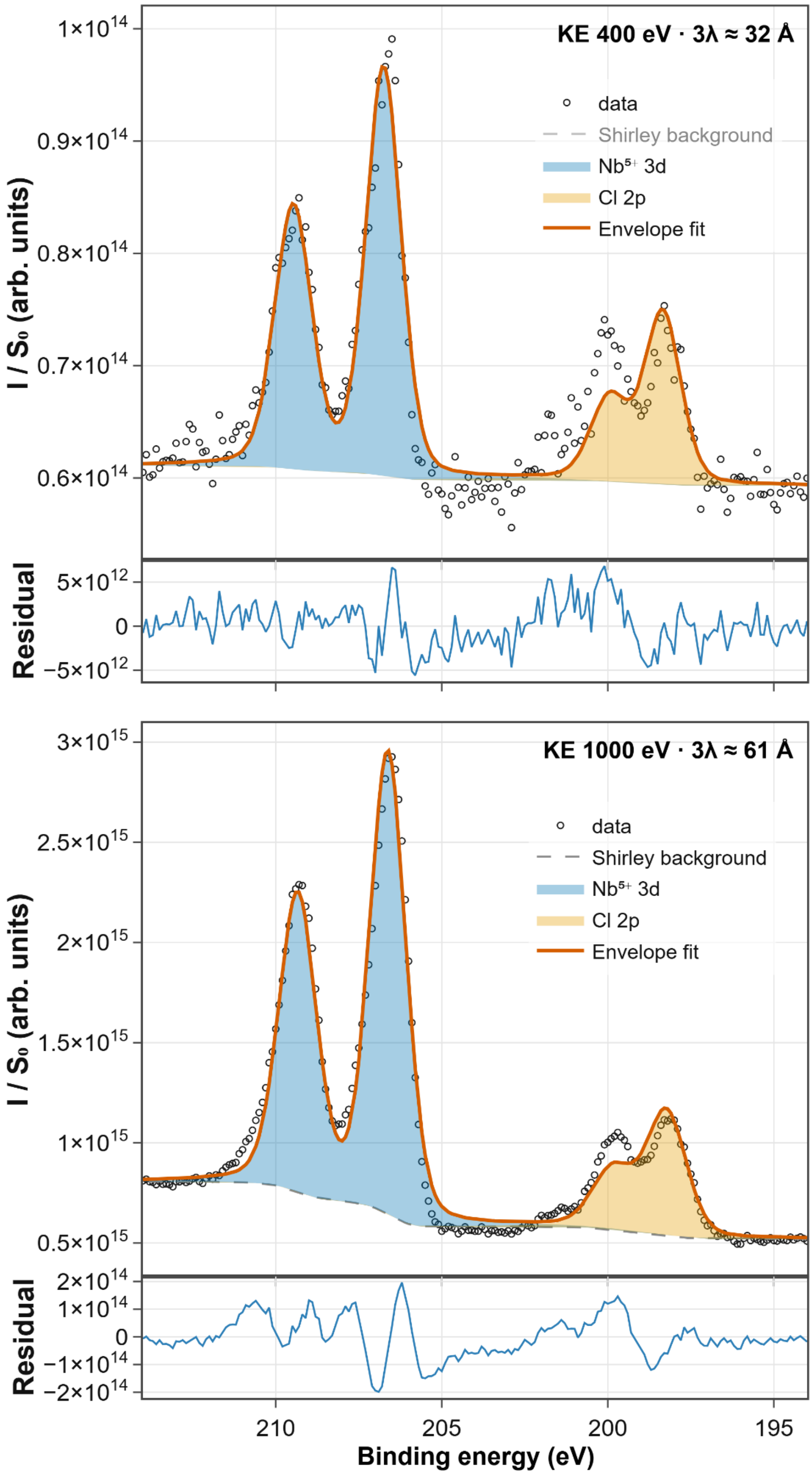

Figure S15. Depth-resolved Nb 3d (KE 400 and 1000 eV; 3λ ≈ 3.2 and 6.1 nm), sensitivity-normalized. A single Nb^{5+} (Nb_2O_5-like) 3d doublet at 206.9/209.7 eV at both depths, with no metallic/segregated-oxide (Nb_xO_y) shoulder near 205 eV.

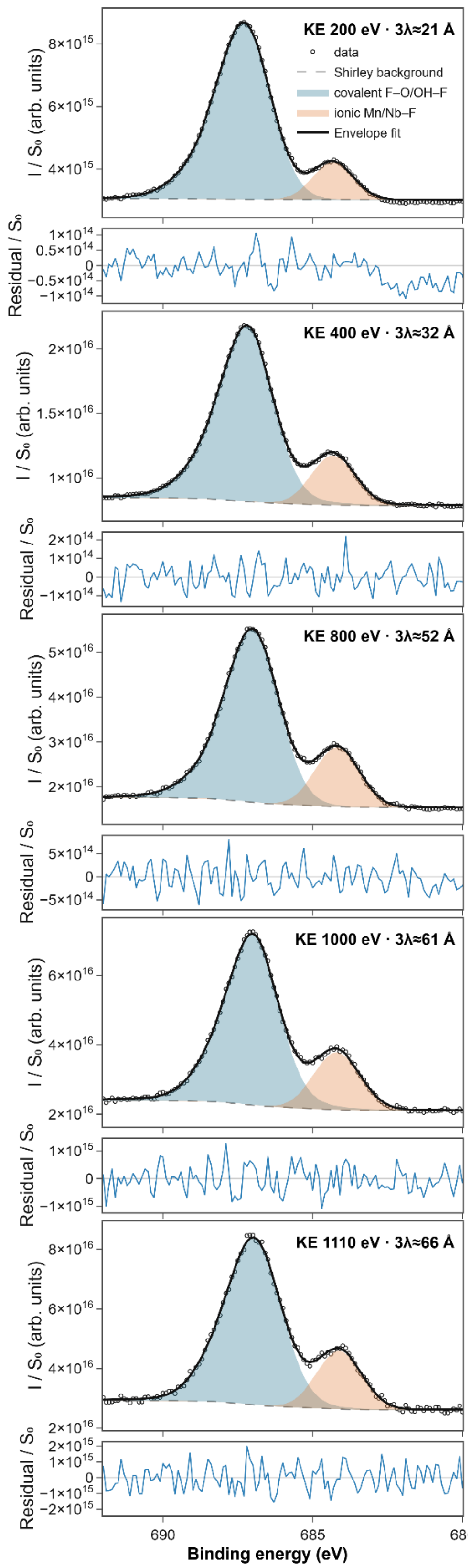

Figure S16. Depth-resolved F 1s (KE 200 → 1110 eV; 3λ ≈ 2.1 → 6.6 nm), sensitivity-normalized, with a global two-component fit and residuals. Covalent F–O/F–OH at 687.3 eV and ionic (lattice) Mn/Nb–F at 684.2 eV is present at every depth.

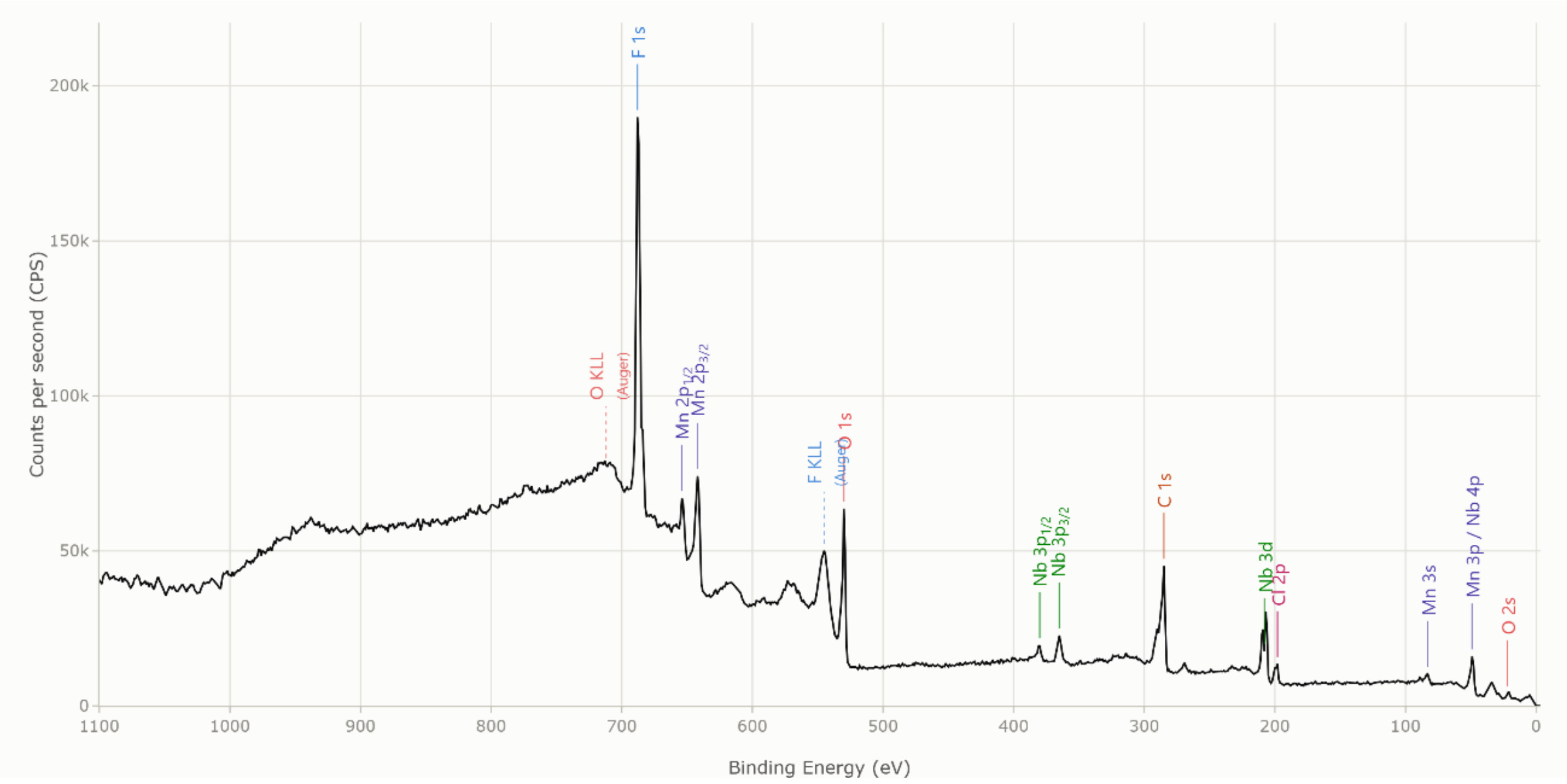

Figure S17. Synchrotron XPS survey with 1200 eV excitation energy. An F 1s photoemission line is observed at ≈ 685 eV, together with Mn 2p, O 1s and Nb 3d core levels, providing direct, unambiguous detection of fluorine.

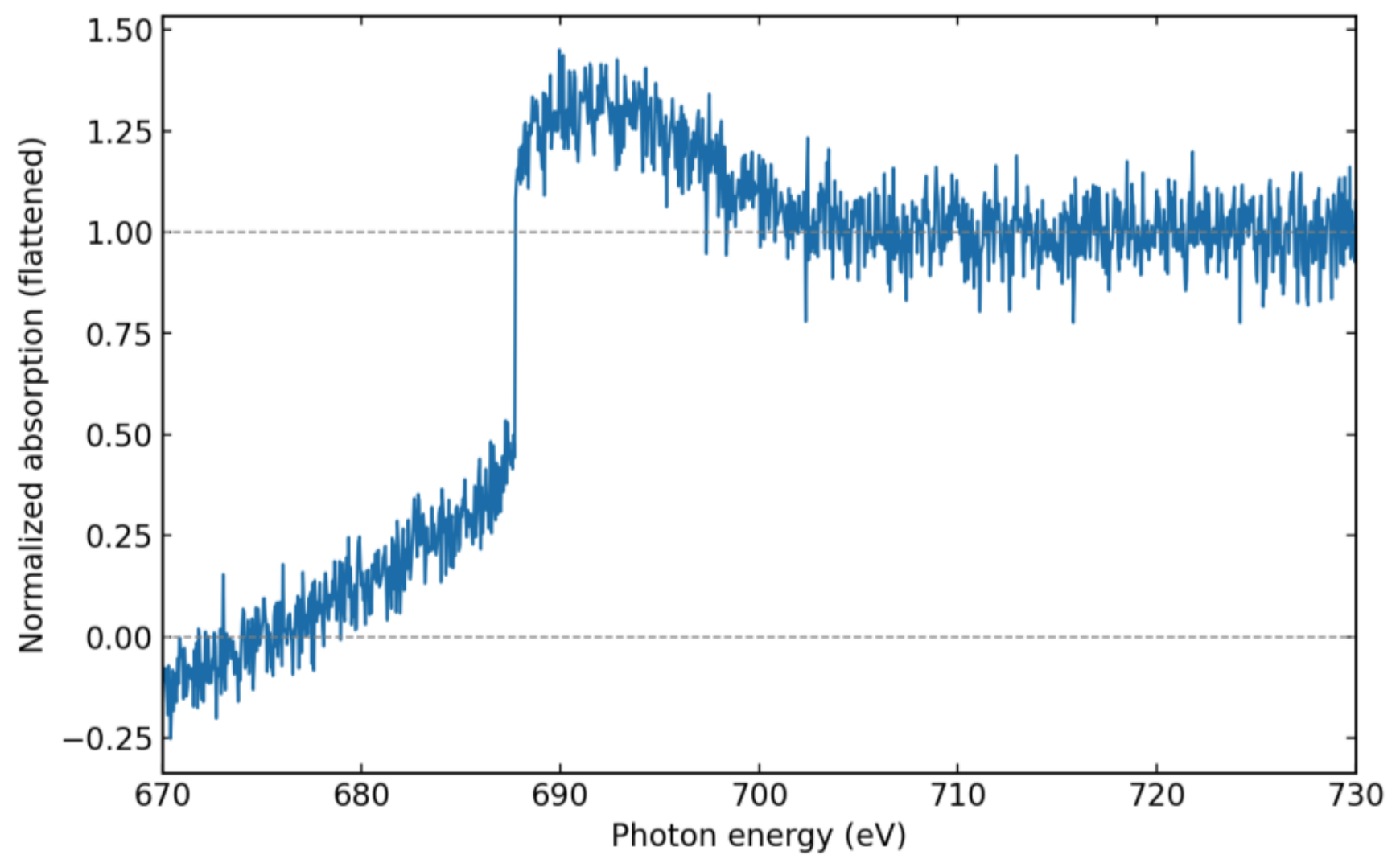

Figure S18. *F K-edge XANES, surface-sensitive Auger-electron yield (AEY, ≈ 1–2 nm). Clear F K-edge, onset ≈ 688 eV.*

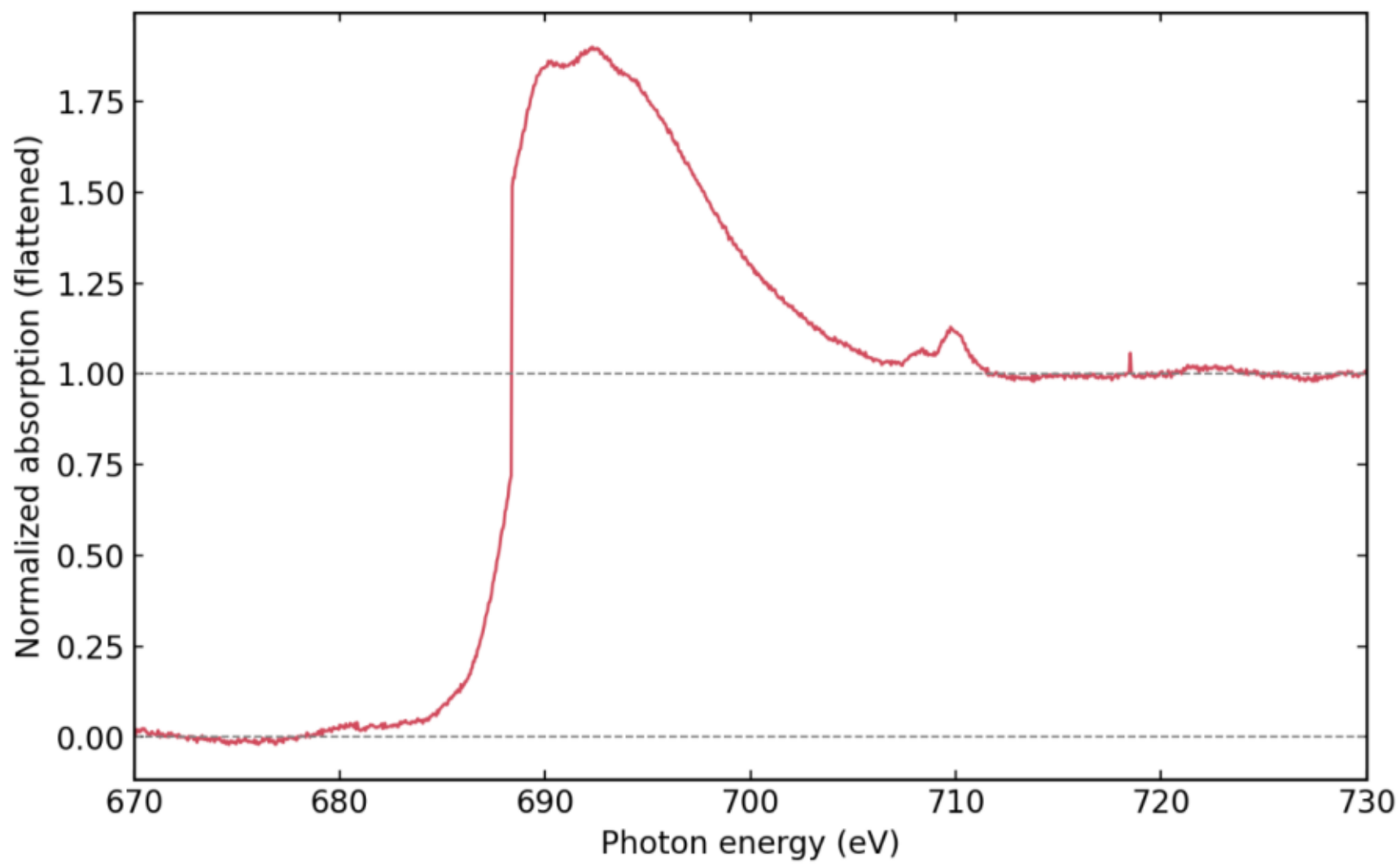

Figure S19. *F K-edge XANES, bulk-sensitive total-electron yield (TEY, ≈ 5–10 nm). The edge and metal-fluoride/fluoro-oxide white-line persist in the deeper-probing channel, confirming sub-surface fluorine.*

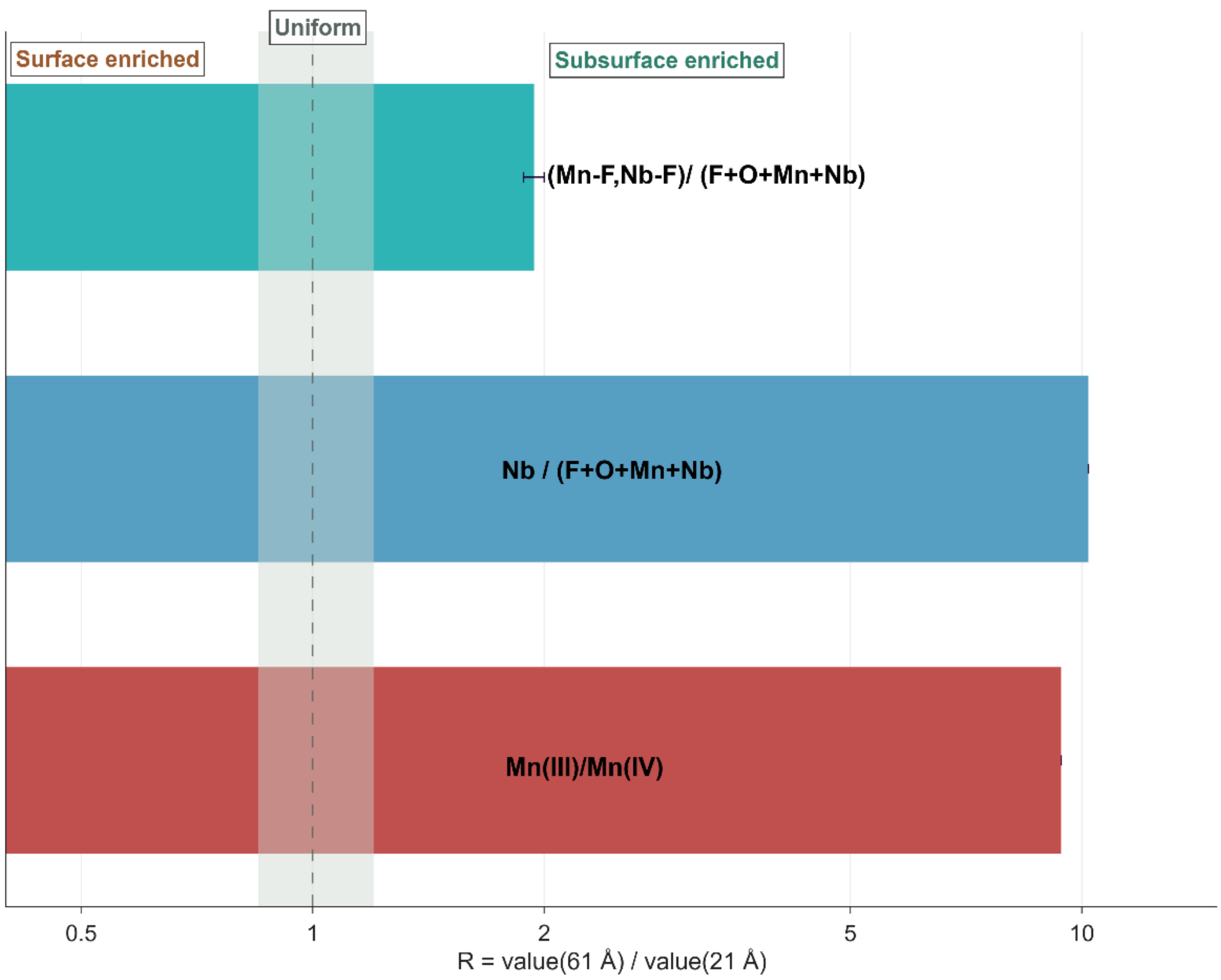

Figure S20. Depth-contrast factor R for the key sensitivity-normalized quantities. Ionic fluoride, Nb and Mn(III)/Mn(IV) all fall into the subsurface-enriched regime (R > 1); Nb and Mn(III)/Mn(IV) reach R ≈10.2 and 9.4.

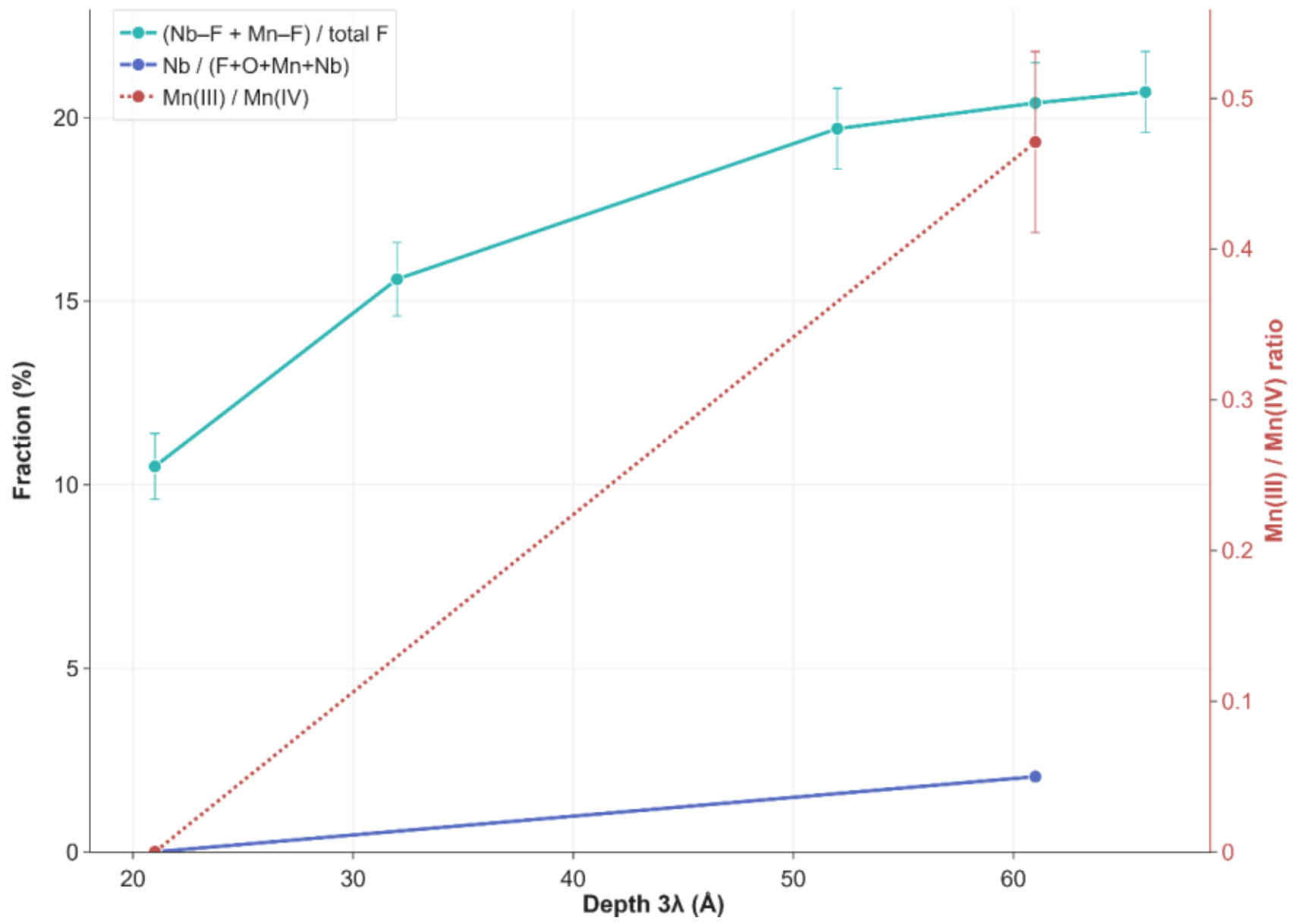

Figure S21. Transmission-independent fractions vs information depth: ionic (Nb–F + Mn–F)/total F rises 10.5 % → 20.7 %; Nb/(F+O+Mn+Nb) and the Mn(III)/Mn(IV) ratio increase monotonically toward the interior.

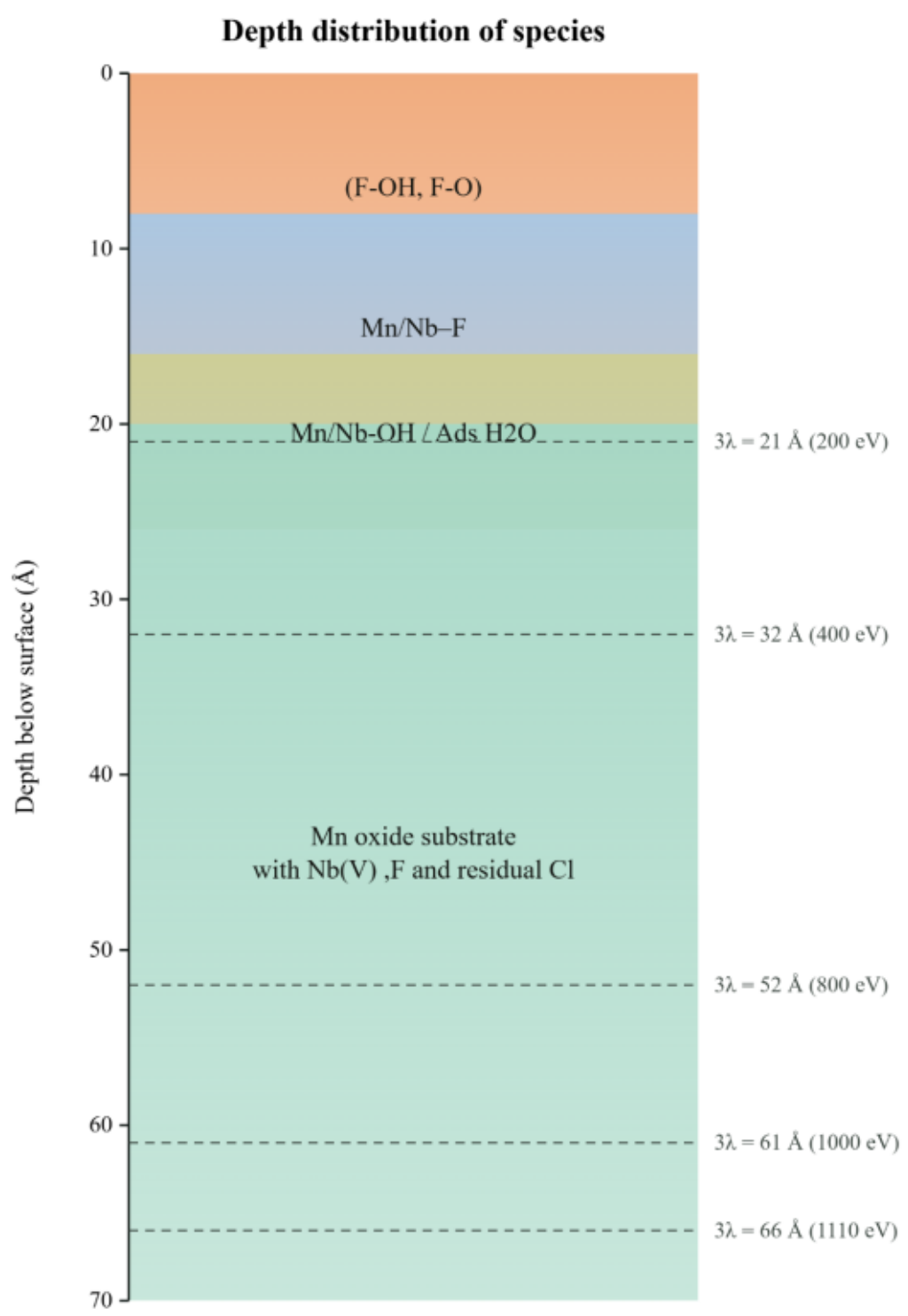

Figure S22. Depth model consistent with all core levels: a thin (~1–2.5 nm) hydroxide/adsorbate- and covalent-F-rich, chloride-bearing outer skin over an Mn-oxide interior carrying Nb(V), lattice F and residual Cl. Dashed lines mark the 3λ depths of the five kinetic energies.

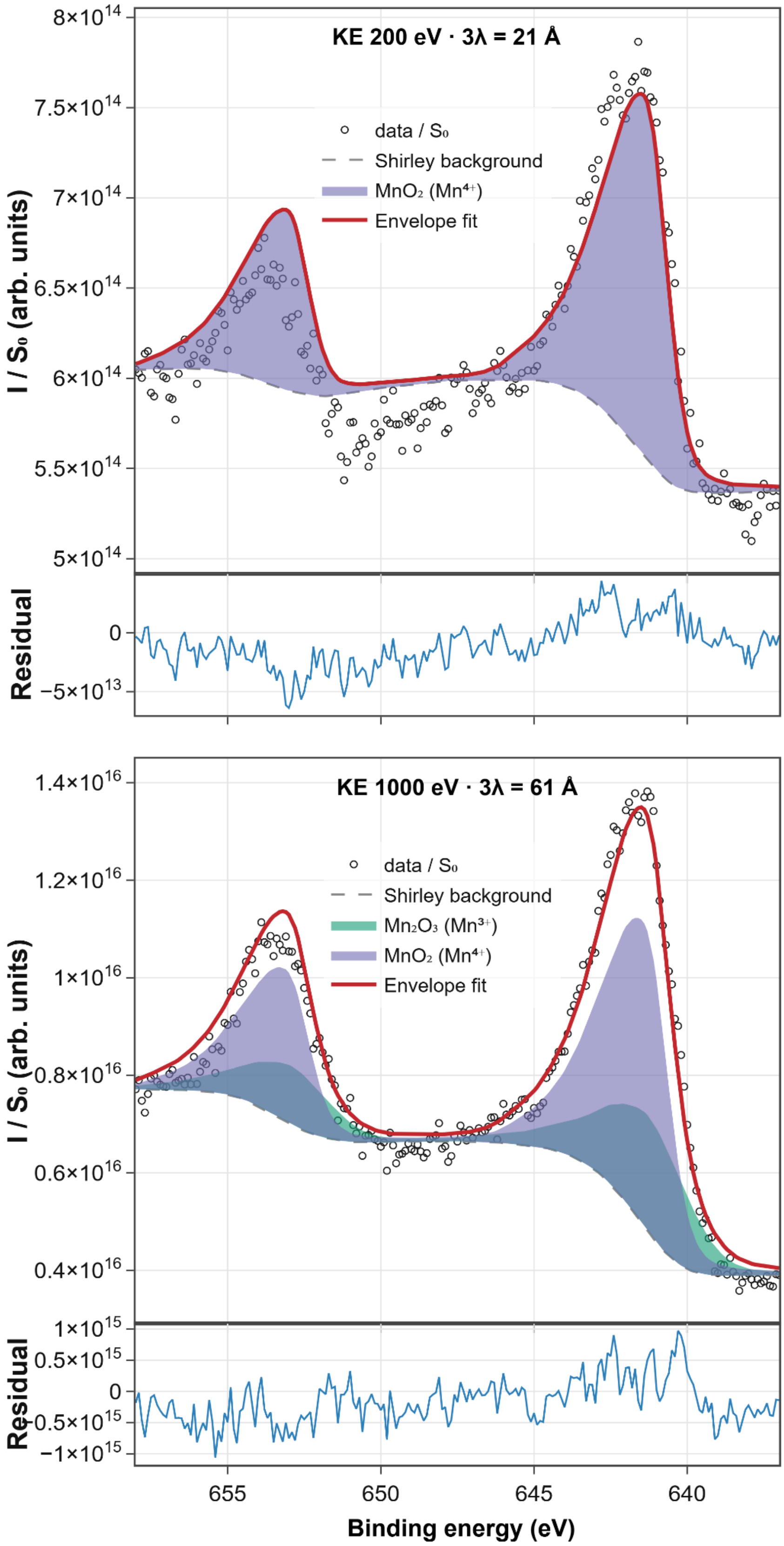

Figure S23. Depth-resolved Mn 2p. The Mn^{3+}/Mn^{4+} (Mn_2O_3/MnO_2) ratio increases with depth, the doped interior is more reduced, matching the operando Mn K-edge XANES conclusion.

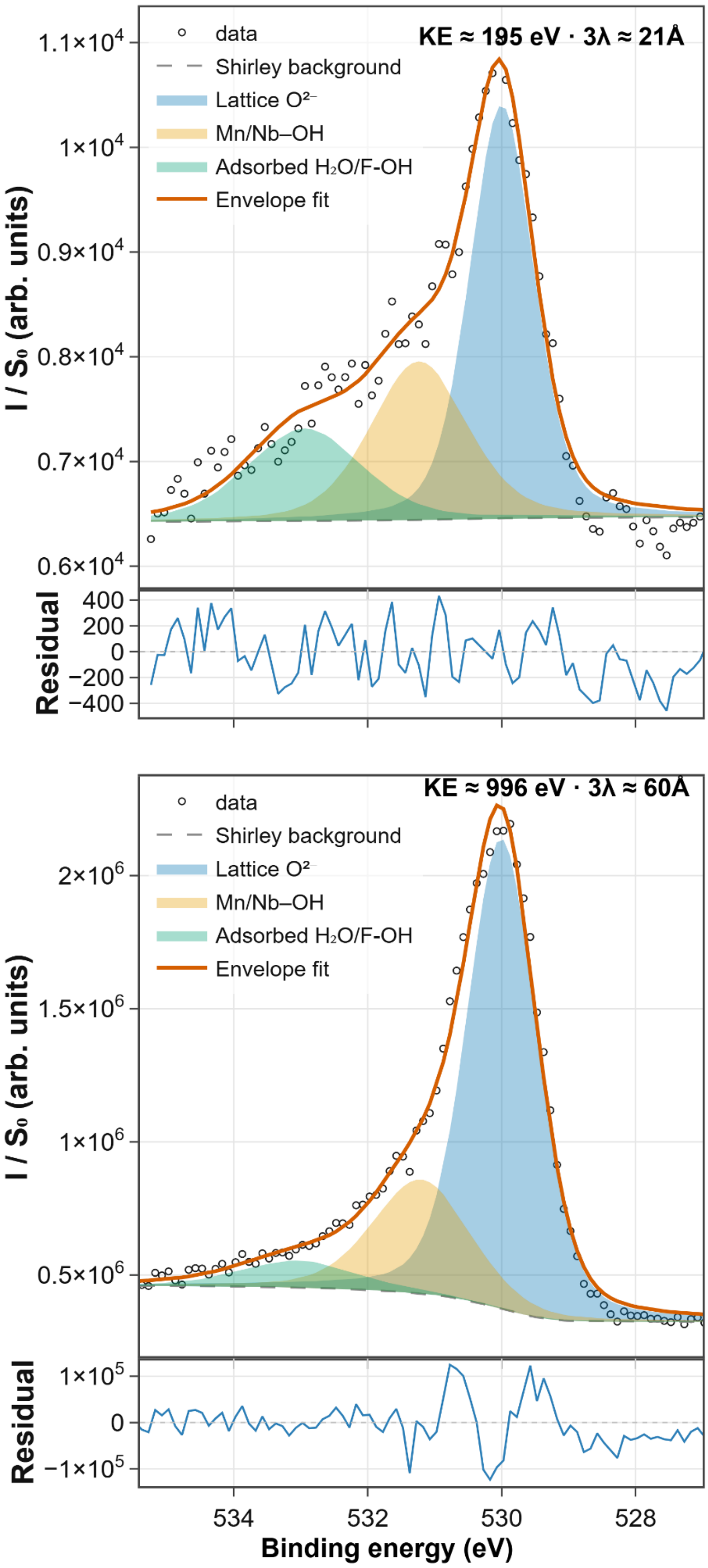

Figure S24. Depth-resolved O 1s (lattice O^{2-} ≈ 530 eV, Mn/Nb–OH ≈ 531 eV, adsorbed H_2O/F–OH ≈ 533 eV). The lattice-O^{2-} fraction grows with depth while surface hydroxide/adsorbate components fall. a hydroxylated surface skin over a more oxidic, doped interior.

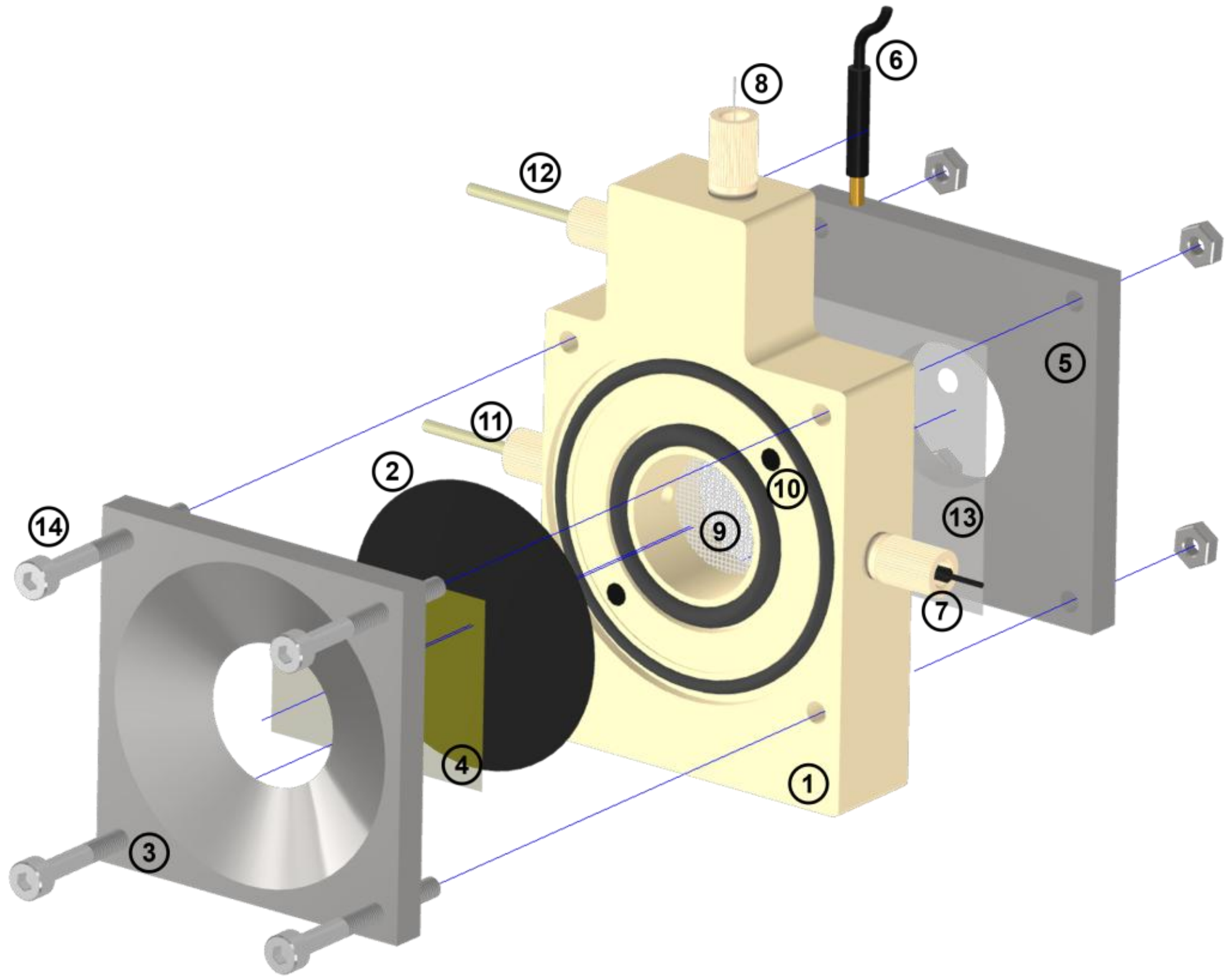

Figure S25. Schematic representation of the spectroelectrochemical cell used for the X-ray absorption experiments. 1) Cell body machined out of PEEK plastic. 2) Carbon felt working electrode. 3) Aluminium lid. 4) Kapton tape is attached to the lid to prevent leaks from the porous carbon felt. 5) Back plate made of stainless steel, it is used to make the connection between the potentiostat (6) and the working electrode, using a glassy carbon (10) and spring-loaded pins (not shown). 7) Reference electrode, sealed with a silicon ring and the screwed fitting. 8) Pt wire used for connecting the counter electrode to the potentiostat. 9) A Pt mesh is used as counter electrode, welded to the Pt wire. 11) Electrolyte inlet. 12) Electrolyte outlet. 13) PET is used to seal the back side of the cell, which allows to observe any bubble formation. Note that two holes are needed for the spring-loaded pins to contact with the steel plate. 14) M2.5 screws are used to close the cell tightly. The technical drawings can be found in the Edmond data repository. [3]

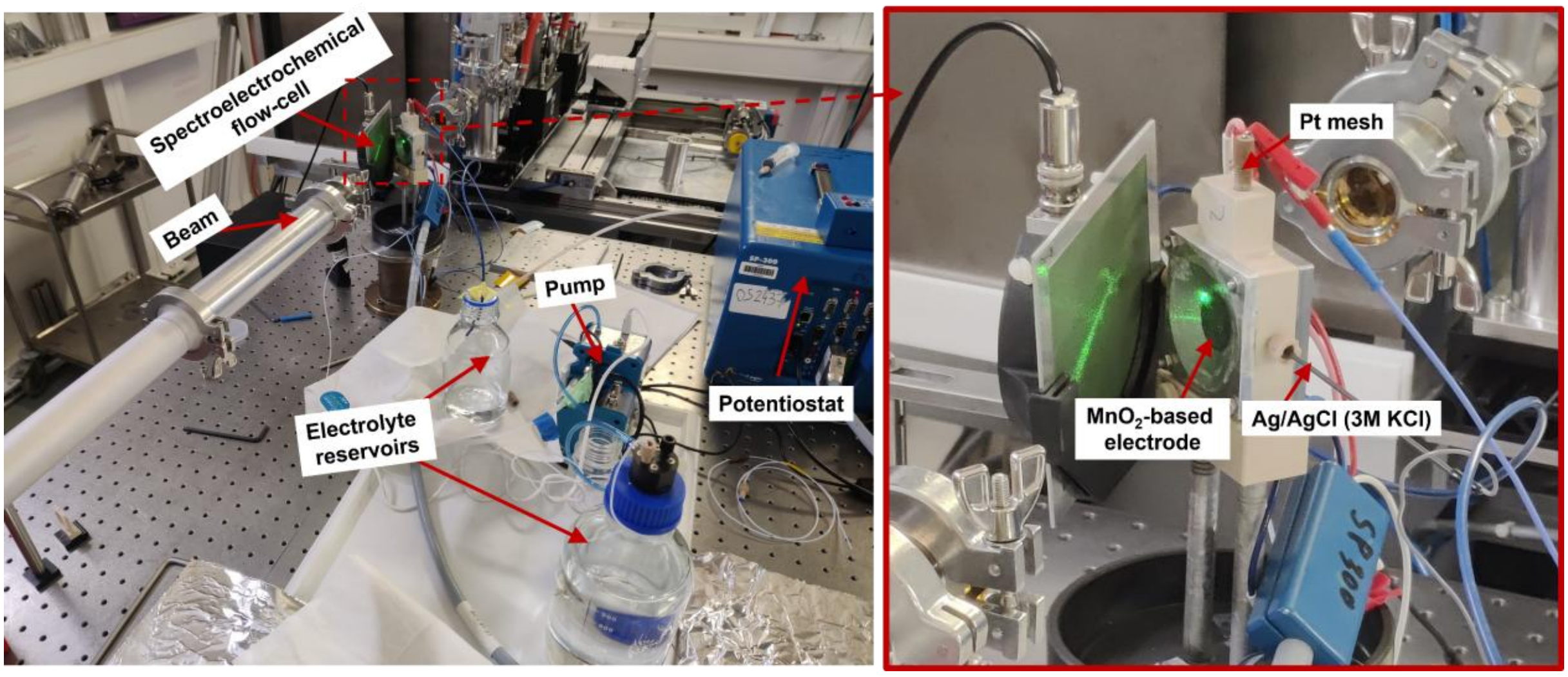

Figure S26. Operando spectroelectrochemical flow-cell setup used for XAS measurements. (a) Overview of the experimental configuration showing the flow cell aligned with the synchrotron beam, electrolyte reservoirs, pump, and potentiostat. (b) Enlarged view of the spectroelectrochemical flow-cell with the MnO_2-based working electrode, Pt mesh counter electrode, and Ag/AgCl (3.4 M KCl) reference electrode.

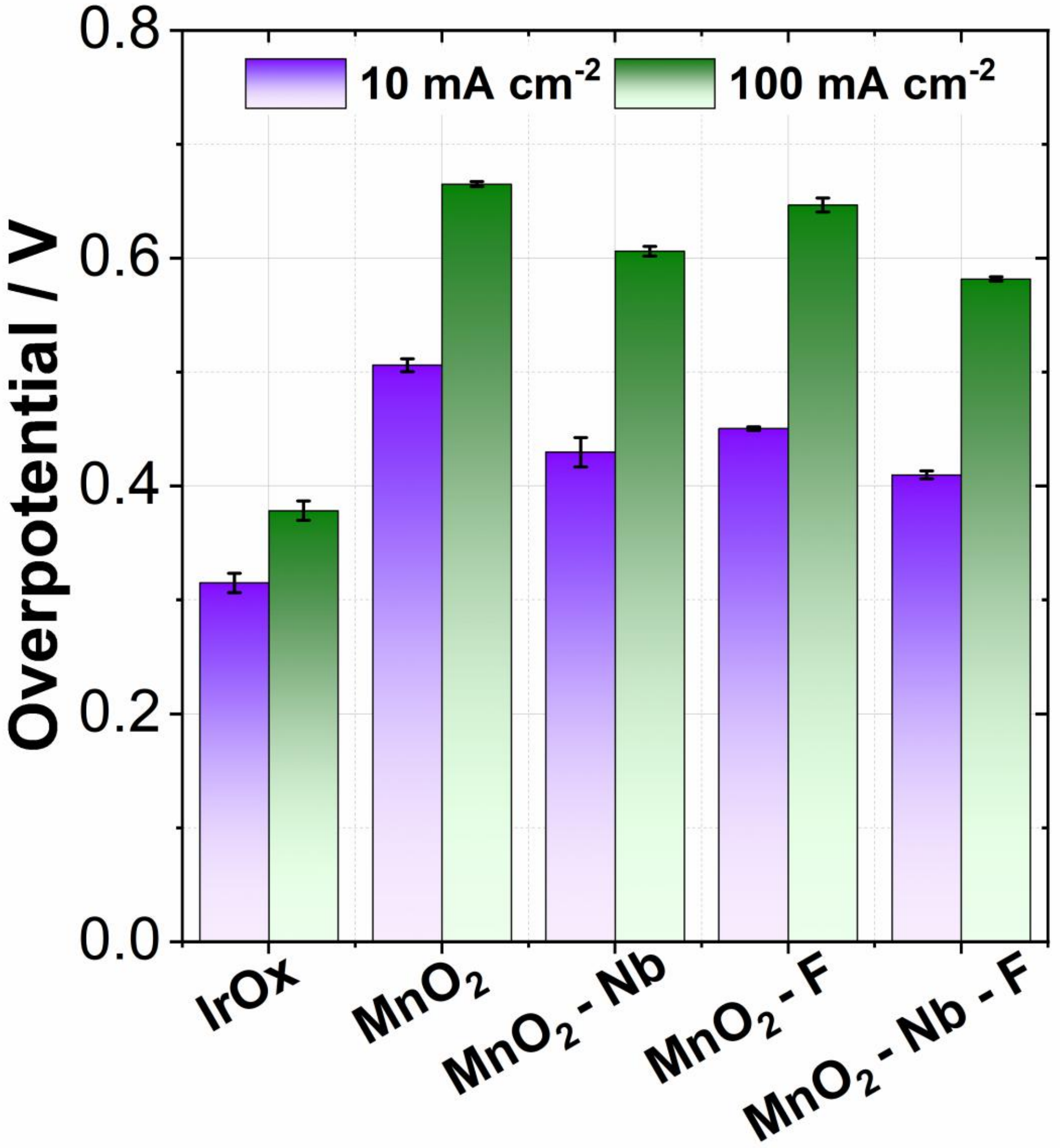

Figure S27. Overpotentials at 10 mA cm^{-2} and 100 mA cm^{-2} for the four catalyst and IrOx reference in 0.5 M H_2SO_4.

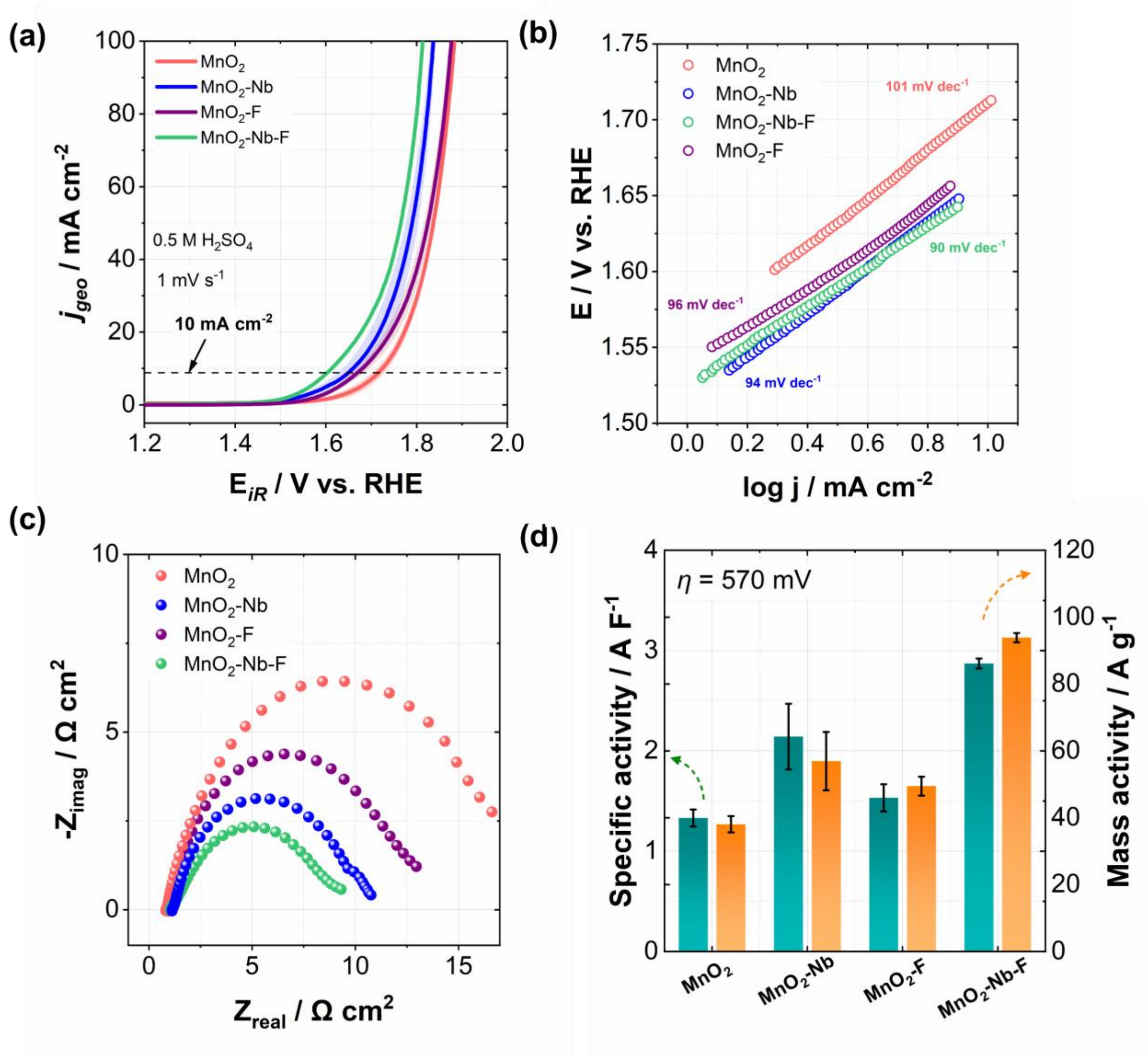

Figure S28. Electrochemical characterization benchmarking of the four catalyst in 0.5 M H_2SO_4. (a) *iR*-corrected linear sweep voltammograms (1 mV s^{-1}), Shaded regions represent standard deviation across replicate measurements. (b) Corresponding Tafel plots. (c) Nyquist plots from electrochemical impedance spectroscopy at 1.75 V vs RHE. (d) Specific and mass activity at $\eta = 570$ mV. Error bars represent standard deviation across replicate measurements.

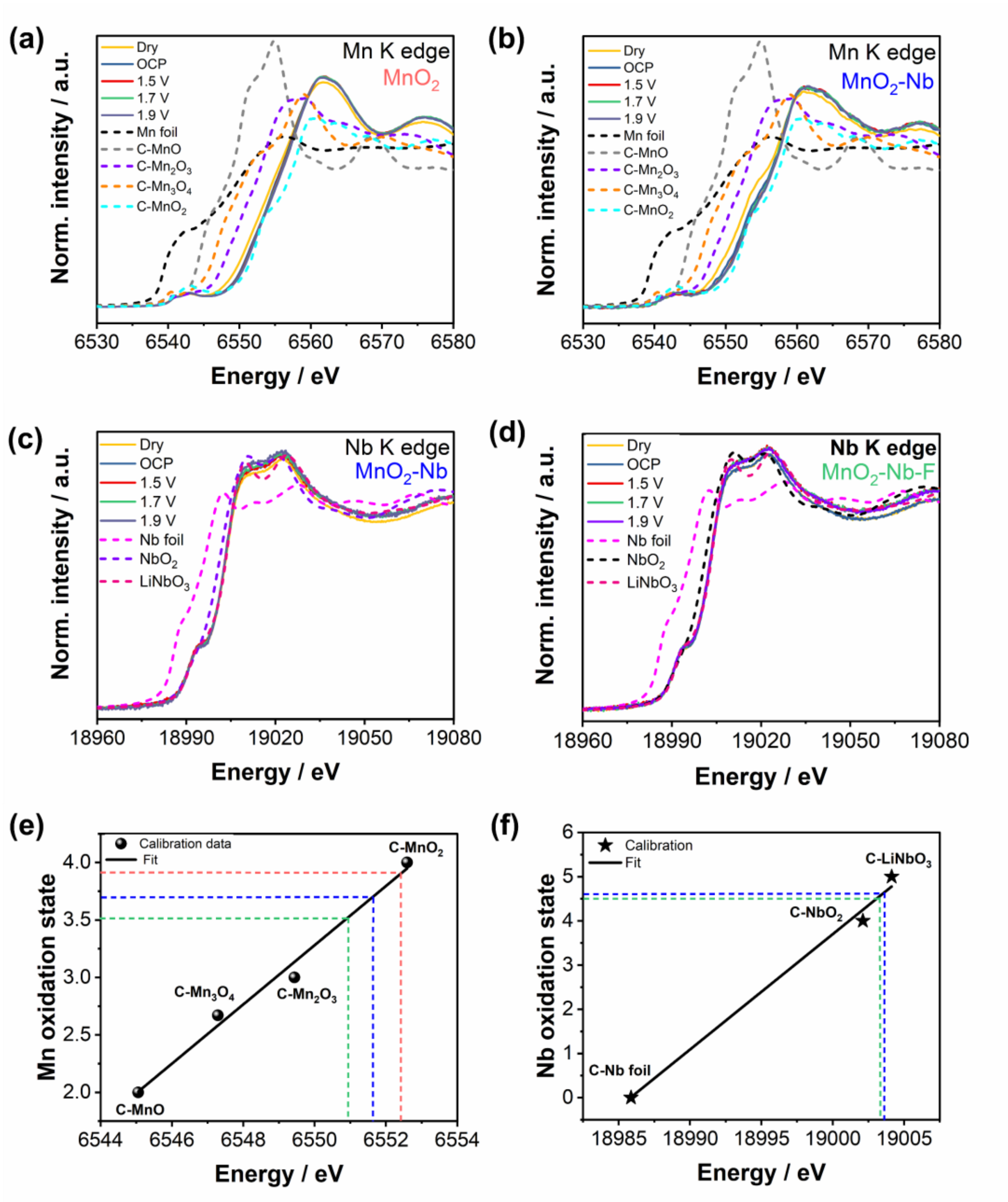

Figure S29. Operando XANES analysis of Mn and Nb electronic structure evolution. Mn K-edge XANES spectra of (a) MnO_2 and (b) MnO_2-Nb under dry, electrolyte-contacted (no applied bias), and anodic polarization conditions (1.5-1.9 V vs. RHE), compared with Mn reference compounds. Nb K-edge XANES spectra of (c) MnO_2-Nb and (d) MnO_2-Nb-F collected under dry and operando conditions. (e) Calibration curve for Mn oxidation state determination derived from Mn K-edge absorption energies (E_o, first derivative maximum) of references (MnO, Mn_3O_4, Mn_2O_3, MnO_2), used to calculate the average Mn oxidation states of MnO_2, MnO_2-Nb, and MnO_2-Nb-F. (f) Calibration curve for Nb oxidation state determination based on Nb K-edge absorption energies of Nb foil,NbO_2, and $LiNbO_3$ references, used to extract the average Nb oxidation states of MnO_2-Nb and MnO_2-Nb-F electrodes.

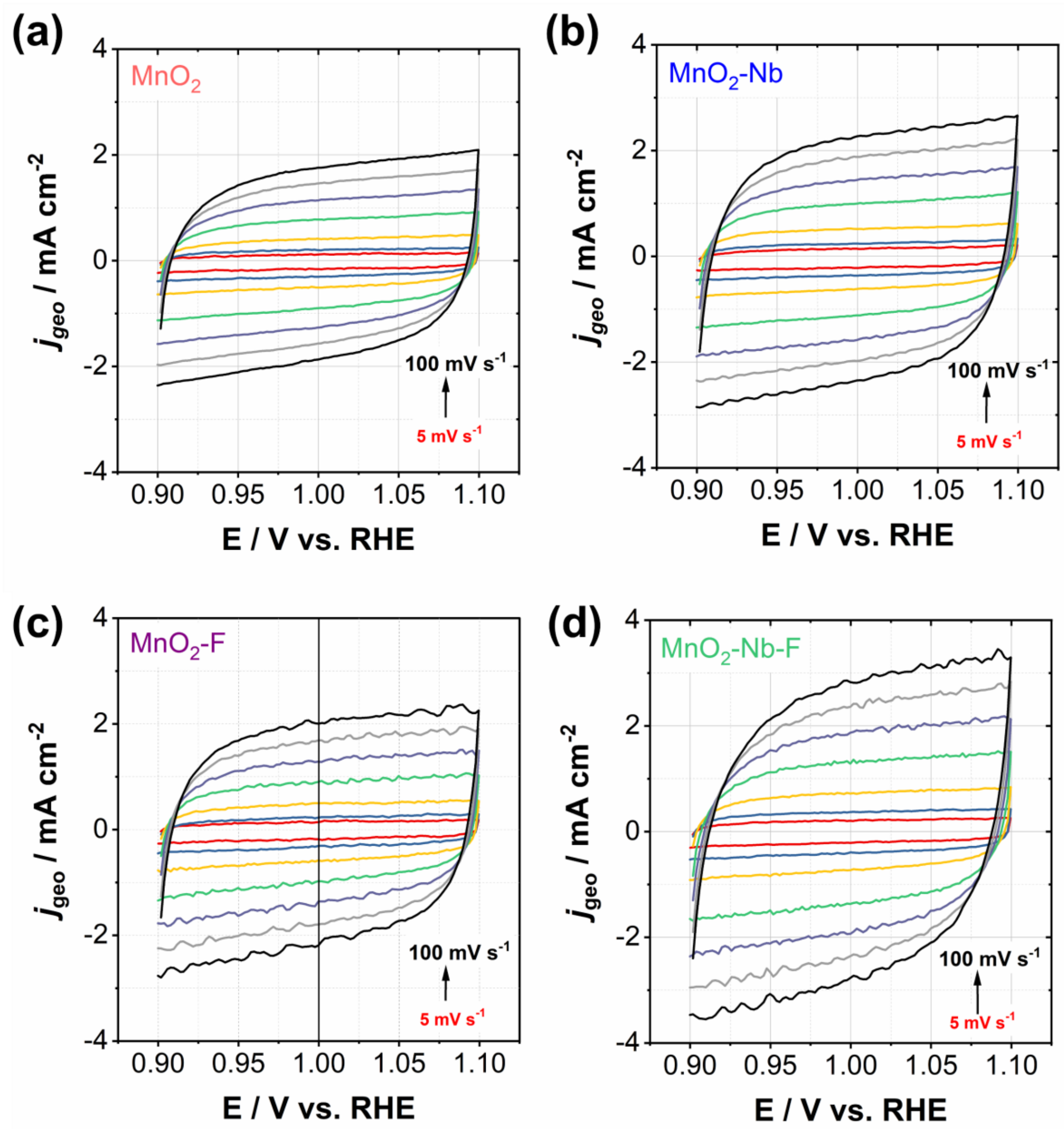

Figure S30. Cyclic voltammograms recorded in the non-faradaic region for calculating the double-layer capacitance of (a) MnO_2, (b) MnO_2-Nb, (c) MnO_2-F and (d) MnO_2-Nb-F.

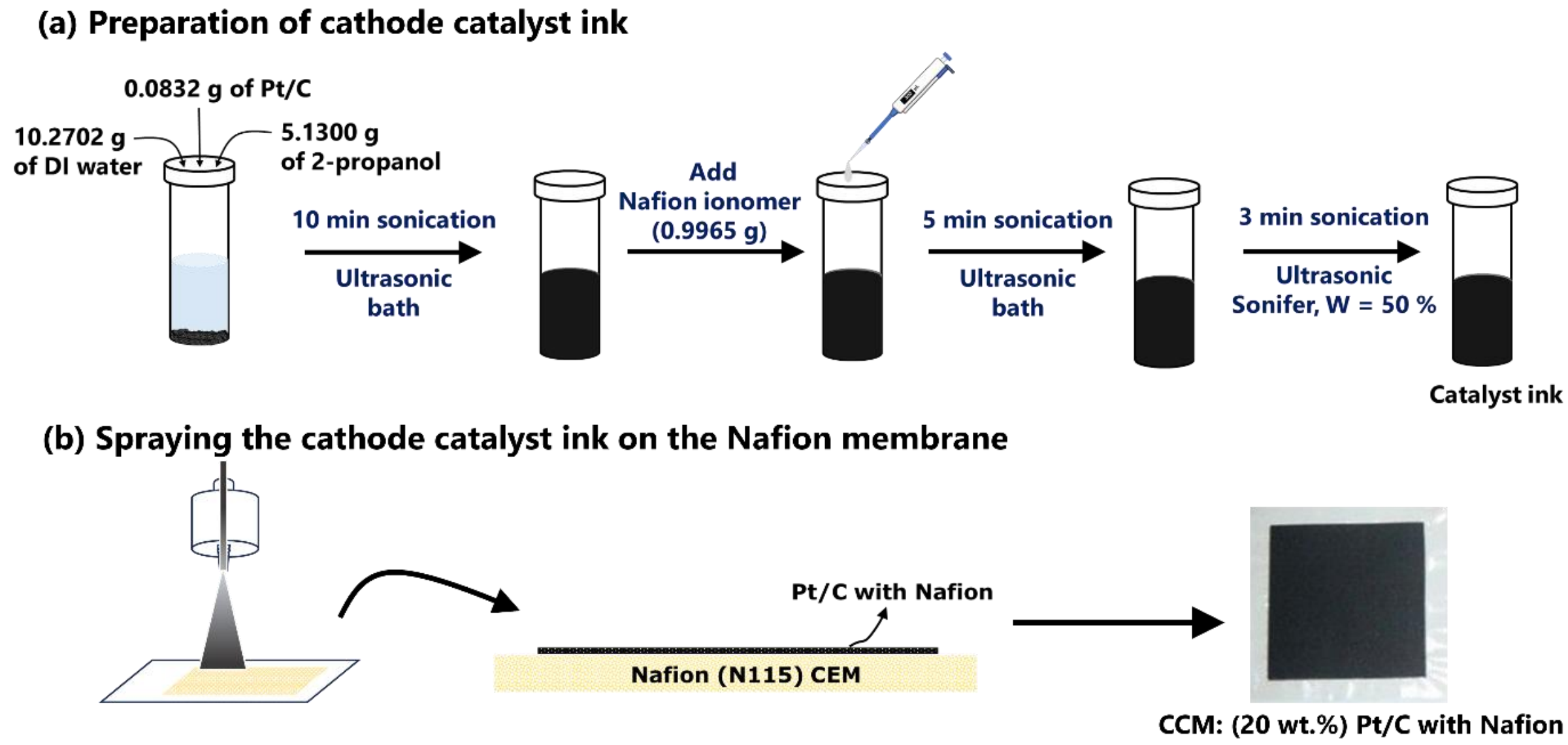

Figure S31. Schematic illustration of the preparation of the Pt/C cathode catalyst ink and its deposition onto a Nafion (N115) membrane.

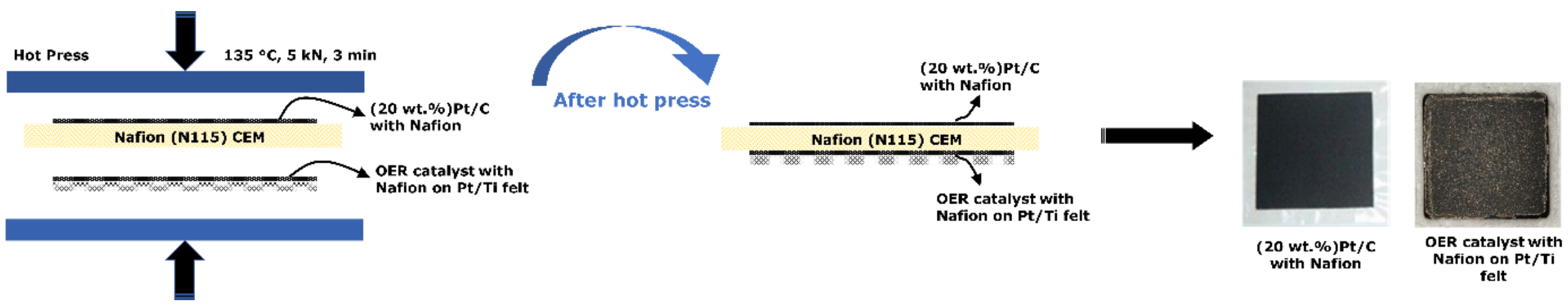

Figure S32. Schematic of membrane-electrode assembly (MEA) fabrication by hot pressing.

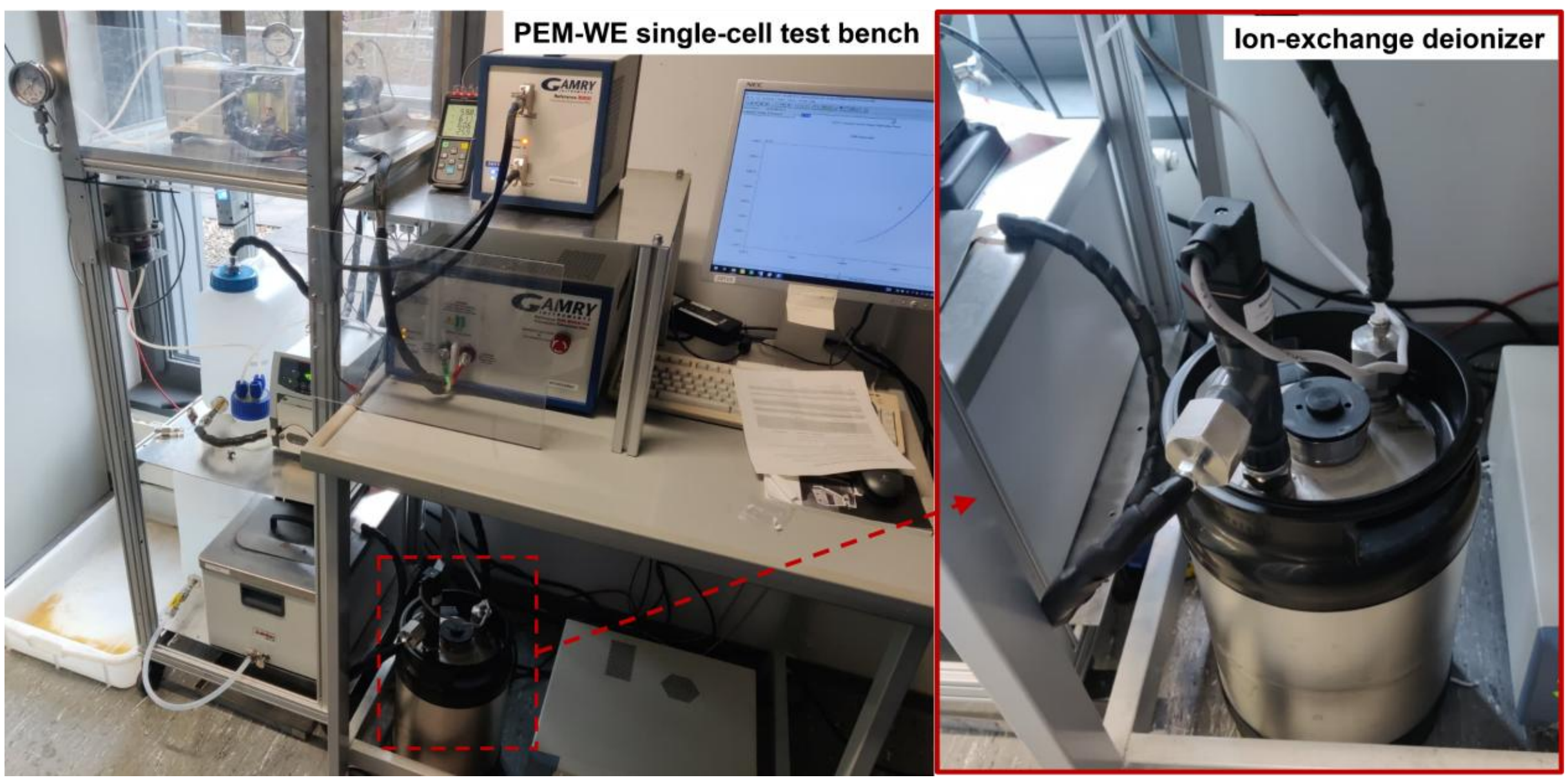

Figure S33. PEM-WE single-cell test bench with mixed-bed ion-exchange deionizer (inset).

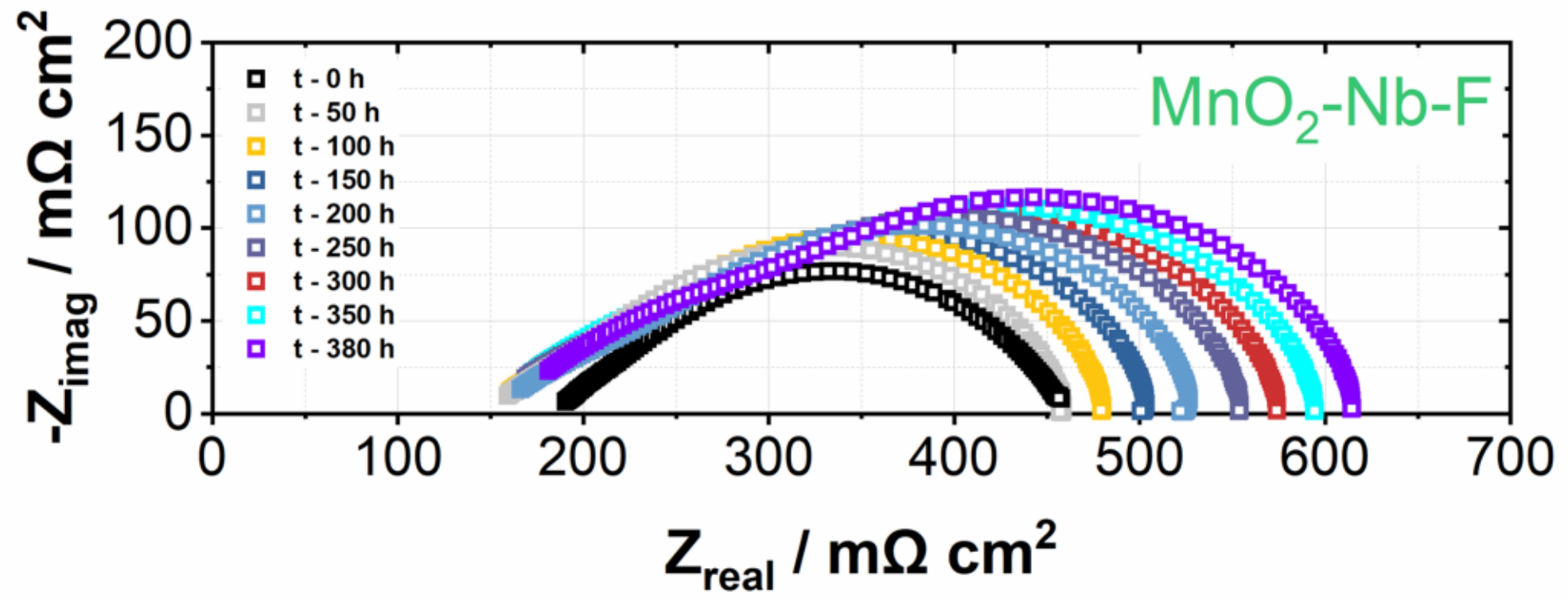

Figure S34. Nyquist plots of the MnO_2-Nb-F anode recorded at different operation times during constant-current PEM electrolyzer operation at 60 °C.

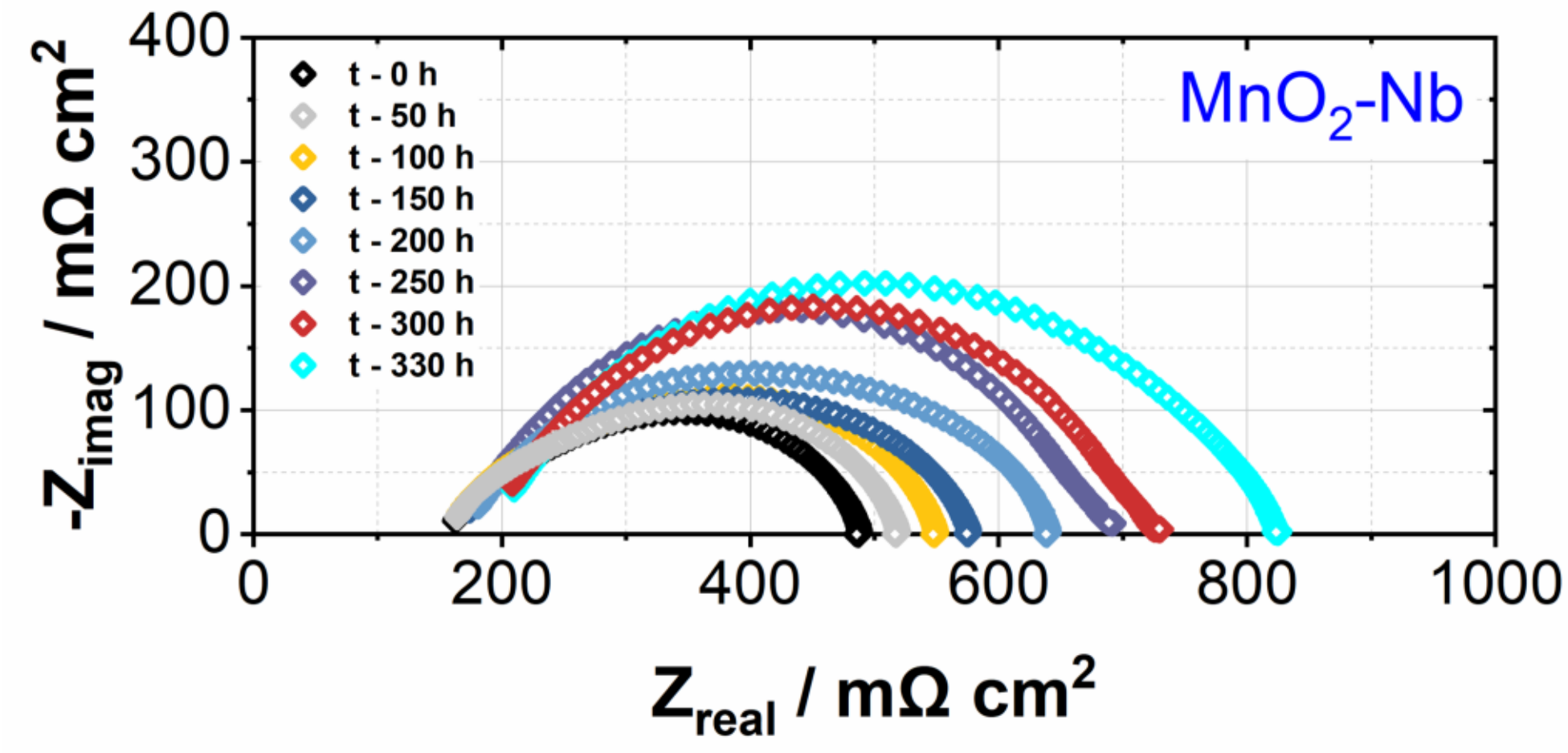

Figure S35. Nyquist plots of the MnO_2-Nb anode recorded at different operation times during constant-current PEM electrolyzer operation at 60 °C.

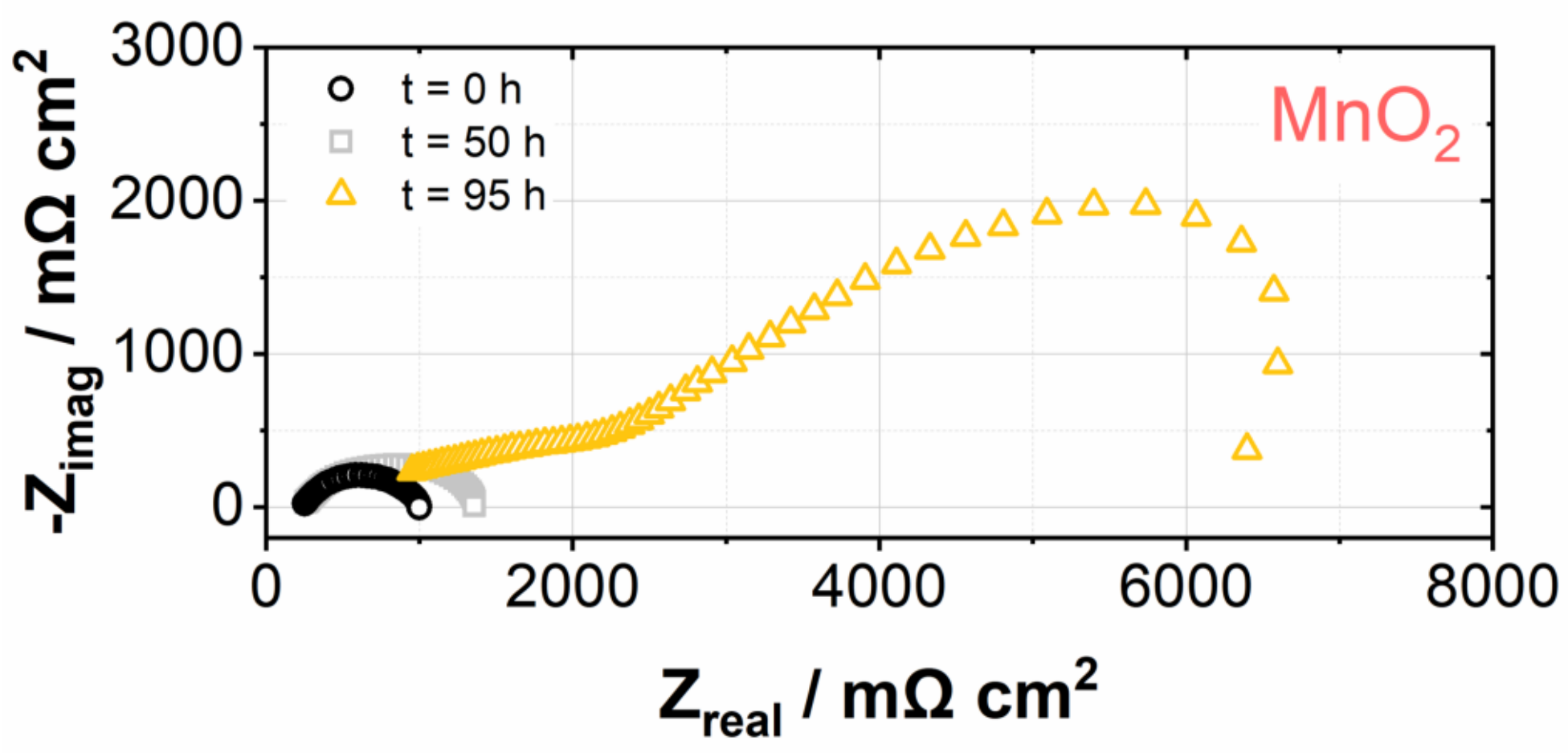

Figure S36. Nyquist plots of the MnO_2 anode recorded at different operation times during constant-current PEM electrolyzer operation at 60 °C.

Table S3. Comparison of activity and stability for a variety of OER catalysts in acidic media.

#	Catalyst	Electrolyte	Overpotential @10 mA cm^{-2}	Stability	Mass Activity	Ref.
**1**	**MnO_2-Nb-F**	**0.5 M H_2SO_4**	**410 ± 4 mV**	**250 h @ 10 mA cm^{-2}(a); 380 h @ 400 mA cm^{-2}(b)**	**93.9 A g^{-1}**	**This work**
2	$Mn_{7.5}O_{10}Br_3$	0.5 M H_2SO_4	295 ± 5 mV	500 h @ 10 mA cm^{-2}(a); 300 h @ 100 mA cm^{-2}(b)	n.r.	[4]
3	γ/β-MnO_2	1 M H_2SO_4	522 mV	400 h @ 100 mA cm^{-2}(a)	n.r.	[5]
4	γ-MnO_2	1 M H_2SO_4	428 ± 5 mV	8000 h @ 10 mA cm^{-2}(a); 12 h @ 100 mA cm^{-2}(b)	n.r.	[6]
5	γ-MnO_2 (94%)	1 M H_2SO_4	445 ± 2 mV	1000 h @ 200 mA cm^{-2}(b)	n.r.	[7]
6	$Ni_{0.5}Mn_{0.5}Sb_{1.7}O_y$	1 M H_2SO_4	672 ± 9 mV	170 h @ 10 mA cm^{-2}(a)	n.r.	[8]
7	LaMn@Co-ZIF	0.1 M $HClO_4$	353 ± 11 mV	360 h @ 10 mA cm^{-2}(a); 100 h @ 210 mA cm^{-2}(b)	n.r.	[9]
8	$(Mn_{0.8}Nb_{0.2})O_2$:10F	1 M H_2SO_4	680 mV	23 h @ 1.9 V_{RHE}(a)	29 A g^{-1}	[10]
9	MnCoOx	0.5 M H_2SO_4	275 mV	300 h @ 100 mA cm^{-2}(a)	n.r.	[11]
10	MnO_2@Er_2O_3	0.5 M H_2SO_4	342 ± 5 mV	30 h @ 50 mA cm^{-2}(a)	n.r.	[12]
11	Co_3O_4	0.5 M H_2SO_4	370 mV	86.8 h @ 10 mA cm^{-2}(a)	n.r.	[13]
12	$Co_xFe_{1-x}O_y$	0.5 M H_2SO_4	650 mV	50 h @ 10 mA cm^{-2}(a)	n.r.	[14]
13	Co_2MnO_4	1.5 M H_3PO_4	298 mV	1500 h @ 200 mA cm^{-2}(a)	n.r.	[15]
14	$Nb_{0.1}Mn_{0.1}Ru_{0.8}O_2$	0.5 M H_2SO_4	209 mV	400 h @ 200 mA cm^{-2}(a); 1000 h @ 500 mA cm^{-2}(b)	127 A g^{-1}	[16]
15	$Nb_{0.1}Ru_{0.9}O_2$	0.5 M H_2SO_4	204 mV	360 h @ 200 mA cm^{-2}(a); 100 h @ 300 mA cm^{-2}(b)	150 A g^{-1}	[17]
16	C-RuO_2	0.5 M H_2SO_4	296 mV	19.5 h @ 10 mA cm^{-2}(a)	5.4 A g^{-1}	[18]
17	C-IrO_2	0.5 M H_2SO_4	321 mV	23.5 h @ 10 mA cm^{-2}(a)	6.6 A g^{-1}	[18]
18	$Ru_{0.5}Ir_{0.5}O_2$	0.5 M H_2SO_4	151 mV	618 h @ 10 mA cm^{-2}(a); 255 h @ 200 mA cm^{-2}(b)	730 A g^{-1}	[18]

19	$Ta_{0.1}Tm_{0.1}Ir_{0.8}O_{2-\delta}$	0.5 M H_2SO_4	198 mV	500 h @ 10 mA cm^{-2}(a); 500 h @ 1500 mA cm^{-2}(b)	3.5 A g^{-1}	[19]
20	Sr-$IrMnO_2$	0.5 M H_2SO_4	236 mV	400 h @ 10 mA cm^{-2}(a); 500 h @ 1500 mA cm^{-2}(b)	1932 A g^{-1}	[20]
21	IrRuCoNiMo	0.5 M H_2SO_4	237 mV	24 h @ 10 mA cm^{-2}(a); 500 h @ 1000 mA cm^{-2}(b)	261 A g^{-1}	[21]
22	TS–Ir/MnO_2	0.5 M $HClO_4$	198 mV	200 h @ 100 mA cm^{-2}(a); 200 h @ 500 mA cm^{-2}(b)	1025 A g^{-1}	[22]

n.r. = not reported.

a = Stability experiment in a half-cell (three-electrode setup)

b = Stability experiment in a full cell (PEM-WE setup)

References:

[1] M. C. Biesinger, B. P. Payne, A. P. Grosvenor, L. W. Lau, A. R. Gerson, R. S. Smart, *Appl. Surf. Sci.* **2011**, *257*, 2717.

[2] B. Ravel, M. Newville, *J. Synchrotron Radiat.* **2005**, *12*, 537.

[3] O. Rüdiger, M. Gil Sepulcre, G. Huang, C. Laurich, S. DeBeer, X-ray Spectroelectrochemical Flow Cell, Edmond, 2026. https://doi.org/10.17617/3.T7MHLT.

[4] S. Pan, H. Li, D. Liu, R. Huang, X. Pan, D. Ren, J. Li, M. Shakouri, Q. Zhang, M. Wang et al., *Nat. Commun.* **2022**, *13*, 2294.

[5] L. Liu, S. Kong, Y. Sun, Q. Hu, P. Zhang, A. Li, K. Adachi, D. Hashizume, R. Nakamura, T. Liu et al., *ACS Sustainable Chem. Eng.* **2024**, *12*, 17613.

[6] A. Li, H. Ooka, N. Bonnet, T. Hayashi, Y. Sun, Q. Jiang, C. Li, H. Han, R. Nakamura, *Angew. Chem., Int. Ed. Engl.* **2019**, *58*, 5054.

[7] S. Kong, A. Li, J. Long, K. Adachi, D. Hashizume, Q. Jiang, K. Fushimi, H. Ooka, J. Xiao, R. Nakamura, *Nat. Catal.* **2024**, *7*, 252.

[8] I. A. Moreno-Hernandez, C. A. MacFarland, C. G. Read, K. M. Papadantonakis, B. S. Brunschwig, N. S. Lewis, *Energy Environ. Sci.* **2017**, *10*, 2103.

[9] L. Chong, G. Gao, J. Wen, H. Li, H. Xu, Z. Green, J. D. Sugar, A. J. Kropf, W. Xu, X.-M. Lin et al., *Science* **2023**, *380*, 609.

[10] S. D. Ghadge, O. I. Velikokhatnyi, M. K. Datta, P. M. Shanthi, S. Tan, P. N. Kumta, *ACS Appl. Energy Mater.* **2020**, *3*, 541.

[11] J. Zhang, A. Raza, Y. Zhao, S. Guo, Z. U. D. Babar, L. Xu, C. Cao, G. Li, *J. Mater. Chem. A* **2023**, *11*, 25345.

[12] S. Pan, S. Wang, Y. Wang, H. Li, D. Zhao, Y. Fu, H. Liu, J. Kou, N. Li, D. Wang, *J. Mater. Chem. A* **2024**, *12*, 15705.

[13] X. Yang, H. Li, A.-Y. Lu, S. Min, Z. Idriss, M. N. Hedhili, K.-W. Huang, H. Idriss, L.-J. Li, *Nano Energy* **2016**, *25*, 42.

[14] W. L. Kwong, C. C. Lee, A. Shchukarev, J. Messinger, *Chem. Commun.* **2019**, *55*, 5017.

[15] A. Li, S. Kong, C. Guo, H. Ooka, K. Adachi, D. Hashizume, Q. Jiang, H. Han, J. Xiao, R. Nakamura, *Nat. Catal.* **2022**, *5*, 109.

[16] B. Yuan, Q. Dang, H. Liu, M. G. Sendeku, J. Peng, Y. Fan, L. Cai, A. Cao, S. Chen, H. Li et al., *Nat. Commun.* **2025**, *16*, 4583.

[17] H. Liu, Z. Zhang, J. Fang, M. Li, M. G. Sendeku, X. Wang, H. Wu, Y. Li, J. Ge, Z. Zhuang et al., *Joule* **2023**, *7*, 558.

[18] W. Zhu, X. Song, F. Liao, H. Huang, Q. Shao, K. Feng, Y. Zhou, M. Ma, J. Wu, H. Yang et al., *Nat. Comm.* **2023**, *14*, 5365.

[19] S. Hao, H. Sheng, M. Liu, J. Huang, G. Zheng, F. Zhang, X. Liu, Z. Su, J. Hu, Y. Qian et al., *Nat. Nanotechnol.* **2021**, *16*, 1371.

[20] J. Kuang, B. Deng, Z. Jiang, Y. Wang, Z.-J. Jiang, *Adv. Mater.* **2023**, e2306934.

[21] L. Yao, F. Zhang, S. Yang, H. Zhang, Y. Li, C. Yang, H. Yang, Q. Cheng, *Adv. Mater.* **2024**, *36*, e2314049.

[22] H. Su, C. Yang, M. Liu, X. Zhang, W. Zhou, Y. Zhang, K. Zheng, S. Lian, Q. Liu, *Nat. Commun.* **2024**, *15*, 95.
